# Genetic variants associated with circulating liver injury markers in Mexican Americans, a population at risk for non-alcoholic fatty liver disease

**DOI:** 10.3389/fgene.2022.995488

**Published:** 2022-10-26

**Authors:** Caroline M. Sabotta, Suet-Ying Kwan, Lauren E. Petty, Jennifer E. Below, Aron Joon, Peng Wei, Susan P. Fisher-Hoch, Joseph B. McCormick, Laura Beretta

**Affiliations:** ^1^ Department of Molecular and Cellular Oncology, The University of Texas MD Anderson Cancer Center, Houston, TX, United States; ^2^ Vanderbilt Genetics Institute and Department of Genetic Medicine, Vanderbilt University Medical Center, Nashville, TN, United States; ^3^ Department of Biostatistics, The University of Texas MD Anderson Cancer Center, Houston, TX, United States; ^4^ School of Public Health, University of Texas Health Science Center at Houston, Brownsville Regional Campus, Brownsville, TX, United States

**Keywords:** AST (aspartate aminotransferase), ALT (alanine aminotransferase), NAFLD (non alcoholic fatty liver disease), liver fibrosis, hispanics

## Abstract

**Objective:** Mexican Americans are disproportionally affected by non-alcoholic fatty liver disease (NAFLD), liver fibrosis and hepatocellular carcinoma. Noninvasive means to identify those in this population at high risk for these diseases are urgently needed.

**Approach:** The Cameron County Hispanic Cohort (CCHC) is a population-based cohort with high rates of obesity (51%), type 2 diabetes (28%) and NAFLD (49%). In a subgroup of 564 CCHC subjects, we evaluated 339 genetic variants previously reported to be associated with liver injury markers aspartate aminotransferase (AST) and alanine aminotransferase (ALT) in United Kingdom and Japanese cohorts.

**Results:** Association was confirmed for 86 variants. Among them, 27 had higher effect allele frequency in the CCHC than in the United Kingdom and Japanese cohorts, and 16 had stronger associations with AST and ALT than rs738409 (*PNPLA3*). These included rs17710008 (*MYCT1*)*,* rs2519093 (*ABO*), rs1801690 (*APOH*), rs10409243 (*S1PR2*), rs1800759 (*LOC100507053*) and rs2491441 (*RGL1*)*,* which were also associated with steatosis and/or liver fibrosis measured by vibration-controlled transient elastography. Main contributors to advanced fibrosis risk were rs11240351 (*CNTN2*), rs1800759 (*LOC100507053*)*,* rs738409 (*PNPLA3*) and rs1801690 (*APOH*), with advanced fibrosis detected in 37.5% of subjects with 3 of these 4 variants [AOR = 11.6 (95% CI) = 3.8–35.3]. AST- and ALT-associated variants implicated distinct pathways (ethanol and galactose degradation *versus* antigen presentation and B cell development). Finally, 8 variants, including rs62292950 (*DNAJC13*), were associated with gut microbiome changes.

**Conclusion:** These genotype-phenotype findings may have utility in risk modeling and disease prevention in this high-risk population.

## Introduction

Hispanics in the United States are at increased risk for chronic liver diseases, in particular for non-alcoholic fatty liver disease (NAFLD) and its severe form, non-alcoholic steatohepatitis (NASH) (Guerrero et al., 2009). NAFLD is closely associated with obesity and type 2 diabetes, and its burden is predicted to increase (Younossi et al., 2019). The increased risk of NAFLD in Hispanic populations is nearly entirely attributable to the Mexican American subgroup, with moderate and severe hepatic steatosis twice as common in Mexican Americans than other Hispanics (Shaheen et al., 2021).

Aspartate aminotransferase (AST) and alanine aminotransferase (ALT) are markers of hepatocellular injury (Kleiner et al., 2019). Elevated levels of serum AST and ALT are associated with an increase in mortality (Kim et al., 2004; Kwo et al., 2017). Identifying genetic variants associated with AST and ALT levels may improve our understanding of liver disease pathophysiology and lead to risk prediction modeling for liver diseases. The use of electronic health records has increasingly been utilized in genome-wide association studies (GWAS), allowing for larger sample sizes and increased statistical power (Fairfield et al., 2021). However, GWAS specific to Hispanics are lacking. A GWAS study of AST and ALT across 5 cohorts of Mexican Americans only identified variants in patatin-like phospholipase domain-containing protein 3 (*PNPLA3*) (Young et al., 2019). A recent meta-analysis of GWAS of AST and ALT from populations in the United Kingdom (*n* = 389,565) and Japan (*n* = 162,255) identified 172 variants associated with elevated ALT and 199 variants associated with elevated AST, comprising 339 unique variants, most of them unreported (Chen et al., 2021). Among them were the well-characterized variants rs738409 in *PNPLA3* and rs58542926 in transmembrane 6 superfamily, member 2 (*TM6SF2*). The G allele of *PNPLA3* rs738409 has been found to be strongly associated with hepatic fat accumulation and the frequency of this variant is significantly higher in Hispanics (Romeo et al., 2008). The T allele of variant rs58542926 in *TM6SF2* is also known to be strongly associated with NAFLD and risk for advanced liver fibrosis (Liu et al., 2014).

A limitation of the study by Chen et al. is the low prevalence of advanced liver disease in the two cohorts. The Cameron County Hispanic Cohort (CCHC) is a population-based cohort of Hispanics in South Texas, with high rates of obesity (51%), type 2 diabetes (28%), and NAFLD (49%) (Fisher-Hoch et al., 2010; Fisher-Hoch et al., 2015; Gill et al., 2017). We also reported a 4-fold higher prevalence of advanced liver fibrosis and cirrhosis in this population compared with the general U.S. population, primarily attributable to central obesity and diabetes (Jiao et al., 2016). We therefore sought to determine in the CCHC, the genotype frequencies of the AST- and ALT-associated variants reported by Chen et al. and test their association with elevated AST and ALT levels as well as with liver steatosis and fibrosis. In addition, as the gut microbiome contributes to NAFLD progression to NASH and advanced liver fibrosis (Le Roy et al., 2013; Loomba et al., 2017; Caussy et al., 2019; Dong et al., 2020; Oh et al., 2020; Kwan et al., 2021; Sharpton et al., 2021), we also sought to determine the association of these variants with gut microbiome composition changes, if any.

## Materials and methods

### Study participants

This study includes 564 adult participants from the CCHC 18 years of age and older, randomly selected for genome-wide genotyping and for which AST and ALT measurements were available. Written informed consent was obtained from each participant and the study protocol was approved by the Committee for the Protection of Human Subjects of the University of Texas Health Science Center at Houston and MD Anderson Cancer Center. All participants underwent a comprehensive clinical exam, as well as detailed health history and demographic interview. Fasting blood samples were collected and analyzed for metabolic and lipid panels. Clinical parameters are as follows: obesity: body mass index (BMI) ≥30; pre-diabetes: fasting blood glucose of 100–125 mg/dl or HbA1c of 5.7–6.4%, with no history of diabetes medication; diabetes: fasting blood glucose ≥126 mg/dl, HbA1c ≥ 6.5%, or having a history of diabetic medication; abnormal AST: >33 U/L; abnormal ALT: >40 U/L for males and >31 U/L for females; heavy drinking: >20 g/day for men and >10 g/day for women; moderate drinking: non-zero weekly alcohol consumption, but not meeting the heavy drinking threshold; former smoking: lifetime consumption of ≥100 cigarettes while currently being a non-smoker; homeostasis model assessment (HOMA): glucose (mg/dl)/18 × insulin (mU/L)/22.5. In addition, trained operators obtained controlled attenuation parameter (CAP) measurements (dB/m) for liver steatosis and liver stiffness measurements (LSM, kiloPascals, kPa) for liver fibrosis, using vibration-controlled transient elastography (VCTE) (FibroScan^®^ 502 Touch or FibroScan^®^ 530 Compact, Echosens). Presence of liver steatosis was defined as CAP ≥268 as described (Karlas et al., 2017). Significant liver fibrosis (F2-F4) was defined as LSM ≥7.1 kPa, while advanced fibrosis (F3-F4) was defined as LSM ≥8.8 kPa, as described (Wong et al., 2010). LSM measurements were considered inconclusive if < 10 valid measures. Demographic, laboratory and clinical parameters of the study participants are described in Table 1.

**TABLE 1:** Demographic, laboratory and clinical parameters of the 564 CCHC subjects. BMI: body mass index, HbA1c: hemoglobin A1c, CAP: controlled attenuation parameter measured by Fibroscan, LSM: liver stiffness measurement measured by Fibroscan, AST: aspartate aminotransferase, ALT: alanine aminotransferase; abnormal AST: >33 U/L, abnormal ALT: >40 U/L for males and >31 U/L for females; HOMA: Homeostasis Model Assessment. Data are also shown for the subgroup of 231 CCHC subjects with 16S sequencing data available.

	Mean (range)—median or frequency (%)	
Parameter	All 564 subjects	Subgroup of 231 subjects
Male	177 (31.4%)	62 (26.8%)
Age	51.3 (18.0–89.0)—53.5	52.8 (19.0–89.0)—54.0
BMI	30.8 (18.8–61.2)—29.9	31.0 (18.8–49.0)—30.6
Overweight (≥25 BMI)	472 (86.8%)	202 (87.4%)
Obese (≥30 BMI)	271 (49.8%)	121 (52.4%)
Mean Waist	102.1 (65.0–167.0)—101.0	103.1 (74.5–137.5)—101.0
Fasting glucose (mg/dl)	107.9 (64.0–339.0)—97.0	111.1 (76.0–272.0)—97.5
HbA1c	6.1 (3.5–12.9)—5.7	6.2 (3.8–11.2)—5.8
Diabetes		
No Diabetes	153 (27.3%)	50 (21.7%)
Pre-Diabetes	232 (41.4%)	94 (40.9%)
Diabetes	175 (31.2%)	86 (37.4%)
Systolic Blood Pressure	120.2 (82.5–213.0)—116.8	120.1 (89.5–196.0)—117.0
Diastolic Blood Pressure	71.9 (50.0–109.0)—71.0	72.3 (50.0–96.0)—72.0
Smoking Status		
Never	387 (69.1%)	162 (70.4%)
Former	117 (20.9%)	51 (22.2%)
Current	56 (10.0%)	17 (7.4%)
Drinking Status		
Never	395 (70.5%)	153 (66.5%)
Moderate	144 (25.7%)	70 (30.4%)
Heavy	21 (3.8%)	7 (3.0%)
Fibroscan LSM (kPa)	5.7 (1.5–75.0)—4.5	5.6 (2.3–45.5)—4.7
Fibrosis (≥7.1 kPa)	69 (13.6%)	28 (12.6%)
Advanced Fibrosis (≥8.8 kPa)	39 (7.7%)	18 (8.1%)
Blood Tests		
High density lipoprotein (mg/dl)	49.0 (0.0–109.0)—48.0	49.8 (0.0–109.0)—49.0
Albumin (gm/dL)	4.0 (2.7–4.8)—4.0	4.0 (3.1–4.6)—4.0
Low density lipoprotein (mg/dl)	106.7 (15.0–209.0)—105.0	109.3 (15.0–204.0)—108.0
Total bilirubin (mg/L)	0.5 (0.1–1.7)—0.4	0.5 (0.1–1.5)—0.4
Triglycerides (mg/dl)	146.5 (36.0–982.5)—130.0	149.3 (36.0–494.0)—136.5
Alkaline phosphatase (U/L)	88.2 (4.0–401.0)—82.0	84.0 (21.0–164.0)—82.0
Total cholesterol (mg/dl)	184.9 (97.5–318.0)—181.5	188.8 (105.0–318.0)—184.5
Insulin (mU/L)	13.0 (0.1–73.0)—11.1	13.2 (1.4–40.3)—11.1
Platelets (x10^9^/L)	246.6 (90.0–453.0)—242.0	248.4 (126.0–426.0)—243.5
Creatinine (mg/dl)	0.8 (0.2–6.8)—0.7	0.8 (0.4–2.8)—0.7
HOMA	3.5 (0.0–26.3)—2.7	3.7 (0.5–26.3)—2.7
AST (U/L)	29.6 (8.0–249.0)—26.5	29.5 (11.0–205.0)—27.5
Abnormal AST	146 (25.9%)	58 (25.1%)
ALT (U/L)	38.4 (15.0–240.0)—32.5	38.1 (15.0–199.0)—33.0
Abnormal ALT	271 (48.0%)	113 (48.9%)

### Genome-wide genotyping

DNA was extracted from buffy coat using mini blood DNA kit (Qiagen). Genome-wide genotyping was performed using Illumina Multi-Ethnic Genotyping Array (MEGA) with 2.7 million single nucleotide polymorphisms (SNPs), optimized for the Hispanic population. After stringent pre-imputation quality control measures including SNP/subject-wise genotyping missing rate, Hardy–Weinberg equilibrium, heterozygosity rate, sample duplication and sex inconsistency, we imputed the data to the TOPMed whole genome sequencing reference panel using the Michigan Imputation Server (Das et al., 2016). Genotyping results of the variants included in this study, as well as AST and ALT measurements of each subject, are listed in Supplementary Table S1.

### Ingenuity Pathway Analysis of genes affected by aspartate aminotransferase- and alanine aminotransferase-associated variants

The database of expression quantitative trait loci (eQTL) studies from PhenoScanner (Staley et al., 2016; Kamat et al., 2019) was used to find genes with expression significantly associated (*p* < 1 × 10^−5^) with the studied variants and their proxies in linkage disequilibrium (*r* ^2^ > 0.6). Ingenuity Pathway core analysis (Ingenuity^®^ Pathway Analysis, www.qiagen.com/ingenuity) was then performed using these genes.

### Stool DNA extraction, 16S rRNA amplicon sequencing and bioinformatic analysis

Stool samples were collected using the OMNIgene^®^ GUT stool collection kit (DNA Genotek, Ontario, Canada). Subjects who had antibiotic, probiotic or proton pump inhibitor use within 30 days of stool collection, were excluded. DNA was extracted using the QIAamp Fast DNA stool mini Kit (Qiagen). The V4 region of the bacterial 16S rRNA gene was amplified by PCR (primer sequences listed in Supplementary Table S2). Libraries were purified using Zymo I-96 columns and analyzed on the 4,200 Tapestation system (Agilent). Barcoded amplicons were pooled in equal concentrations. Pooled libraries were quantified by Qubit fluorometer and the molarity was calculated based on amplicon size. Sequencing was performed using 250bp paired-end on the Illumina MiSeq platform (primer sequences listed in Supplementary Table S2). Paired-end reads were de-multiplexed and split in QIIME. Merging of paired-end reads to create consensus sequences was done by VSEARCH v7, allowing up to a maximum of 10 mismatched. The cluster_otus command, an implementation of UPARSE algorithm, was used to perform 97% related operational taxonomic units (OTU) clustering. Denoising was done by the unoise3 command. OTUs were subjected to taxonomy assignment using the Mothur with Silva database (v138).

### Statistical analyses

All statistical analyses were performed in R v4.1.2 unless specified otherwise. Pairwise correlations were performed using Spearman’s correlation. Using the “glm.fit” function, logistic regression was performed to determine the association between each variant as the independent variable and selected clinical parameters as the binary dependent variable. Odds ratios were adjusted for age and gender (AOR). Principal coordinates analysis (PCoA) was performed using the “cmdscale” function and the weighted UniFrac distances of the OTU tables. Permutational multivariate analysis of variance (PERMANOVA) tests were performed with the Vegan package using weighted UniFrac distances. Differences in bacterial abundance between genotypes were assessed using the linear discriminant analysis (LDA) effect size (LefSe) tool (Segata et al., 2011), with *p* < 0.05 and log10 LDA score >2 considered significant. Taxa with ≥0.1% abundance in at least 10% of samples were included. Cladograms showing the phylogenetic relationships between bacteria enriched in each genotype group were created using the Huttenhower lab galaxy server (Afgan et al., 2018). We considered *p* < 0.05 as significant for all statistical tests. Two machine learning algorithms, ridge regression and random forest, were implemented using the “caret” package, with the variants as independent variables and advanced fibrosis as the binary outcome. For ridge regression, the method “glmnet” was used, with an alpha of 0. Using 5-fold cross validation, the optimal lambda giving the maximum area under the receiver operating characteristic curve (AUC) was determined. Standardized coefficients at the optimal lambda were generated to obtain rankings of importance. For random forest, the method “rf” was used. Using 5-fold cross validation, the optimal “mtry” value giving the maximum AUC was determined. Unscaled permutation-based importance scores (mean decrease in accuracy) at the optimal “mtry” were generated to obtain rankings of importance.

## Results

### Frequency of reported aspartate aminotransferase- and alanine aminotransferase-associated variants in the Cameron County Hispanic Cohort

We performed genome-wide genotyping using genomic DNA from 564 randomly selected subjects from the CCHC (Table 1). The average age was 51.3 and 177 (31.4%) of the subjects were male. Among all participants, 271 (49.8%) were obese and 175 (31.2%) had diabetes. The study cohort included 146 subjects (25.9%) with elevated AST levels and 271 subjects (48.0%) with elevated ALT levels. All subjects were screened for liver steatosis and fibrosis using VCTE. VCTE screening identified 297 subjects (58.7%) with steatosis (CAP ≥268) and 69 subjects (13.6%) with significant liver fibrosis (kPa ≥7.1). AST and ALT values in the study participants positively correlated with HOMA values (AST r = 0.24 *p* < 0.001; ALT r = 0.36 *p* < 0.001) and liver fibrosis kPa values (AST r = 0.22 *p* < 0.001; ALT r = 0.25 *p* < 0.001) (Supplementary Figures S1A,B). ALT values also correlated with steatosis CAP values (r = 0.28 *p* < 0.001) (Supplementary Figure S1B).

We calculated the frequency in the CCHC of the effect allele for the 339 variants reported to be associated with an increase in AST or ALT in the United Kingdom Biobank (UKBB) and BioBank Japan (BBJ) cohorts (Chen et al., 2021). We detected 329 of these 339 variants in the 564 CCHC participants. The effect allele frequency (EAF) for these 329 variants in the CCHC is shown in Supplementary Table S3. Among them, 82 variants had a higher EAF in the CCHC than in the UKBB and BBJ cohorts. The EAF for 38 variants was higher by 10% or more. As anticipated, rs738409 (*PNPLA3*) was among these 38 variants, with an EAF of 52% in CCHC compared to 22% in UKBB and 45% in BBJ. Important increases in EAF were also observed for rs2126259 in the noncoding RNA *LOC157273* (CCHC: 32%, UKBB: 10%, BBJ: 1%), rs61352607 in inhibin beta C (*INHBC*) (CCHC: 42%, UKBB: 24%, BBJ: 7%), rs73545546 in dermatin actin binding protein (*DMTN*) (CCHC: 27%, UKBB: 16%, BBJ: 5%), rs76722284 near long intergenic ncRNA 1336 (*LINC01336*) and ankyrin repeat domain-containing protein 31 (*ANKRD31*) (CCHC: 24%, UKBB: 4%, BBJ: 10%), rs7209484 near nuclear factor erythroid 2-like 1 (*NFE2L1*) and chromobox 1 (*CBX1*) (CCHC: 40%, UKBB: 24%, BBJ: 22%) and rs17710008 in Myc target in myeloid cells 1 (*MYCT1*) (CCHC: 23%, UKBB: 19%, and BBJ: 12%).

### Replication of aspartate aminotransferase and alanine aminotransferase association in the Cameron County Hispanic Cohort

**FIGURE 1:**
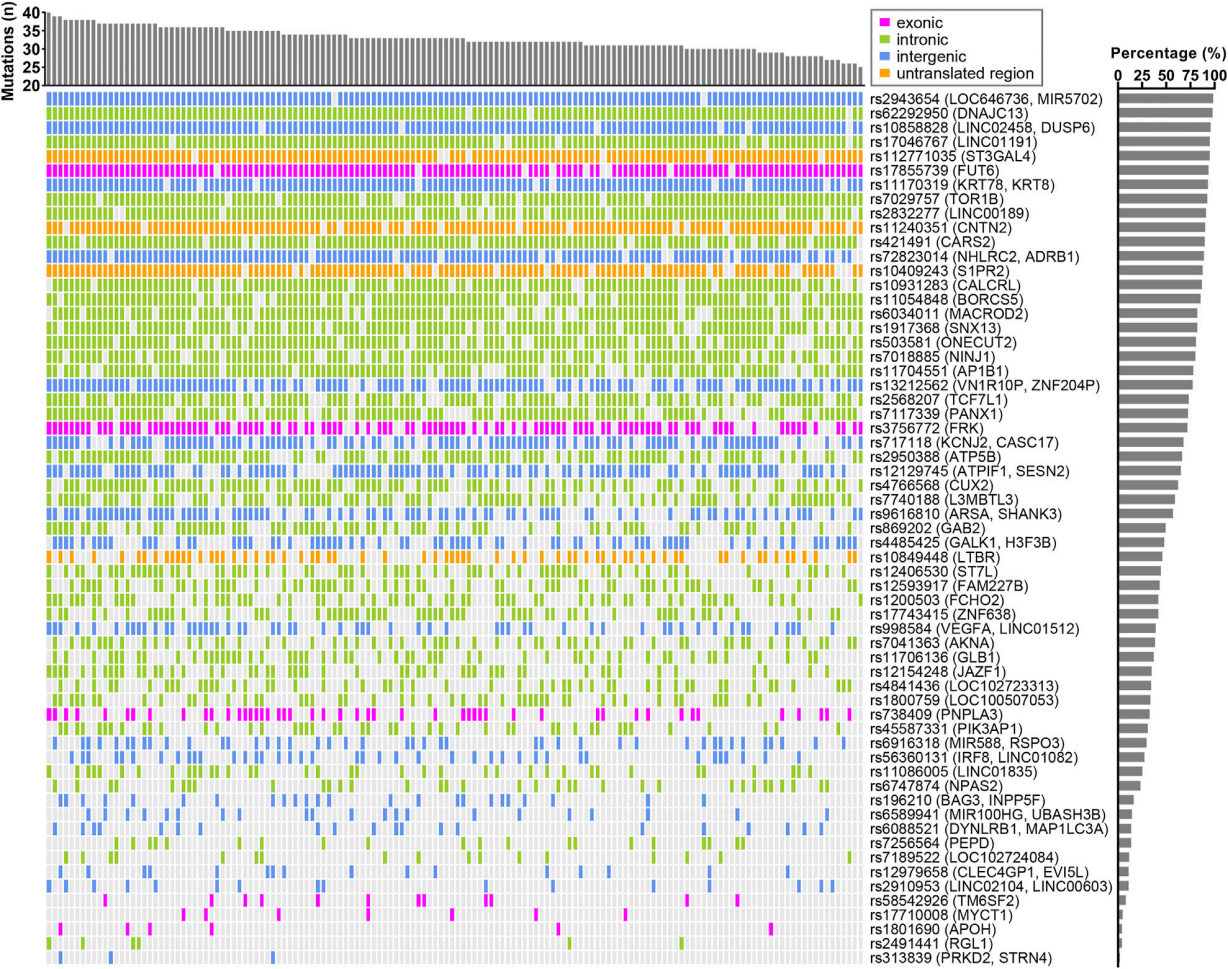
Landscape of AST-associated variants in CCHC subjects with elevated AST. The bar chart at the top represents the number of variants in each subject. The bar chart on the right shows the frequency of each variant in those CCHC subjects with elevated AST levels. Each column represents one subject and each row represents one variant.

**FIGURE 2:**
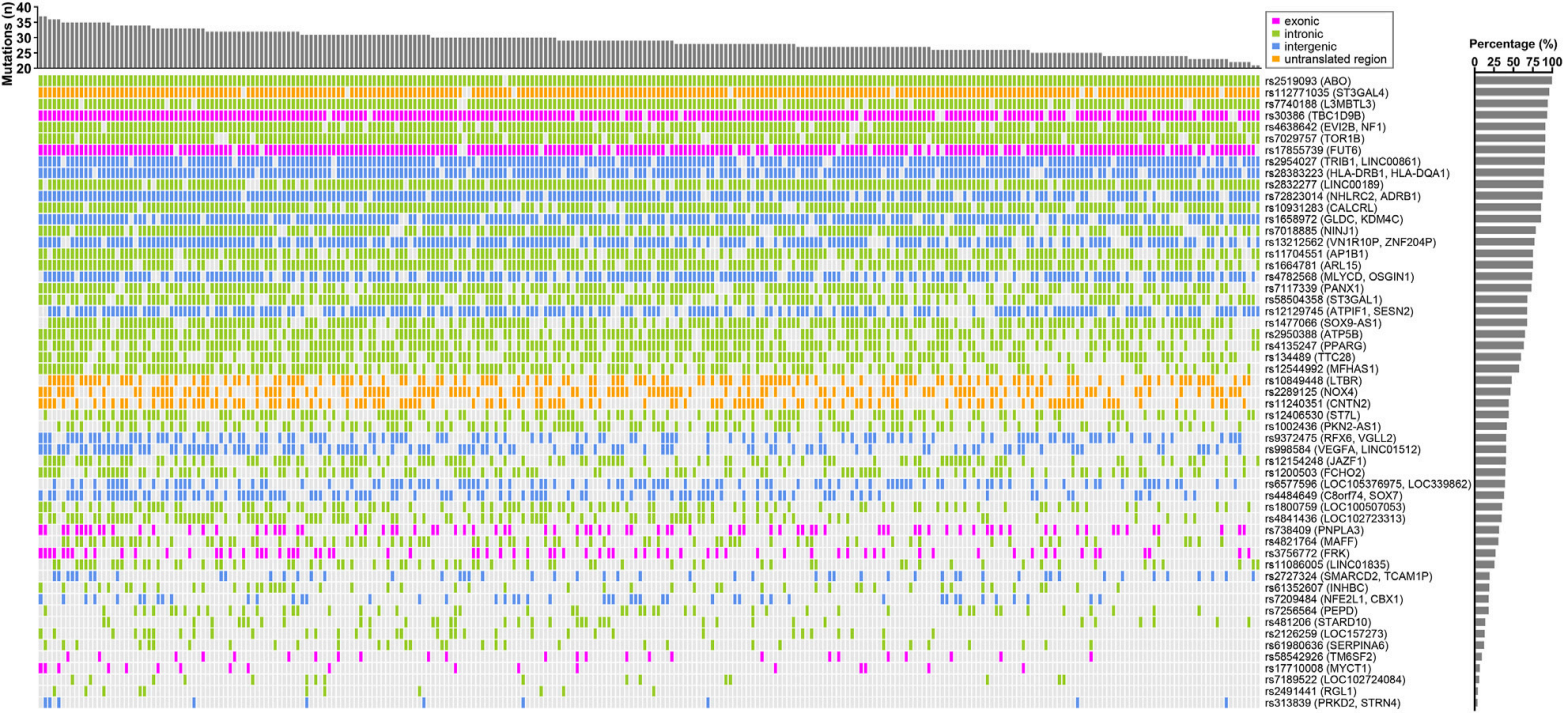
Landscape of ALT-associated variants in CCHC subjects with elevated ALT levels. The bar chart at the top represent the number of variants in each subject. The bar chart on the right shows the frequency of each variant in those CCHC subjects with elevated ALT levels. Each column represents one subject and each row represents one variant.

We used logistic regression analysis, adjusting for age and gender, to determine possible associations between each of the 329 variants and elevated AST or ALT levels in the CCHC. After exclusion of variants with *r* ^2^ > 0.8 in linkage disequilibrium and selecting for variants with the same effect allele in the CCHC as in the UKBB and BBJ cohorts, 86 variants were found associated with elevated AST (n = 61) and/or ALT (n = 55) in the CCHC (Supplementary Table S4). As anticipated, rs738409 (*PNPLA3*) and rs58542926 (*TM6SF2*) were among them. Other variants resulting in non-synonymous amino acid changes, included rs17710008 (*MYCT1*)*,* rs1801690 in apoliproprotein H (*APOH*), rs17855739 in fucosyltransferase 6 (*FUT6*), rs3756772 in FYN-related SRC family tyrosine kinase (*FRK*) and rs30386 in TBC1 domain family member 9B (*TBC1D9B*). The median number of variants detected in the 146 subjects with elevated AST levels was 33, ranging from 25 to 40 (Figure 1), while the median number of variants detected in the 271 subjects with elevated ALT levels was 29, ranging from 21 to 37 (Figure 2).

Among the 86 AST- and ALT-associated variants in the CCHC, 27 displayed an EAF that was higher in the CCHC than in both the UKBB and BBJ cohorts, including 13 with EAF higher by more than 10%. These included rs738409 (*PNPLA3*) found to be associated with both AST [AOR = 2.12 (1.44–3.12), *p* < 0.001] and ALT [AOR = 3.28 (1.85–5.80), *p* < 0.001]. Other variants with >10% EAF in the CCHC and associated with both elevated AST and ALT included rs2950388 in ATP synthase F1 subunit beta (*ATP5B*) [AST AOR = 1.54 (1.03–2.33), *p* = 0.034; ALT AOR = 1.75 (1.22–2.56), *p* = 0.003], rs7018885 in nerve injury-induced protein 1 (*NINJ1*) [AST AOR = 1.75 (1.15–2.70), *p* = 0.009; ALT AOR = 2.17 (1.33–3.57), *p* = 0.002], rs1200503 in FCH domain only protein 2 (*FCH O 2*) [AST AOR = 1.41 (0.98–2.00), *p* = 0.050; ALT AOR = 1.52 (1.03–2.2), *p* = 0.037], rs1800759 in the non-coding RNA *LOC100507053* [AST AOR = 1.49 (1.00–2.22), *p* = 0.050; ALT AOR = 1.64 (1.14–2.38), *p* = 0.009] and rs17710008 (*MYCT1*) [AST AOR = 2.35 (1.07–5.16), *p* = 0.033; ALT AOR = 3.20 (1.43–7.15), *p* = 0.005]. Of note, rs17710008 (*MYCT1*) had stronger or similar associations with AST and ALT compared to rs738409 (*PNPLA3*)*.* Variants with EAF higher by >10% and associated with only AST or ALT included rs17743415 in zinc finger protein 638 (*ZNF638*) [AST AOR = 1.50 (1.02–2.20), *p* = 0.041], rs28383223 near major histocompatibility complex class II DR beta-1 and DQ alpha-1 (*HLA-DRB1, HLA-DQA1*) [ALT AOR = 2.35 (1.26–4.41), *p* = 0.007], rs4638642 in ecotropic viral integration site 2B (*EVI2B*) and neurofibromin 1 (*NF1*) [ALT AOR = 2.18 (1.30–3.66), *p* = 0.003], rs61352607 (*INHBC*) [ALT AOR = 1.93 (1.20–3.11), *p* = 0.007], rs2126259 (*LOC157273*) [ALT AOR = 1.96 (1.10–3.45), *p* = 0.022], rs2954027 near tribbles pseudokinase 1 (*TRIB1*) and long intergenic ncRNA 861 (*LINC00861*) [ALT AOR = 1.89 (1.13–3.15), *p* = 0.015] and rs7209484 (*NFE2L1*, *CBX1*) [ALT AOR = 1.62 (1.01–2.59), *p* = 0.045]. These results are described in Supplementary Table S5.

**FIGURE 3:**
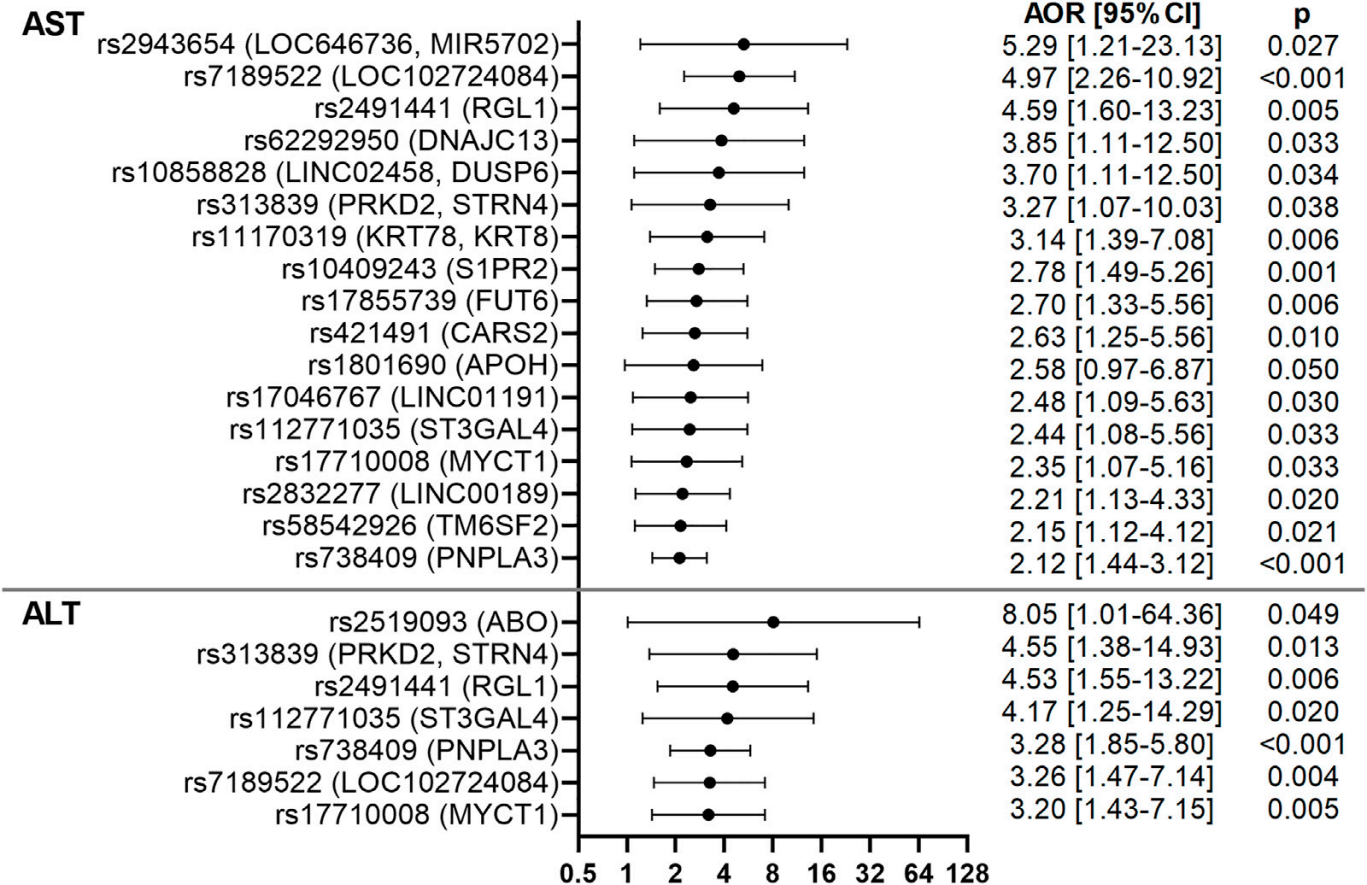
Forest plots of AST- and ALT-associated variants in the CCHC. Forest plots show AORs [95% CI] for elevated AST and ALT, after adjusting for age and gender. The top variants associated with AST and ALT are shown.

In addition to rs17710008 (*MYCT1*), 4 variants had stronger or similar associations with elevated AST and ALT levels than rs738409 (*PNPLA3*)*.* These included rs7189522 (*LOC102724084*) [AST AOR = 4.97 (2.26–10.92), *p* < 0.001; ALT AOR = 3.26 (1.47–7.14), *p* = 0.004], rs313839 near protein kinase D2 (*PRKD2*) and striatin, calmodulin-binding protein 4 (*STRN4*) [AST AOR = 3.27 (1.07–10.03), *p* = 0.038; ALT AOR = 4.55 (1.38–14.93), *p* = 0.013], rs2491441 in ral guanine nucleotide dissociation stimulator-like 1 (*RGL1*) [AST AOR = 4.59 (1.60–13.23), *p* = 0.005; ALT AOR = 4.53 (1.55–13.22), *p* = 0.006] and rs112771035 near ST3 beta-galactoside alpha-2,3-sialyltransferase 4 (*ST3GAL4*) [AST AOR = 2.44 (1.08–5.56), *p* = 0.033; ALT AOR = 4.17 (1.25–14.29), *p* = 0.020]. Eleven additional variants had stronger associations with elevated AST levels than rs738409 (*PNPLA3*) (Figure 3). These included rs1801690 (*APOH*) [AOR = 2.58 (0.97–6.87), *p* = 0.050], rs421491 in cysteinyl-tRNA synthetase 2 (*CARS2*) [AOR = 2.63 (1.25–5.56), *p* = 0.010], rs62292950 in DNAJ/HSP40 homolog, subfamily C, member 13 (*DNAJC13*) [AOR = 3.85 (1.11–12.50), *p* = 0.033], rs17855739 (*FUT6*) [AOR = 2.70 (1.33–5.56), *p* = 0.006], rs11170319 near keratin 78 (*KRT78*) and keratin 8 type II (*KRT8*) [AOR = 3.14 (1.39–7.08), *p* = 0.006], rs2832277 (*LINC00189*) [AOR = 2.21 (1.13–4.33), *p* = 0.020], rs17046767 (*LINC01191*) [AOR = 2.48 (1.09–5.63), *p* = 0.030], rs10858828 near *LINC02458* and dual-specificity phosphatase 6 (*DUSP6*) [AOR = 3.70 (1.11–12.50), *p* = 0.034], rs2943654 near *LOC646736* and *MIR5702* [AOR = 5.29 (1.21–23.13), *p* = 0.027], rs10409243 in sphingosine-1-phosphate receptor 2 (*S1PR2*) [AOR = 2.78 (1.49–5.26), *p* = 0.001] and rs58542926 (*TM6SF2*) [AOR = 2.15 (1.12–4.12), *p* = 0.021]. One additional variant had stronger association with elevated ALT levels than rs738409 (*PNPLA3*)*,* rs2519093 in the glycosyltransferase *ABO* gene [AOR = 8.05 (1.01–64.36), *p* = 0.049]. These results are shown in Figure 3; Supplementary Table S6.

### Genotype-phenotype associations for aspartate aminotransferase- and alanine aminotransferase-associated variants in the Cameron County Hispanic Cohort

**FIGURE 4:**
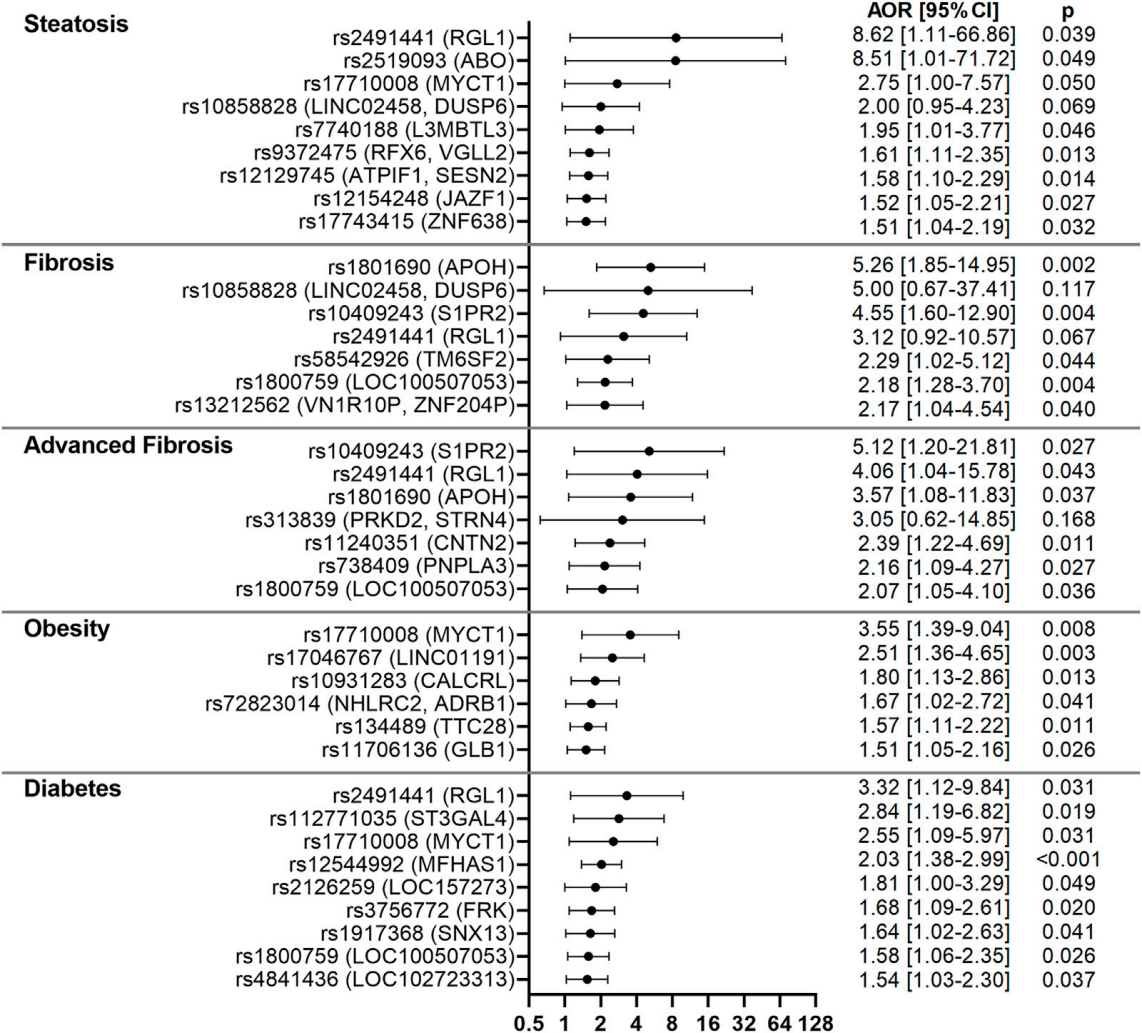
Forest plots for variants associated with NAFLD-related clinical parameters in the CCHC. Forest plots show AORs [95% CI] for steatosis, liver fibrosis, advanced fibrosis, obesity and diabetes, after adjusting for age and gender. The top variants associated with each outcome are shown.

To determine the role of the identified variants on NAFLD liver pathologies and associated co-morbidities, all subjects were screened with VCTE to detect and stage liver fibrosis and steatosis. Logistic regression analysis, adjusting for age and gender, identified associations with presence of steatosis, liver fibrosis or advanced liver fibrosis for 17 of the 86 variants. Associations with diabetes or obesity were also observed for 14 of the 86 variants. Only rs2491441 (*RGL1*) was significantly associated with both steatosis and advanced fibrosis and both associations were very strong [AORs = 8.62 (1.11–66.86), *p* = 0.039 and 4.06 (1.04–15.78), *p* = 0.043]. rs2491441 (*RGL1*) was also associated with diabetes. Strong associations with steatosis were observed for rs2519093 (*ABO*) [AOR = 8.51 (1.01–71.72), *p* = 0.049] and rs17710008 (*MYCT1*) [AOR = 2.75 (1.00–7.57), *p* = 0.050]. In addition, rs17710008 (*MYCT1*) was associated with both obesity and diabetes [AORs = 3.55 (1.39–9.04), *p* = 0.008 and 2.55 (1.09–5.97), *p* = 0.031]. Strong associations with both liver fibrosis and advanced fibrosis were observed for rs1801690 (*APOH*) [AORs = 5.26 (1.85–14.95), *p* = 0.002 and 3.57 (1.08–11.83), *p* = 0.037], rs10409243 (*S1PR2*) [AORs = 4.55 (1.60–12.90), *p* = 0.004 and 5.12 (1.20–21.81), *p* = 0.027] and rs1800759 (*LOC100507053*) [AORs = 2.18 (1.28–3.70), *p* = 0.004 and 2.07 (1.05–4.10), *p* = 0.036]. rs738409 (*PNPLA3*) associated with advanced fibrosis [AOR = 2.16 (1.09–4.27), *p* = 0.027), while rs58542926 (*TM6SF2*) associated with fibrosis [AOR = 2.29 (1.02–5.12), *p* = 0.044]. These results are shown in Figure 4; Supplementary Table S6.

**FIGURE 5:**
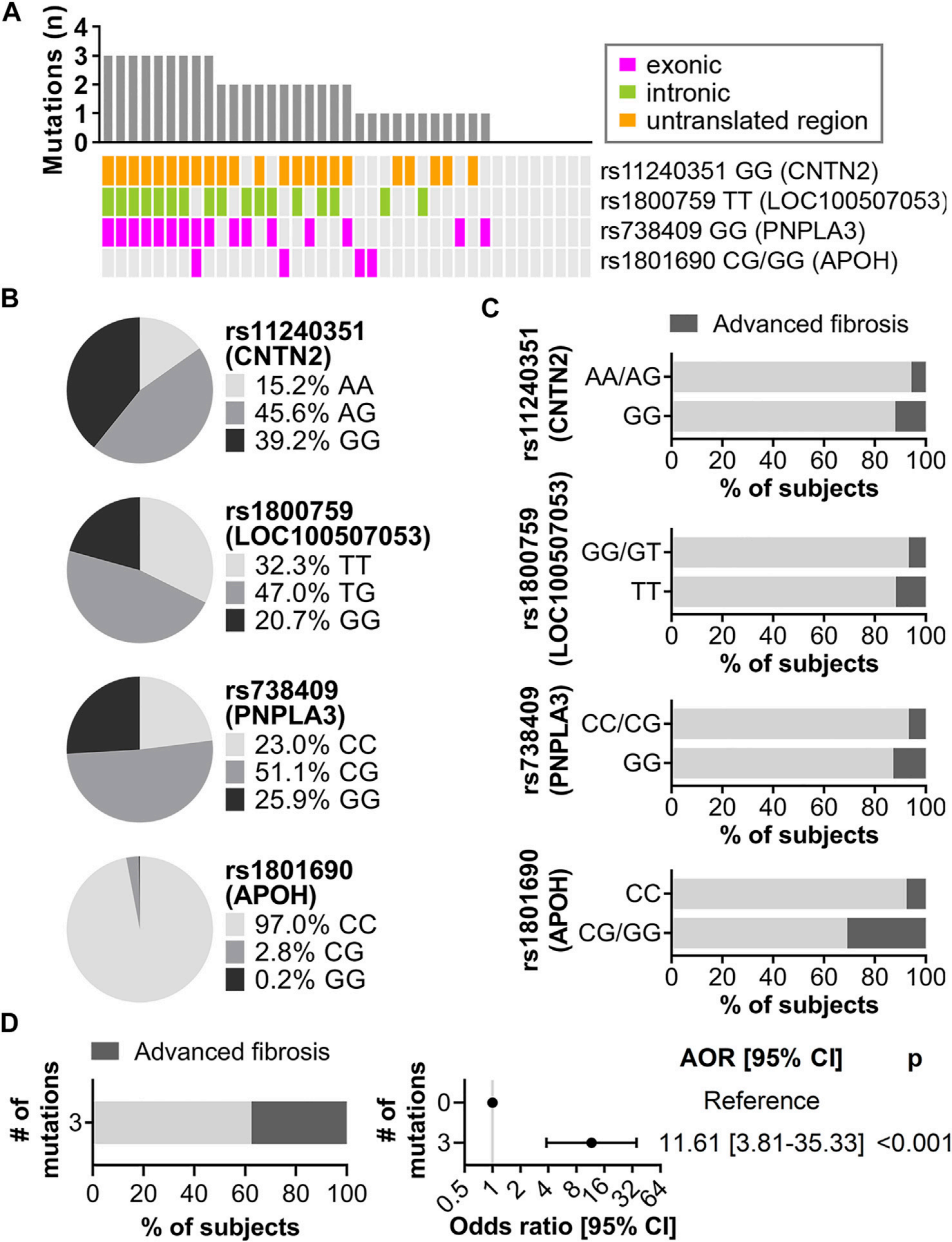
Main contributors to risk of advanced liver fibrosis identified by ridge regression and random forest. **(A)** Mutation landscape of the top four variants in CCHC subjects with advanced fibrosis. The bar chart represents the number of variants in each subject. Each column represents one subject and each row represents one variant. **(B)** Genotype frequency of the four variants in the CCHC cohort. **(C)** Percentage of subjects with advanced fibrosis by genotype. **(D)** Left, percentage of subjects with advanced fibrosis in subjects with three of the four variants. Right, forest plot showing AOR [95% CI] for advanced liver fibrosis in subjects with three of the four variants.

To determine which of the 86 AST- and ALT-associated variants contributed the most to risk of advanced fibrosis, two machine learning algorithms, ridge regression and random forest, were implemented (Supplementary Figure S2A). Based on standardized coefficients from ridge regression and unscaled permutation-based importance scores from random forest, four variants were ranked among the top 10 contributors in both methods: rs11240351 GG in contactin 2 (*CNTN2*), rs1800759 TT (*LOC100507053*)*,* rs738409 GG (*PNPLA3*) and rs1801690 CG/GG (*APOH*), present in 59.0%, 43.6%, 41.0%, and 10.3% of subjects with advanced fibrosis, respectively (Figure 5A). At least one of these four variants was detected in 31 (79.5%) of the 39 subjects with advanced fibrosis (Figure 5A). The genomic context including overlapping and surrounding genes for these four variants is shown in Supplementary Figure S2B. The frequency of each variant in the CCHC is shown in Figure 5B while Figure 5C shows the frequency of advanced liver fibrosis in subjects with and without the variants. A significant increase in risk of advanced fibrosis was observed for those subjects with at least three out of the four variants, with a frequency of 37.6% and [AOR = 11.6 (3.8–35.3), *p* < 0.001], after adjusting for age and gender (Figure 5D). Further adjusting with alcohol intake in addition to age and gender did not change the estimated AOR. Confirming an additive effect, the sum of effect alleles present from the four variants was significantly associated with an increased risk of advanced fibrosis, with AOR = 1.62 (1.23–2.13) (*p* = 0.001) for every additional effect allele present.

### Biological functions and gut microbiome changes associated with aspartate aminotransferase- and alanine aminotransferase-associated variants in the Cameron County Hispanic Cohort

**FIGURE 6:**
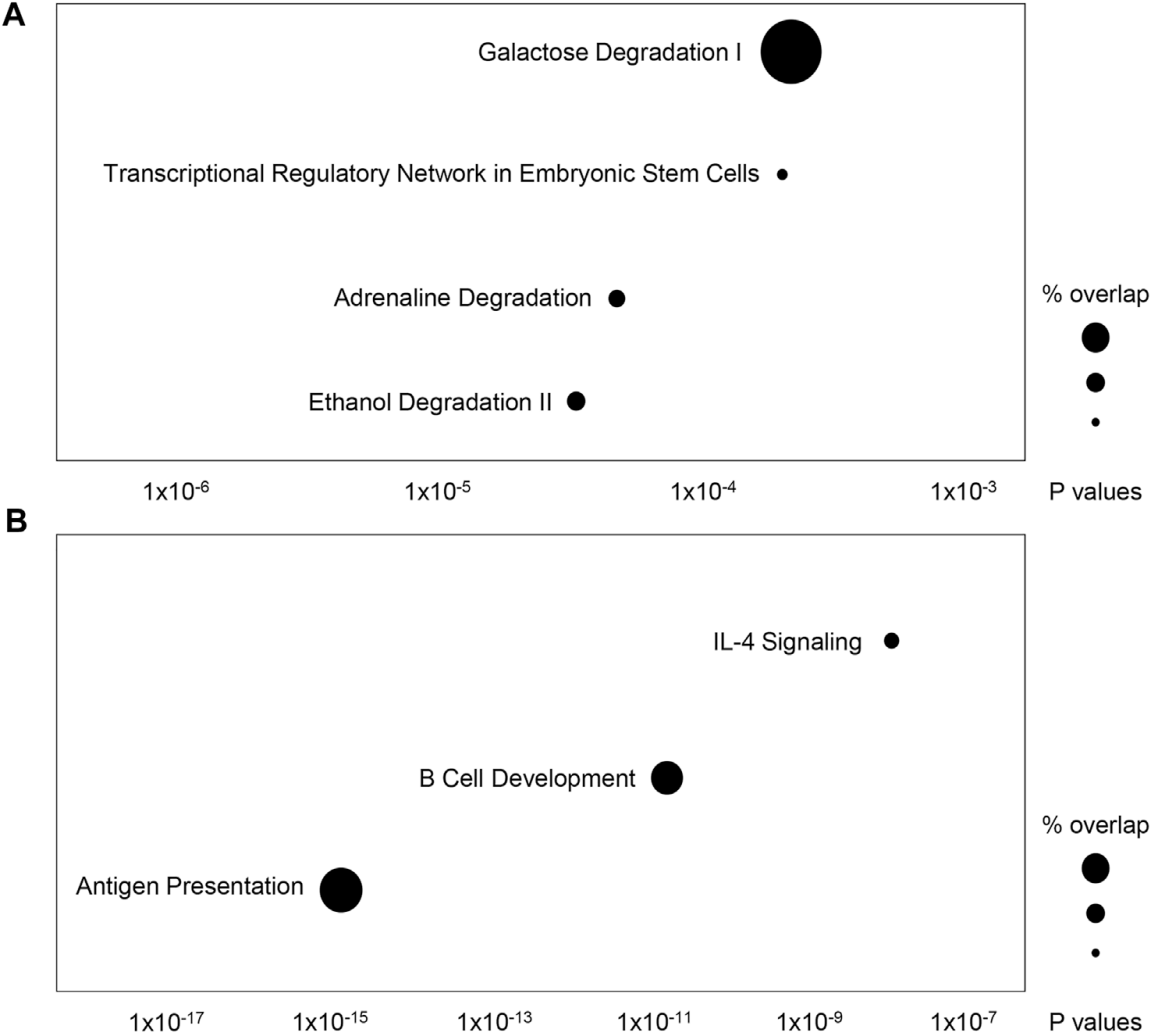
Ingenuity Pathway Analysis of genes regulated by AST- and ALT-associated variants in the CCHC. The top canonical pathways overrepresented by genes regulated by **(A)** AST-associated variants and their proxies, and **(B)** ALT- associated variants and their proxies.

Using eQTL data from PhenoScanner, we identified gene expression changes modulated by the 86 AST- and ALT-associated variants in the CCHC and their proxies (Supplementary Table S7). With gene expression changes modulated by AST-associated variants, Ingenuity Pathway Analysis identified an enrichment in the following canonical pathways: ethanol degradation II (*p* = 5.8 × 10^−5^), adrenaline degradation (*p* = 8.1 × 10^−5^), transcriptional regulatory network in embryonic stem cells (*p* = 3.6 × 10^−4^) and galactose degradation I (*p* = 3.8 × 10^−4^) (Figure 6A). The same analysis for ALT identified an enrichment in the following canonical pathways: antigen presentation pathway (*p* = 2.5 × 10^−15^), B cell development (*p* = 2.2 × 10^−11^) and interleukin 4 (IL-4) signaling (*p* = 2.3 × 10^−7^) (Figure 6B).

**FIGURE 7:**
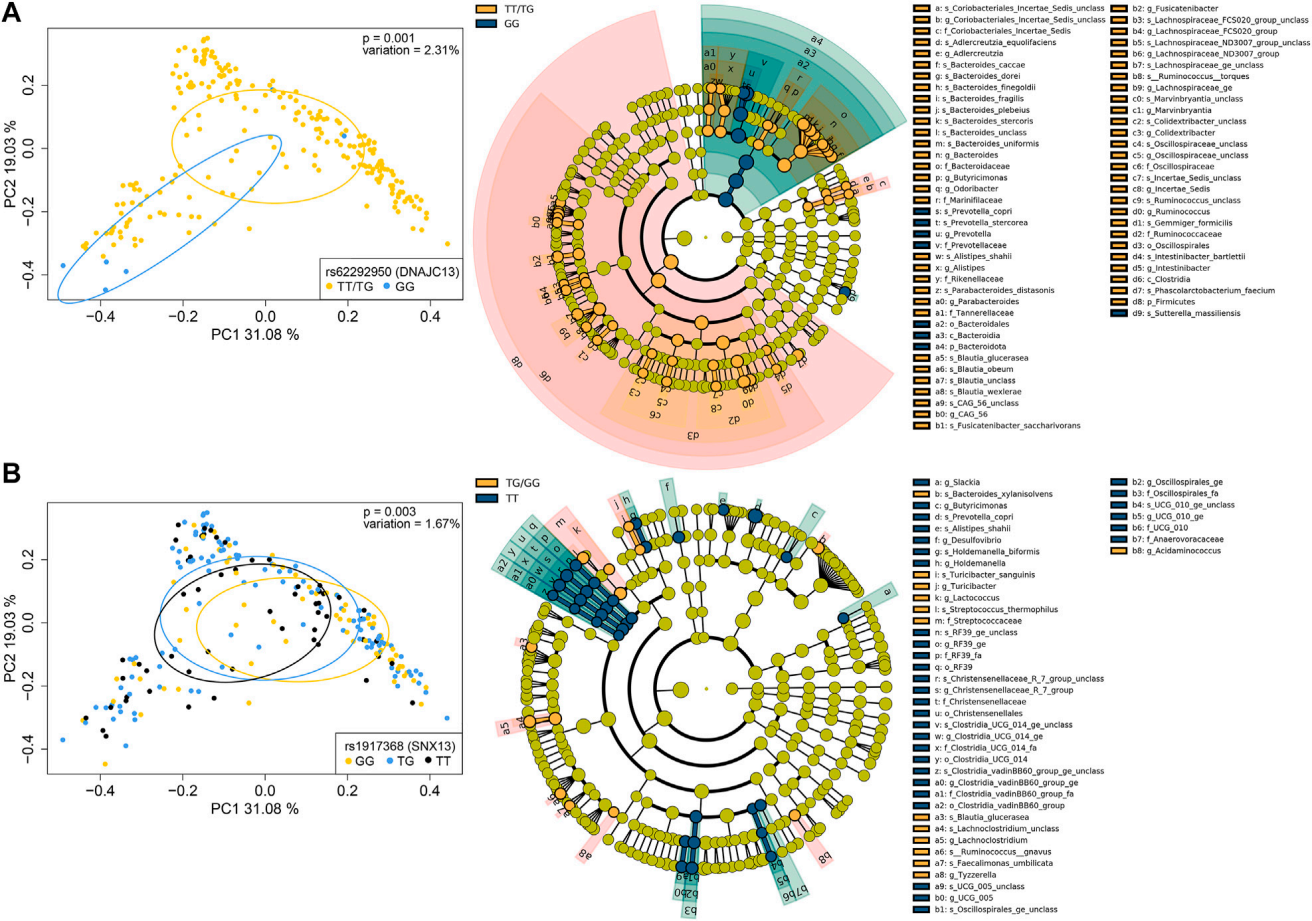
Genotype-associated gut microbiome changes. **(A)** PCoA plots of microbiome profiles, based on weighted UniFrac distances, with samples grouped by the TT/TG and GG genotypes for rs62292950 (DNAJC13) (left) and cladogram showing taxa with significantly different abundances between subjects with the TT/TG and GG genotypes, assessed by LEfSe (right). **(B)** PCoA plot of microbiome profiles with samples grouped by the GG, TG, and TT genotypes for rs1917368 (SNX13) (left) and cladogram showing taxa with significantly different abundances between subjects with the TG/GG and TT genotypes (right). Bacterial classifications at the phylum (p_), class (c_), order (o_), family (f_), genus (g_), and species (s_) levels are shown.

In addition, 16S sequencing was performed and gut taxonomic composition was determined in 231 of the 564 subjects for which stool samples were available. To assess potential effects of the AST- or ALT-associated variants in the CCHC on gut microbiome profiles, we performed principal component and PERMANOVA analyses using weighted UniFrac distances. Among the 86 variants analyzed, 8 (9.3%) were found to be significantly associated with a shift in gut microbiome profiles. The two variants with the strongest variation effect on gut microbiome included rs62292950 (*DNAJC13*) (2.31% variation, *p* = 0.001) and rs1917368 in sorting nexin 13 (*SNX13*) (1.67% variation, *p* = 0.003) (Figure 7). Others included rs2727324 near SWI/SNF-related matrix-associated actin-dependent regulator of chromatin subfamily D member 2 (*SMARCD2*) and testicular cell adhesion molecule 1 (*TCAM1P*) (1.22% variation, *p* = 0.023), rs503581 in one cut homeobox 2 (*ONECUT2*) (1.56% variation, *p* = 0.014), rs7256564 in peptidase D (*PEPD*) (1.19% variation, *p* = 0.037), rs313839 (*PRKD2*, *STRN4*) (1.15% variation, *p* = 0.038), rs7041363 in at-hook transcription factor (*AKNA*) (1.57% variation, *p* = 0.006) and rs1658972 near glycine decarboxylase (*GLDC*) and lysine demethylase 4C (*KDM4C*) (0.97% variation, *p* = 0.048) (Supplementary Figure S3). LEfSe analysis was performed to identify the specific taxonomic changes from phylum to species, associated with each of these 8 variants. While a large variation in taxonomic changes was observed across the 6 AST-associated and 4 ALT-associated variants, a trend towards increased Firmicutes/Bacteroidetes ratio was observed for 5 of the 8 variants, reaching significance for rs62292950 (*DNAJC13*) (FC = 9.25, *p* = 0.014) and rs7041363 (*AKNA*) (FC = 3.55, *p* = 0.050) (Figure 7; Supplementary Figure S3).

## Discussion

Mexican Americans are disproportionately affected by NAFLD, liver fibrosis, cirrhosis, and hepatocellular carcinoma (HCC). In a population-based Hispanic cohort in South Texas, we aimed to validate 339 genetic variants identified in a large meta-analysis of GWAS to be associated with elevated AST and/or ALT levels in cohorts from the United Kingdom and Japan (Chen et al., 2021). Unlike the UKBB and BBJ cohorts, the CCHC exhibits a high prevalence of chronic liver diseases, particularly NAFLD and liver fibrosis. We identified 86 of these 339 variants to be significantly associated with elevated AST and/or ALT levels in the CCHC, with the same effect allele as in UKBB and BBJ cohorts, demonstrating robust associations across ancestries for these variants. Interestingly, 27 of these 86 variants had a higher EAF in CCHC than in UKBB and BBJ, including 14 with an EAF higher by >10%. rs738409 (*PNPLA3*) was among these 14 variants in agreement with prior studies. Other variants with >10% EAF in CCHC and associated with both elevated AST and ALT included rs1800759 (*LOC100507053*) and rs17710008 (*MYCT1*). The locus *LOC100507053* has been reported to be associated with alcohol dependence. (Gelernter et al., 2014; Xu et al., 2015). In addition, rs1800759 (*LOC100507053*) is associated with expression regulation of the nearby alcohol dehydrogenase 4 (ADH4), 5 (ADH5) and 6 (ADH6) genes, based on eQTL data from PhenoScanner. *MYCT1* is a regulator of hematopoietic stem cell repopulation (Holmfeldt et al., 2016). Remarkably, rs17710008 (*MYCT1*) had stronger associations with AST and ALT compared to rs738409 (*PNPLA3*). Variants with EAF higher by >10% and associated with ALT included rs2126259 (*LOC157273*)*.* This variant is in linkage disequilibrium (LD) with rs4841132 reported to be associated with risk for type 2 diabetes (Manning et al., 2020).

In addition to rs17710008 (*MYCT1*), four variants had stronger associations with both elevated AST and ALT levels than rs738409 (*PNPLA3*)*.* These included rs313839 (*PRKD2*, *STRN4*). The *PRKD2* gene is involved in cell survival, migration, differentiation and proliferation pathways. Inhibition of *STRN4* suppresses the tumorigenicity of HCC cell lines (Czauderna et al., 2021). We also found rs313839 (*PRKD2*, *STRN4*) to be associated with gut microbiome changes. Interestingly, PRKD2 inhibition has been shown to affect gut microbiota in mice (Trujillo-Viera et al., 2021). rs2519093 (*ABO*) had stronger association with ALT levels than rs738409 (*PNPLA3*). The glycosyltransferase ABO gene catalyzes the formation of the antigenic structures of the ABO blood type, and non-O blood types are associated with increased NAFLD risk (Zhong et al., 2019). The *ABO* locus is also the main determinant of circulating levels of E-selectin (Polfus et al., 2019; Teng et al., 2021). E-selectin plays a key role in the recruitment of leukocytes during inflammatory processes. Increased circulating levels and expression of E-selectin in hepatic and adipose tissues have been associated with NAFLD severity (Simons et al., 2020; Rodrigues et al., 2022) and *in vivo* studies have demonstrated E-selectin’s contribution to the NASH phenotype (Rodrigues et al., 2022). Associations with AST levels, greater than that of rs738409 (*PNPLA3*), were found for rs62292950 (*DNAJC13*), rs10858828 (*LINC02458*, *DUSP6*), rs58542926 (*TM6SF2*), rs10409243 (*S1PR2*)*,* and the two exonic variants, rs1801690 (*APOH*) and rs17855739 (*FUT6*). The missense variant rs1801690 (*APOH*) has been shown to account for up to 14% of plasma level variations of the glycoprotein *APOH,* involved in lipoprotein metabolism (Mehdi et al., 1999; Mather et al., 2016). *DNAJC13* plays a role in vesicle formation and trafficking. Associations between increased levels of *DUSP6* and HCC or liver cirrhosis have been reported (Wu et al., 2022). *S1PR2* inhibition mitigated liver fibrosis development and *S1PR2* blockage has been shown to accelerate HCC progression in mice (Yoshida et al., 2020; Kawai et al., 2021). Hepatic expression of *FUT6* is significantly higher in patients with NASH compared to NAFLD patients (Ogawa et al., 2020). Of additional interest was the association of rs17743415 (*ZNF638*) with AST levels. *ZNF638* contributes to lipogenesis-associated HCC and its expression is increased in NAFLD patients (Ni et al., 2020).

A strength of this study was the implementation of VCTE screening for the detection and staging of steatosis and liver fibrosis. Several AST- and ALT-associated variants in the CCHC also had significant associations with liver fibrosis. Of note were rs2491441 (*RGL1*), rs1801690 (*APOH*)*,* rs10409243 (*S1PR2*) and rs1800759 (*LOC100507053*)*,* with AORs comparable or greater than that observed for rs738409 (*PNPLA3*) and rs58542926 (*TM6SF2*). Two machine learning algorithms confirmed separately the importance of rs738409 (*PNPLA3*) in predicting risk of advanced liver fibrosis. They also revealed the importance of rs11240351 (*CNTN2*), rs1800759 (*LOC100507053*) and rs1801690 (*APOH*) in risk prediction of advanced liver fibrosis*.* The sum of effect alleles across all four variants was significantly associated with increased risk and over a third of subjects with three out of the four variants had advanced liver fibrosis.

Interestingly, different canonical pathways were found enriched in genes regulated by AST- and ALT-associated variants, suggesting different mechanisms of liver damage. While ethanol, adrenaline and galactose degradation pathways were enriched in AST-variants regulated genes, novel associations, all affecting immune responses, were identified for ALT-variants regulated genes. These included B cell development, antigen presentation and IL-4 signaling pathways. Several functions of B cells, such as the production of anti- and pro-inflammatory cytokines and antigen presentation for T cell activation, can exacerbate hepatic tissue damage and fibrosis (Patel et al., 2021). B cells depletion in mice results in a reduction of CCL4-induced liver fibrosis (Novobrantseva et al., 2005) and improves fat-induced inflammation (Sutti and Albano, 2020). Inversely, B cells accumulate in NASH livers and microbiota-driven activation of intrahepatic B cells aggravates NASH (Barrow et al., 2021). Immune-associated genes linked to antigen presentation have been reported in NASH gene signatures (Haas et al., 2019). Type 2 immunity is characterized by increased production of IL-4 and is directly involved in tissue repair and regeneration following injury. Many studies suggested a critical role for IL-4 activated macrophages in the resolution of inflammation and restoration of tissue homeostasis (Gieseck et al., 2018). Furthermore, associations between IL-4 polymorphisms and the risk of liver diseases have been reported (Wu et al., 2015).

Finally, as host genetics can contribute to variations in gut microbiome composition (Awany et al., 2018) and it is well-established that the gut microbiome contributes to NAFLD progression, we sought to determine whether gut microbiome changes were associated with any of the 86 AST- and ALT-associated variants in the CCHC, as genotype-microbiome associations may uncover potential mechanisms through which host genetics contribute to increased liver disease risk in this population. Eight variants, corresponding to 9.3% of all variants, were significantly associated with a shift in gut microbiome profiles. Interestingly, the CC genotype of rs2727324 (*SMARCD2*, *TCAM1P*) was associated with depletion of *Bacteroides dorei* and Marinifilaceae, which we previously reported as depleted in subjects with liver fibrosis in the CCHC (Kwan et al., 2021). In addition, we observed a trend towards increased Firmicutes/Bacteroidetes ratio for 5 of the 8 variants, reaching significance for rs62292950 (*DNAJC13*) and rs7041363 (*AKNA*). A change in Firmicutes/Bacteroidetes ratio has been often observed in obesity (Magne et al., 2020). An increased Firmicutes/Bacteroidetes ratio has also been associated with NAFLD (Monga Kravetz et al., 2020; Jasirwan et al., 2021; Lee et al., 2021).

Limitations to this study are the small sample size, and potential false positive associations inherent in association studies. The reported results should therefore be interpreted with caution. Another limitation is the use of FibroScan measurements to assess presence of liver steatosis and liver fibrosis. While liver biopsy remains the gold standard for diagnosis and staging of liver steatosis and fibrosis, the non-invasive nature of FibroScan allows for detection of liver disease across larger populations. Nevertheless, there is a lack of consensus across different studies on the optimal cutoffs for FibroScan CAP and LSM values to accurately diagnose liver steatosis and fibrosis respectively.

In conclusion, among variants identified from a large meta-analysis of United Kingdom and Japanese cohorts, we validated the association of 86 variants with elevated AST and/or ALT levels in a third ethnic population: Mexican Americans, suggesting that these associations are robust across ancestries. Some of these variants were further associated with steatosis, liver fibrosis and/or gut microbiome changes. Several variants had stronger associations than the well-known *PNPLA3* variant and a panel of four variants was strongly associated with risk for advanced liver fibrosis. Remarkably, the predicted effect of ALT-associated variants on canonical pathways was distinct from the predicted effect of AST-associated variants, and suggested dysregulation of immune-related processes, particularly B cell functions. These newly identified variants and related canonical pathways may have utility in risk modeling and disease prevention in this high-risk population.

## Data Availability

The original contributions presented in the study are included in the article/Supplementary Materials, further inquiries can be directed to the corresponding author.

## Abbreviations

CCHC, Cameron County Hispanic Cohort; PNPLA3, phospholipase domain-containing protein 3; MYCT1, Myc target in myeloid cells 1; APOH, apoliproprotein H; S1PR2, sphingosine-1-phosphate receptor 2; RGL1, ral guanine nucleotide dissociation stimulator-like 1; AOR, adjusted odds ratio; CI, confidence interval; DNAJC13, DNAJ/HSP40 homolog, subfamily C, member 13; TM6SF2, transmembrane 6 superfamily, member 2; CAP, controlled attenuation parameter; VCTE, vibration-controlled transient elastography; UKBB, United Kingdom Biobank; BBJ, BioBank Japan; EAF, effect allele frequency; PRKD2, protein kinase D2; STRN4, striatin, calmodulin-binding protein 4
