# Supplementary material for: Genetic variants associated with circulating liver injury markers in Mexican Americans, a population at risk for non-alcoholic fatty liver disease
Source: Front Genet. 2022 Oct 26;13:995488. doi: 10.3389/fgene.2022.995488 (PMC9644071; doi:10.3389/fgene.2022.995488)
Supplement: Supplementary file 2 [file DataSheet2.DOCX]

**Supplementary Table S2.** Resources used for stool DNA extraction, 16S rRNA amplicon sequencing and bioinformatic analysis.

| **Reagents** | **Source** |
| --- | --- |
| OMNIgene® GUT stool collection kit | DNA Genotek |
| QIAamp Fast DNA stool mini Kit | Qiagen |
| **Oligonucleotides for PCR** | **Sequence (5’-3’) ^a^** |
| Forward primer: V4 region of 16S rRNA gene | AATGATACGGCGACCACCGAGATCTACACGCTXXXXXXXXXXXXTATGGTAATTGTGTGYCAGCMGCCGCGGTAA |
| Reverse primer: V4 region of 16S rRNA gene | CAAGCAGAAGACGGCATACGAGATAGTCAGCCAGCCGGACTACNVGGGTWTCTAAT |
| **Oligonucleotides for sequencing** | **Sequence (5’-3’)** |
| Read1 primer | TATGGTAATTGTGTGYCAGCMGCCGCGGTAA |
| Read2 primer | AGTCAGCCAGCCGGACTACNVGGGTWTCTAAT |
| Index primer | AATGATACGGCGACCACCGAGATCTACACGCT |
| **Software and algorithms** | **Source** |
| QIIME | <http://qiime.org/> |
| VSEARCH v7 | <https://github.com/torognes/vsearch> |
| “cluster_otus” command | <https://drive5.com/usearch/manual/cmd_cluster_otus.html> |
| “unoise3” command | <https://drive5.com/usearch/manual/cmd_unoise3.html> |
| Mothur | <https://mothur.org/> |

^a^ XXXXXXXXXXXX is an index sequence for multiplexing libraries.

**Supplementary Table S3.** Frequency of effect alleles in CCHC.

| **rs ID** | **Chr** | **Position (GRCh38)** | **Ref** | **Alt** | **Gene annotation** | **Effect allele** | **EAF CCHC** | **EAF UKBB** | **EAF BBJ** |
| --- | --- | --- | --- | --- | --- | --- | --- | --- | --- |
| **rs2126259** | 8 | 9327636 | T | C | LOC157273 (i) | T | 32% | 10% | 1% |
| **rs11887534** | 2 | 43839108 | G | C | ABCG8 (e) | C | 10% | 7% | 1% |
| **rs2800290** | 9 | 100569123 | T | C | MSANTD3-TMEFF1, TMEFF1 (i) | C | 52% | 42% | 7% |
| **rs12824533** | 12 | 11638751 | A | G | LINC01252, ETV6 (inter) | G | 54% | 40% | 9% |
| **rs61352607** | 12 | 57445390 | G | T | INHBC (i) | T | 42% | 24% | 7% |
| **rs73545546** | 8 | 22059711 | T | C | DMTN (i) | C | 27% | 16% | 5% |
| **rs17743415** | 2 | 71335373 | T | C | ZNF638 (i) | C | 63% | 57% | 18% |
| **rs1800759** | 4 | 99144358 | T | G | LOC100507053 (i) | T | 56% | 39% | 16% |
| **rs76722284** | 5 | 75054108 | G | A | LINC01336, ANKRD31 (inter) | A | 24% | 4% | 10% |
| **rs62544382** | 9 | 33109998 | G | T | B4GALT1 (d) | T | 72% | 57% | 31% |
| **rs11601507** | 11 | 5679844 | C | A | TRIM5 (e) | A | 12% | 7% | 5% |
| **rs1024794** | 2 | 102130853 | A | G | IL1R1 (i) | G | 43% | 35% | 21% |
| **rs113895159** | 8 | 58553547 | T | C | SDCBP (i) | T | 75% | 67% | 37% |
| **rs17710008** | 6 | 152721900 | G | A | MYCT1 (e) | A | 23% | 19% | 12% |
| **rs75182702** | 3 | 46130331 | C | A | XCR1, CCR1 (inter) | A | 9% | 6% | 5% |
| **rs7209484** | 17 | 48065680 | T | C | NFE2L1, CBX1 (inter) | C | 40% | 24% | 22% |
| **rs2594973** | 3 | 11354347 | C | G | ATG7 (i) | C | 59% | 37% | 37% |
| **rs28383223** | 6 | 32615700 | C | T | HLA-DRB1, HLA-DQA1 (inter) | T | 65% | 58% | 46% |
| **rs6834314** | 4 | 87292656 | A | G | KLHL8, MIR5705 (inter) | A | 89% | 72% | 66% |
| **rs7776054** | 6 | 135097778 | A | G | HBS1L, MYB (inter) | A | 87% | 74% | 65% |
| **rs1200503** | 5 | 73055235 | C | G | FCHO2 (i) | C | 61% | 53% | 46% |
| **rs2954021** | 8 | 125469835 | A | G | TRIB1, LINC00861 (inter) | A | 58% | 49% | 45% |
| **rs68082079** | 9 | 109315250 | T | C | EPB41L4B (i) | T | 83% | 71% | 65% |
| **rs4638642** | 17 | 31312307 | G | C | EVI2B, NF1 (i) | G | 63% | 39% | 50% |
| **rs8067056** | 17 | 46006582 | T | C | MAPT (i) | T | 72% | 62% | 58% |
| **rs2954027** | 8 | 125473052 | T | A | TRIB1, LINC00861 (inter) | T | 63% | 53% | 51% |
| **rs11061602** | 12 | 122131417 | G | T | MLXIP (i) | T | 57% | 52% | 47% |
| **rs412662** | 21 | 29349061 | T | C | BACH1, BACH1-IT2 (inter) | C | 53% | 48% | 44% |
| **rs4074793** | 5 | 52897294 | A | G | ITGA1 (i) | G | 9% | 8% | 7% |
| **rs2950388** | 12 | 56639569 | C | T | ATP5B (i) | C | 79% | 72% | 67% |
| **rs2137537** | 12 | 70719307 | T | C | PTPRR (i) | T | 54% | 45% | 47% |
| **rs738409** | 22 | 43928847 | C | G | PNPLA3 (e) | G | 52% | 22% | 45% |
| **rs1365298** | 1 | 93392732 | G | A | DR1, FNBP1L (inter) | A | 41% | 18% | 36% |
| **rs193759** | 16 | 11186056 | G | A | CLEC16A, SOCS1 (inter) | A | 80% | 67% | 71% |
| **rs4835265** | 4 | 145900258 | C | A | ZNF827 (i) | A | 41% | 16% | 36% |
| **rs4135240** | 6 | 36679903 | T | C | CDKN1A (i) | T | 77% | 67% | 69% |
| **rs3176334** | 6 | 36680587 | T | C | CDKN1A (i) | T | 77% | 68% | 70% |
| **rs7018885** | 9 | 93130444 | T | C | NINJ1 (i) | T | 86% | 75% | 78% |
| rs7840329 | 8 | 80890620 | G | A | ZNF704, PAG1 (inter) | A | 57% | 36% | 52% |
| rs4711750 | 6 | 43789345 | T | A | VEGFA, LINC01512 (inter) | A | 60% | 50% | 55% |
| rs6762589 | 3 | 125208361 | G | A | SLC12A8 (i) | A | 56% | 41% | 52% |
| rs998584 | 6 | 43790159 | C | A | VEGFA, LINC01512 (inter) | A | 59% | 48% | 55% |

| rs11170319 | 12 | 52891302 | T | G | KRT78, KRT8 (inter) | G | 69% | 55% | 64% |
| --- | --- | --- | --- | --- | --- | --- | --- | --- | --- |
| rs876435 | 8 | 23016020 | G | A | RHOBTB2 (i) | G | 55% | 41% | 51% |
| rs4531558 | 12 | 52901297 | T | G | KRT8 (i) | G | 68% | 51% | 64% |
| rs6034011 | 20 | 14691531 | T | C | MACROD2 (i) | C | 51% | 32% | 49% |
| rs10007975 | 4 | 99527667 | C | T | C4orf17 (i) | C | 83% | 75% | 80% |
| rs964184 | 11 | 116778201 | G | C | ZPR1 (UTR) | G | 29% | 13% | 28% |
| rs656933 | 12 | 120878449 | A | G | SPPL3 (i) | A | 81% | 58% | 80% |
| rs2911980 | 8 | 6719619 | G | T | AGPAT5 (i) | T | 84% | 63% | 83% |
| rs9637973 | 6 | 54038354 | G | A | MLIP (i) | G | 55% | 31% | 54% |
| rs1815544 | 1 | 150507425 | C | T | TARS2 (UTR) | C | 73% | 60% | 72% |
| rs12544992 | 8 | 8804171 | C | G | MFHAS1 (i) | G | 73% | 47% | 72% |
| rs1664781 | 5 | 53980471 | G | A | ARL15 (i) | A | 85% | 69% | 84% |
| rs75004998 | 14 | 77051443 | G | A | LINC02288 (i) | G | 72% | 66% | 50% |
| rs1002436 | 1 | 88681169 | A | G | PKN2-AS1 (i) | G | 61% | 56% | 55% |
| rs292585 | 7 | 135199435 | C | T | WDR91 (i) | T | 80% | 73% | 61% |
| rs429358 | 19 | 44908684 | T | C | APOE (e) | T | 91% | 84% | 90% |
| rs4773169 | 13 | 110367115 | A | C | COL4A2 (i) | A | 65% | 60% | 62% |
| rs2376584 | 17 | 78406035 | G | A | PGS1 (i) | A | 44% | 41% | 10% |
| rs11159247 | 14 | 77043529 | G | A | LINC02288 (i) | G | 74% | 69% | 50% |
| rs7041363 | 9 | 114383763 | C | G | AKNA (i) | C | 55% | 51% | 49% |
| rs292580 | 7 | 135197671 | T | A | WDR91 (i) | A | 78% | 73% | 61% |
| rs140822617 | 3 | 172541716 | C | A | TNFSF10, LINC02068 (inter) | C | 93% | 87% | 87% |
| rs2282043 | 9 | 95450326 | C | T | PTCH1 (i) | C | 97% | 91% | 91% |
| rs4979373 | 9 | 114382224 | C | T | AKNA (i) | C | 54% | 51% | 49% |
| rs500802 | 13 | 21776998 | T | C | FGF9, LINC00424 (inter) | T | 72% | 68% | 60% |
| rs7029757 | 9 | 129804387 | G | A | TOR1B (i) | G | 95% | 90% | 82% |
| rs2519093 | 9 | 133266456 | T | C | ABO (i) | C | 86% | 81% | 72% |
| rs1658972 | 9 | 6665010 | C | T | GLDC, KDM4C (inter) | C | 91% | 86% | 89% |
| rs13074711 | 3 | 172550013 | T | C | TNFSF10, LINC02068 (inter) | T | 94% | 89% | 88% |
| rs3814420 | 4 | 183283075 | T | C | WWC2 (i) | T | 99% | 94% | 96% |
| rs2910953 | 5 | 39533216 | T | A | LINC02104, LINC00603 (inter) | A | 26% | 25% | 8% |
| rs7356034 | 3 | 171014810 | G | A | SLC2A2 (i) | A | 29% | 28% | 20% |
| rs864899 | 12 | 50827344 | A | G | ATF1, TMPRSS12 (inter) | G | 60% | 58% | 33% |
| rs351978 | 19 | 806256 | A | G | PTBP1 (i) | G | 60% | 58% | 45% |
| rs112771035 | 11 | 126355981 | C | G | ST3GAL4 (UTR) | C | 96% | 93% | 85% |
| rs17046767 | 2 | 113999632 | G | C | LINC01191 (i) | G | 95% | 92% | 83% |
| rs6700241 | 1 | 161531185 | A | G | HSPA6, FCGR3A (inter) | A | 82% | 80% | 73% |
| rs10849448 | 12 | 6384185 | A | G | LTBR (UTR) | A | 26% | 25% | 20% |
| rs2289850 | 5 | 157326276 | T | C | CYFIP2 (i) | T | 97% | 95% | 79% |
| rs1541098 | 1 | 94202414 | T | C | ARHGAP29 (i) | T | 77% | 76% | 64% |
| rs12617864 | 2 | 112957399 | T | G | IL37, IL36G (inter) | G | 46% | 25% | 46% |
| rs2642438 | 1 | 220796686 | A | G | MARC1 (e) | G | 83% | 70% | 83% |
| rs2841532 | 6 | 13512277 | G | A | GFOD1, SIRT5 (inter) | A | 76% | 69% | 76% |
| rs11643959 | 16 | 58736993 | T | G | GOT2, APOOP5 (inter) | T | 97% | 91% | 99% |
| rs12889639 | 14 | 103393869 | G | A | MARK3 (i) | A | 76% | 65% | 78% |
| rs2127015 | 15 | 73692882 | T | C | CD276 (i) | C | 54% | 51% | 55% |

| rs6879279 | 5 | 107543091 | G | C | EFNA5 (i) | G | 90% | 85% | 93% |
| --- | --- | --- | --- | --- | --- | --- | --- | --- | --- |
| rs6678642 | 1 | 59214574 | T | G | HSD52, FGGY (inter) | T | 82% | 66% | 85% |
| rs645040 | 3 | 136207780 | G | T | MSL2, PCCB (inter) | T | 79% | 77% | 82% |
| rs8041181 | 15 | 73689995 | C | T | CD276 (i) | T | 46% | 43% | 48% |
| rs4925546 | 1 | 247439666 | A | G | NLRP3 (i) | A | 39% | 37% | 41% |
| rs10863565 | 1 | 220801203 | T | C | MARC1 (i) | C | 86% | 76% | 91% |
| rs117143374 | 21 | 39183635 | T | C | PSMG1 (u) | T | 92% | 86% | 98% |
| rs6689009 | 1 | 196729333 | A | G | CFH (i) | A | 89% | 81% | 95% |
| rs1051713 | 10 | 45443298 | C | T | ALOX5 (i) | C | 88% | 83% | 94% |
| rs132642 | 22 | 36149089 | A | T | APOL3 (UTR) | T | 93% | 83% | 99% |
| rs10075805 | 5 | 31021251 | A | G | LOC105374704, CDH6 (inter) | A | 81% | 72% | 87% |
| rs3850625 | 1 | 201047168 | G | A | CACNA1S (e) | G | 90% | 88% | 97% |
| rs72823014 | 10 | 114026477 | G | A | NHLRC2, ADRB1 (inter) | G | 92% | 87% | 100% |
| rs3731714 | 2 | 201196097 | C | T | CASP10 (i) | C | 77% | 68% | 84% |
| rs134489 | 22 | 28333779 | A | T | TTC28 (i) | A | 75% | 73% | 82% |
| rs2241883 | 2 | 88124547 | T | C | FABP1 (e) | T | 70% | 69% | 77% |
| rs30386 | 5 | 179863845 | T | G | TBC1D9B (e) | G | 72% | 44% | 80% |
| rs11704551 | 22 | 29381515 | C | T | AP1B1 (i) | C | 86% | 74% | 96% |
| rs2943654 | 2 | 226248038 | C | T | LOC646736, MIR5702 (inter) | T | 81% | 65% | 91% |
| rs13108218 | 4 | 3442204 | A | G | HGFAC (i) | A | 48% | 38% | 55% |
| rs12129745 | 1 | 28245806 | G | C | ATPIF1, SESN2 (inter) | G | 79% | 68% | 90% |
| rs11088963 | 21 | 44789511 | C | G | UBE2G2 (i) | C | 72% | 51% | 82% |
| rs4880197 | 9 | 137073348 | G | A | SAPCD2, UAP1L1 (inter) | A | 26% | 25% | 30% |
| rs705699 | 12 | 55991020 | G | A | RAB5B (i) | G | 68% | 58% | 79% |
| rs1277930 | 1 | 109279521 | G | A | PSRC1 (d) | A | 79% | 77% | 92% |
| rs12979658 | 19 | 7827201 | A | G | CLEC4GP1, EVI5L (inter) | G | 30% | 28% | 35% |
| rs6712203 | 2 | 164700808 | C | T | COBLL1 (i) | C | 78% | 63% | 91% |
| rs13275089 | 8 | 102650624 | T | C | KLF10 (i) | T | 72% | 63% | 85% |
| rs900400 | 3 | 157080986 | T | C | LINC02029, LINC00880 (inter) | C | 41% | 40% | 49% |
| rs10842708 | 12 | 26321934 | G | A | SSPN, ITPR2 (inter) | G | 61% | 24% | 74% |
| rs4484649 | 8 | 10713981 | C | A | C8orf74, SOX7 (inter) | C | 59% | 40% | 71% |
| rs6938097 | 6 | 21939097 | G | A | CASC15 (i) | G | 82% | 72% | 99% |
| rs74367983 | 16 | 54381615 | G | A | LINC02140, LOC101927480 (inter) | A | 12% | 4% | 14% |
| rs6440123 | 3 | 142940519 | A | G | LOC100507389 (i) | A | 77% | 65% | 94% |
| rs3739235 | 8 | 141219046 | A | G | SLC45A4 (i) | G | 68% | 60% | 85% |
| rs61980636 | 14 | 94318281 | C | T | SERPINA6 (i) | T | 36% | 20% | 46% |
| rs2862954 | 10 | 100152307 | T | C | ERLIN1 (e) | T | 75% | 50% | 96% |
| rs4841436 | 8 | 10736964 | C | A | LOC102723313 (i) | C | 54% | 40% | 69% |
| rs8041057 | 15 | 40038428 | C | T | SRP14 (i) | C | 54% | 29% | 70% |
| rs7740188 | 6 | 130024960 | G | A | L3MBTL3 (i) | A | 73% | 69% | 96% |
| rs10409243 | 19 | 10222312 | C | T | S1PR2 (UTR) | C | 57% | 40% | 75% |
| rs2727324 | 17 | 63844742 | G | C | SMARCD2, TCAM1P (inter) | C | 41% | 33% | 55% |
| rs9876116 | 3 | 37042249 | A | G | MLH1 (i) | A | 63% | 54% | 85% |
| rs11240351 | 1 | 205075211 | A | G | CNTN2 (UTR) | G | 62% | 40% | 86% |
| rs339969 | 15 | 60591082 | C | A | RORA-AS1 (i) | A | 65% | 61% | 91% |
| rs6734238 | 2 | 113083453 | A | G | IL1F10, IL1RN (inter) | A | 68% | 60% | 95% |

| rs7256564 | 19 | 33398687 | A | G | PEPD (i) | A | 37% | 31% | 53% |
| --- | --- | --- | --- | --- | --- | --- | --- | --- | --- |
| rs1545536 | 8 | 143560999 | C | T | GSDMD (i) | T | 30% | 22% | 43% |
| rs57457691 | 19 | 33420799 | T | C | PEPD (i) | T | 38% | 37% | 54% |
| rs61394864 | 15 | 84626943 | T | C | ZSCAN2, SCAND2P (inter) | C | 58% | 54% | 85% |
| rs2235698 | 6 | 29588001 | A | G | OR2H2 (e) | G | 27% | 23% | 40% |
| rs3798232 | 6 | 115996281 | T | C | FRK (i) | C | 44% | 40% | 68% |
| rs9616810 | 22 | 50656498 | C | T | ARSA, SHANK3 (inter) | T | 31% | 22% | 48% |
| rs3756772 | 6 | 116003979 | C | T | FRK (e) | T | 45% | 40% | 70% |
| rs6747874 | 2 | 100962027 | G | A | NPAS2 (i) | A | 43% | 22% | 67% |
| rs13197551 | 6 | 35316181 | T | C | DEF6 (i) | T | 24% | 20% | 39% |
| rs6938946 | 6 | 35312920 | C | T | DEF6 (i) | C | 19% | 16% | 33% |
| rs1970619 | 13 | 27865252 | T | C | PLUT (i) | T | 41% | 40% | 82% |
| rs11557154 | 9 | 34107507 | C | T | DCAF12 (e) | T | 27% | 13% | 56% |
| rs445 | 7 | 92779056 | C | T | CDK6 (i) | T | 14% | 9% | 31% |
| rs12154248 | 7 | 28152756 | C | T | JAZF1 (i) | T | 21% | 8% | 49% |
| rs1938500 | 1 | 65675819 | G | T | LEPR, PDE4B (inter) | T | 31% | 19% | 76% |
| rs10145626 | 14 | 93998863 | G | A | C14orf48 (i) | A | 21% | 5% | 59% |
| rs72623176 | 2 | 168977860 | G | A | ABCB11 (i) | A | 9% | 4% | 33% |
| rs117743490 | 17 | 17280660 | A | C | COPS3 (UTR) | C | 4% | 3% | 17% |
| rs2277998 | 19 | 7766742 | G | A | CLEC4M (e) | G | 70% | 70% | 85% |
| rs7155922 | 14 | 54771591 | G | A | SAMD4A (i) | G | 42% | 42% | 42% |
| rs216141 | 5 | 150064431 | C | T | CSF1R (i) | C | 28% | 28% | 52% |
| rs10787429 | 10 | 112189906 | T | C | GPAM, TECTB (inter) | T | 27% | 27% | 23% |
| rs10887777 | 10 | 88047609 | T | C | PTEN, RNLS (inter) | C | 26% | 26% | 61% |
| rs7700107 | 4 | 17878793 | A | C | LCORL (i) | C | 14% | 14% | 24% |
| rs12320328 | 12 | 25255530 | A | G | KRAS, LMNTD1 (inter) | A | 92% | 92% | 97% |
| rs3185057 | 17 | 80390047 | G | A | RNF213 (e) | G | 92% | 92% | 93% |
| rs17138478 | 17 | 37713312 | C | A | HNF1B (i) | C | 87% | 87% | 79% |
| rs58504358 | 8 | 133543494 | T | C | ST3GAL1 (i) | T | 81% | 81% | 75% |
| rs5752776 | 22 | 28712241 | A | G | CHEK2 (i) | G | 67% | 67% | 88% |
| rs12454712 | 18 | 63178651 | T | C | BCL2 (i) | T | 62% | 62% | 48% |
| rs12799680 | 11 | 95450772 | T | C | LOC100129203, FAM76B (inter) | T | 90% | 91% | 85% |
| rs10858828 | 12 | 89147879 | G | C | LINC02458, DUSP6 (inter) | G | 77% | 78% | 57% |
| rs17855739 | 19 | 5831829 | C | T | FUT6 (e) | C | 95% | 96% | 94% |
| rs421491 | 13 | 110647653 | T | C | CARS2 (i) | T | 94% | 95% | 80% |
| rs13212562 | 6 | 27332531 | A | G | VN1R10P, ZNF204P (inter) | A | 87% | 88% | 92% |
| rs6973917 | 7 | 56022948 | T | A | PSPH (i) | T | 25% | 25% | 37% |
| rs78900599 | 3 | 149470375 | A | G | TM4SF1-AS1, TM4SF4 (inter) | A | 91% | 93% | 90% |
| rs17803745 | 20 | 39913927 | C | T | LINC01734, LINC01370 (inter) | C | 65% | 66% | 70% |
| rs1468615 | 7 | 87445648 | T | C | ABCB4 (i) | T | 80% | 82% | 72% |
| rs11714574 | 3 | 58394738 | A | T | PXK (i) | A | 58% | 59% | 24% |
| rs11698868 | 20 | 34962143 | C | T | MYH7B (i) | T | 19% | 19% | 17% |
| rs7599 | 19 | 35547488 | A | G | TMEM147 (UTR) | A | 36% | 37% | 24% |
| rs12489967 | 3 | 170355551 | A | C | PRKCI, SKIL (inter) | C | 82% | 84% | 89% |
| rs34467936 | 11 | 47893747 | A | G | NUP160, PTPRJ (inter) | A | 62% | 64% | 70% |
| rs17119056 | 5 | 140695889 | C | T | HARS2 (i) | T | 9% | 9% | 6% |

| rs503581 | 18 | 57461443 | G | A | ONECUT2 (i) | G | 87% | 90% | 93% |
| --- | --- | --- | --- | --- | --- | --- | --- | --- | --- |
| rs12143966 | 1 | 247438055 | G | A | NLRP3 (i) | G | 57% | 59% | 52% |
| rs7488780 | 12 | 20426458 | G | C | PDE3A (i) | G | 77% | 80% | 95% |
| rs7246479 | 19 | 55312964 | T | G | TMEM150B (e) | G | 49% | 51% | 19% |
| rs2053158 | 17 | 75397378 | T | A | GRB2 (i) | A | 81% | 84% | 95% |
| rs17145884 | 11 | 62432704 | C | T | AHNAK (d) | C | 79% | 82% | 71% |
| rs40270 | 5 | 56508725 | A | C | LINC01948, C5orf67 (inter) | C | 74% | 77% | 15% |
| rs2110944 | 2 | 36863090 | T | C | STRN (i) | C | 51% | 53% | 38% |
| rs5751775 | 22 | 23924539 | T | C | MIF-AS1, GSTT2B (inter) | T | 39% | 41% | 54% |
| rs4821764 | 22 | 38203357 | G | A | MAFF (i) | A | 55% | 58% | 66% |
| rs10931283 | 2 | 187346716 | C | T | CALCRL (i) | T | 61% | 65% | 76% |
| rs2239222 | 14 | 72545177 | A | G | RGS6 (i) | A | 61% | 65% | 58% |
| rs636202 | 6 | 139522446 | T | C | LINC01625, LOC100132735 (inter) | T | 45% | 48% | 69% |
| rs62292950 | 3 | 132479151 | T | G | DNAJC13 (i) | T | 82% | 87% | 87% |
| rs31672 | 7 | 87430383 | C | T | ABCB4 (i) | T | 74% | 79% | 69% |
| rs11054848 | 12 | 12362395 | A | G | BORCS5 (i) | G | 59% | 63% | 37% |
| rs79188145 | 10 | 73021160 | A | G | P4HA1 (i) | A | 88% | 94% | 68% |
| rs11878604 | 19 | 40827379 | T | C | CYP2T1P, CYP2A6 (inter) | T | 87% | 93% | 61% |
| rs7117339 | 11 | 94137172 | C | T | PANX1 (i) | C | 82% | 88% | 95% |
| rs4485425 | 17 | 75771356 | A | G | GALK1, H3F3B (inter) | G | 67% | 72% | 53% |
| rs7976853 | 12 | 3231491 | C | T | TSPAN9 (i) | T | 9% | 10% | 6% |
| rs56175344 | 11 | 94131227 | C | G | PANX1 (i) | C | 82% | 88% | 96% |
| rs17236494 | 3 | 160455812 | A | C | TRIM59, KPNA4 (inter) | C | 25% | 27% | 16% |
| rs6916318 | 6 | 127113961 | A | T | MIR588, RSPO3 (inter) | T | 49% | 53% | 49% |
| rs686548 | 20 | 12992873 | A | T | LINC01722, SPTLC3 (inter) | A | 36% | 39% | 41% |
| rs73382439 | 6 | 20404189 | T | C | E2F3 (i) | C | 76% | 83% | 60% |
| rs481206 | 11 | 72786417 | C | T | STARD10 (i) | T | 31% | 34% | 30% |
| rs6589941 | 11 | 122648314 | T | G | MIR100HG, UBASH3B (inter) | G | 36% | 39% | 39% |
| rs11204725 | 1 | 150770224 | T | C | CTSS, CTSK (inter) | C | 50% | 55% | 45% |
| rs6602909 | 13 | 113849020 | T | C | GAS6 (i) | C | 30% | 33% | 29% |
| rs3786876 | 19 | 38268463 | T | C | SPINT2 (i) | C | 14% | 15% | 15% |
| rs12500824 | 4 | 76495474 | A | G | SHROOM3 (i) | A | 32% | 35% | 48% |
| rs4766568 | 12 | 111286895 | T | C | CUX2 (i) | T | 77% | 86% | 35% |
| rs6755970 | 2 | 111932403 | A | G | MERTK (i) | A | 34% | 38% | 76% |
| rs1052238 | 1 | 198665496 | T | C | PTPRC (i) | T | 42% | 47% | 29% |
| rs196210 | 10 | 119711739 | C | G | BAG3, INPP5F (inter) | G | 37% | 41% | 31% |
| rs8023703 | 15 | 72211718 | G | A | PKM (i) | A | 68% | 77% | 66% |
| rs6547692 | 2 | 27512105 | G | A | GCKR (i) | G | 39% | 44% | 56% |
| rs6541998 | 2 | 112027660 | T | C | MERTK (i) | T | 34% | 39% | 76% |
| rs223454 | 4 | 102789773 | G | A | LOC102723704 (i) | G | 45% | 52% | 54% |
| rs10148309 | 14 | 50734993 | C | T | NIN (i) | C | 74% | 85% | 45% |
| rs10134101 | 14 | 50390914 | A | G | CDKL1 (i) | A | 83% | 96% | 97% |
| rs10141892 | 14 | 34715117 | T | C | CFL2 (u) | C | 51% | 59% | 74% |
| rs1800978 | 9 | 104903697 | C | G | ABCA1 (UTR) | C | 76% | 88% | 79% |
| rs1917368 | 7 | 17872129 | G | T | SNX13 (i) | G | 53% | 61% | 57% |
| rs4940576 | 18 | 63181406 | T | C | BCL2 (i) | C | 66% | 77% | 53% |

| rs717118 | 17 | 70468676 | T | A | KCNJ2, CASC17 (inter) | T | 41% | 48% | 29% |
| --- | --- | --- | --- | --- | --- | --- | --- | --- | --- |
| rs28650012 | 16 | 80463444 | G | C | LOC102724084 (i) | G | 23% | 27% | 38% |
| rs7189522 | 16 | 80464197 | T | C | LOC102724084 (i) | T | 23% | 27% | 38% |
| rs2289125 | 11 | 89491285 | A | C | NOX4 (UTR) | C | 67% | 79% | 51% |
| rs4485410 | 17 | 70379805 | T | C | KCNJ2, CASC17 (inter) | T | 66% | 78% | 59% |
| rs6591673 | 11 | 61930432 | G | A | RAB3IL1, BEST1 (inter) | G | 57% | 67% | 69% |
| rs8027647 | 15 | 72282873 | A | T | PARP6, CELF6 (inter) | T | 62% | 73% | 32% |
| rs11706136 | 3 | 33056563 | G | A | GLB1 (i) | A | 58% | 69% | 45% |
| rs508196 | 10 | 17974944 | C | T | SLC39A12 (i) | C | 47% | 56% | 45% |
| rs671275 | 8 | 60613221 | G | A | RAB2A (i) | A | 20% | 24% | 30% |
| rs869202 | 11 | 78416868 | A | G | GAB2 (i) | A | 69% | 83% | 54% |
| rs12406530 | 1 | 112574591 | A | G | ST7L (i) | A | 66% | 79% | 50% |
| rs7243073 | 18 | 58463061 | A | C | MIR122, ALPK2 (inter) | A | 22% | 26% | 24% |
| rs2491441 | 1 | 183833125 | T | C | RGL1 (i) | C | 17% | 20% | 31% |
| rs9959832 | 18 | 58419588 | C | T | NEDD4L, MIR122 (inter) | C | 17% | 20% | 18% |
| rs11002301 | 10 | 77814320 | G | A | DLG5 (i) | A | 28% | 34% | 23% |
| rs10832570 | 11 | 16227964 | A | G | SOX6 (i) | A | 50% | 61% | 51% |
| rs1401454 | 11 | 16228637 | C | T | SOX6 (i) | C | 50% | 61% | 50% |
| rs11770163 | 7 | 116777794 | G | C | MET (i) | C | 27% | 33% | 14% |
| rs4782568 | 16 | 83946924 | C | G | MLYCD, OSGIN1 (inter) | C | 45% | 55% | 24% |
| rs2291160 | 16 | 88631079 | G | A | ZC3H18 (i) | G | 45% | 55% | 54% |
| rs4135247 | 3 | 12355089 | G | A | PPARG (i) | G | 35% | 43% | 53% |
| rs1047891 | 2 | 210675783 | C | A | CPS1 (e) | A | 26% | 32% | 15% |
| rs313839 | 19 | 46718300 | C | G | PRKD2, STRN4 (inter) | G | 13% | 16% | 18% |
| rs2450128 | 11 | 78229029 | G | A | GAB2 (i) | G | 69% | 85% | 56% |
| rs3096168 | 5 | 96898508 | G | A | ERAP2 (i) | G | 41% | 50% | 54% |
| rs13145218 | 4 | 48041928 | G | A | NIPAL1, TXK (inter) | A | 17% | 21% | 27% |
| rs6441017 | 3 | 155844283 | C | T | SLC33A1 (i) | C | 59% | 73% | 71% |
| rs12799896 | 11 | 65408899 | C | T | FRMD8 (i) | C | 67% | 83% | 92% |
| rs13395911 | 2 | 232655544 | A | T | EFHD1 (i) | T | 48% | 60% | 32% |
| rs72816437 | 2 | 48047453 | A | T | FBXO11, FOXN2 (inter) | T | 31% | 39% | 5% |
| rs2394529 | 10 | 69225511 | G | C | LOC101928994 (i) | C | 56% | 70% | 18% |
| rs12413013 | 10 | 17219763 | G | A | VIM-AS1 (i) | G | 55% | 69% | 72% |
| rs10430531 | 10 | 77900367 | T | C | DLG5 (i) | C | 21% | 26% | 23% |
| rs333947 | 1 | 109928142 | G | A | CSF1 (i) | G | 67% | 85% | 71% |
| rs2468175 | 8 | 119175470 | A | G | COLEC10, MAL2 (inter) | G | 50% | 63% | 63% |
| rs2568207 | 2 | 85188306 | A | T | TCF7L1 (i) | T | 48% | 61% | 22% |
| rs28642812 | 12 | 123409363 | T | C | KMT5A (d) | C | 56% | 72% | 74% |
| rs380731 | 19 | 54255708 | C | T | LILRB5 (i) | T | 21% | 27% | 3% |
| rs3758526 | 10 | 94344908 | T | G | NOC3L (e) | G | 10% | 13% | 32% |
| rs11932940 | 4 | 23924364 | G | A | PPARGC1A (i) | A | 53% | 69% | 61% |
| rs11086005 | 19 | 15864286 | G | T | LINC01835 (i) | G | 48% | 62% | 19% |
| rs4766462 | 12 | 111433337 | A | T | SH2B3 (i) | A | 60% | 79% | 19% |
| rs1497406 | 1 | 16178825 | A | G | EPHA2, ARHGEF19 (inter) | G | 44% | 58% | 79% |
| rs11220136 | 11 | 125582185 | C | T | STT3A-AS1 (i) | T | 5% | 7% | 0% |
| rs217184 | 16 | 72072066 | T | C | HPR (i) | T | 61% | 81% | 77% |

| rs2467663 | 8 | 144729622 | T | C | ZNF251 (i) | T | 50% | 66% | 31% |
| --- | --- | --- | --- | --- | --- | --- | --- | --- | --- |
| rs10096191 | 8 | 71507263 | T | G | EYA1, MSC (inter) | G | 6% | 8% | 12% |
| rs2832277 | 21 | 29236564 | A | G | LINC00189 (i) | G | 66% | 88% | 61% |
| rs273507 | 19 | 18111154 | A | C | MAST3 (i) | C | 22% | 29% | 13% |
| rs36086195 | 1 | 16184399 | C | T | EPHA2, ARHGEF19 (inter) | T | 43% | 58% | 77% |
| rs6894249 | 5 | 132461855 | A | G | C5orf56 (i) | A | 45% | 61% | 38% |
| rs687621 | 9 | 133261662 | G | A | ABO (i) | G | 23% | 32% | 46% |
| rs56360131 | 16 | 86063872 | C | T | IRF8, LINC01082 (inter) | T | 14% | 19% | 10% |
| rs7097660 | 10 | 99052093 | C | G | HPSE2 (i) | G | 55% | 77% | 80% |
| rs12928392 | 16 | 58037060 | T | A | MMP15 (i) | T | 54% | 76% | 61% |
| rs12904 | 1 | 155134221 | G | A | EFNA1 (UTR) | G | 42% | 59% | 9% |
| rs142451335 | 1 | 2244114 | C | T | SKI (i) | T | 5% | 7% | 1% |
| rs8176279 | 17 | 43058379 | A | C | BRCA1 (i) | C | 24% | 34% | 35% |
| rs12876036 | 13 | 73550539 | G | A | KLF5, LINC00392 (inter) | G | 39% | 56% | 67% |
| rs45587331 | 10 | 96700824 | G | A | PIK3AP1 (i) | A | 13% | 19% | 30% |
| rs12806061 | 11 | 2987174 | G | A | NAP1L4 (i) | G | 47% | 70% | 33% |
| rs151401 | 4 | 102268971 | A | G | SLC39A8 (i) | G | 15% | 23% | 5% |
| rs7168849 | 15 | 89802996 | A | G | ANPEP (i) | A | 59% | 91% | 71% |
| rs9657643 | 9 | 130276442 | A | G | HMCN2 (i) | A | 18% | 27% | 15% |
| rs6088521 | 20 | 34545380 | A | C | DYNLRB1, MAP1LC3A (inter) | C | 31% | 48% | 26% |
| rs11657440 | 17 | 81513660 | T | C | ACTG1 (u) | C | 27% | 42% | 9% |
| rs11070841 | 15 | 51201770 | G | A | MIR4713HG (i) | A | 14% | 21% | 29% |
| rs439401 | 19 | 44911194 | T | C | APOE, APOC1 (inter) | C | 41% | 64% | 40% |
| rs11555039 | 11 | 1758975 | A | G | CTSD (e) | G | 6% | 9% | 1% |
| rs33204 | 5 | 77438259 | C | T | WDR41 (e) | C | 36% | 56% | 51% |
| rs11678685 | 2 | 168854097 | G | A | NOSTRIN (i) | A | 20% | 32% | 33% |
| rs1477066 | 17 | 72110287 | T | C | SOX9-AS1 (i) | C | 41% | 67% | 63% |
| rs7481951 | 11 | 22250324 | A | T | ANO5 (e) | T | 36% | 59% | 14% |
| rs35064159 | 6 | 95634240 | T | C | MANEA, FUT9 (inter) | C | 5% | 8% | 8% |
| rs2289702 | 15 | 78944951 | C | T | CTSH (e) | T | 6% | 10% | 7% |
| rs7953767 | 12 | 20904347 | C | T | SLCO1B3 (i) | T | 10% | 16% | 1% |
| rs6742283 | 2 | 135839297 | G | A | MCM6 (d) | G | 48% | 80% | 53% |
| rs5758487 | 22 | 41830391 | C | T | CCDC134, SREBF2 (inter) | C | 29% | 48% | 30% |
| rs1046271 | 19 | 3750624 | A | C | TJP3 (e) | A | 27% | 45% | 29% |
| rs2624817 | 3 | 50080087 | A | G | RBM6, RBM5 (inter) | A | 10% | 17% | 14% |
| rs56094641 | 16 | 53772541 | A | G | FTO (i) | G | 24% | 40% | 20% |
| rs9372475 | 6 | 117180825 | T | C | RFX6, VGLL2 (inter) | T | 22% | 37% | 28% |
| rs12593917 | 15 | 49544670 | G | T | FAM227B (i) | T | 22% | 38% | 15% |
| rs1077834 | 15 | 58431280 | T | C | LIPC (u) | T | 45% | 78% | 49% |
| rs2070895 | 15 | 58431740 | G | A | LIPC (u) | G | 45% | 78% | 49% |
| rs660173 | 1 | 111194561 | C | T | DENND2D (i) | C | 18% | 31% | 33% |
| rs1538742 | 1 | 219494910 | C | A | LOC102723886 (i) | A | 32% | 57% | 46% |
| rs4788460 | 16 | 72120610 | C | T | PMFBP1 (i) | T | 16% | 29% | 7% |
| rs1760940 | 14 | 20470092 | A | C | PNP (i) | C | 14% | 25% | 15% |
| rs11621792 | 14 | 24402720 | C | T | NYNRIN (i) | T | 24% | 45% | 6% |
| rs58542926 | 19 | 19268740 | C | T | TM6SF2 (e) | T | 4% | 8% | 8% |

| rs61856806 | 10 | 96706525 | T | A | PIK3AP1 (i) | A | 10% | 18% | 31% |
| --- | --- | --- | --- | --- | --- | --- | --- | --- | --- |
| rs6577596 | 3 | 17791337 | C | T | LOC105376975, LOC339862 (inter) | C | 21% | 40% | 1% |
| rs11882796 | 19 | 48722509 | A | T | RASIP1 (i) | T | 28% | 54% | 5% |
| rs35173225 | 7 | 73586691 | C | T | TBL2, MLXIPL (inter) | T | 6% | 12% | 10% |
| rs1008897 | 7 | 7233809 | G | A | C1GALT1 (i) | G | 16% | 37% | 6% |
| rs241597 | 20 | 3925431 | A | G | PANK2 (UTR) | G | 6% | 13% | 16% |
| rs71423566 | 2 | 178480456 | C | T | MIR548N (i) | T | 13% | 32% | 7% |
| rs117643180 | 17 | 7282460 | C | A | SLC2A4 (i) | A | 1% | 3% | 1% |
| rs1794898 | 12 | 121102728 | G | A | OASL, P2RX7 (inter) | G | 6% | 16% | 6% |
| rs1801690 | 17 | 66212167 | C | G | APOH (e) | G | 2% | 6% | 10% |
| rs4579782 | 1 | 26700496 | C | G | ARID1A (i) | G | 2% | 8% | 1% |

Ref: reference allele; Alt: alternate allele; annotations: (e) exonic, (i) intronic, (u) upstream, (d) downstream, (inter) intergenic, (UTR) untranslated region; EAF: effect allele frequency; CCHC: Cameron County Hispanic Cohort; UKBB: UK BioBank; BBJ: BioBank Japan. Annotations and EAF in UKBB and BBJ were extracted from (Chen et al, 2021). Rs ID in bold have 10% or greater increase in EAF in CCHC compared to UKBB and BBJ.

**Supplementary Table S4.** Association of 86 SNPs with elevated AST and ALT levels in CCHC.

|  |  | **CCHC** | | | | | **AST** | | | | **ALT** | | | |
| --- | --- | --- | --- | --- | --- | --- | --- | --- | --- | --- | --- | --- | --- | --- |
| **rsID** | **Gene annotation** | **EA** |  | **WT**  **(%)** | **Het**  **(%)** | **Hom**  **(%)** | **AOR [95% CI]**  **(2 vs 0-1 EA)** | **p** | **AOR [95% CI]**  **(1-2 vs 0 EA)** | **p** | **AOR [95% CI]**  **(2 vs 0-1 EA)** | **p** | **AOR [95% CI]**  **(1-2 vs 0 EA)** | **p** |
| rs2519093 | ABO | C | Alt | 2% | 26% | 73% |  |  |  |  |  |  | 8.05 [1.01-  64.36] | 0.049 |
| rs7041363 | AKNA | C | Ref | 30% | 47% | 23% | 1.57 [1.05-  2.34] | 0.028 |  |  |  |  |  |  |
| rs11704551 | AP1B1 | C | Ref | 73% | 25% | 2% | 1.79 [1.12-  2.86] | 0.015 |  |  | 1.52 [0.96-  2.38] | 0.050 |  |  |
| rs1801690 | APOH | G | Alt | 97% | 3% | 0% |  |  | 2.58 [0.97-  6.87] | 0.050 |  |  |  |  |
| rs1664781 | ARL15 | A | Alt | 3% | 25% | 72% |  |  |  |  | 1.52 [1.01-  2.28] | 0.044 |  |  |
| rs9616810 | ARSA, SHANK3 | T | Alt | 47% | 43% | 9% |  |  | 1.97 [1.38-  2.80] | <0.001 |  |  |  |  |
| rs2950388 | ATP5B | C | Ref | 60% | 36% | 4% | 1.54 [1.03-  2.33] | 0.034 |  |  | 1.75 [1.22-  2.56] | 0.003 |  |  |
| rs12129745 | ATPIF1, SESN2 | G | Ref | 62% | 33% | 5% | 1.47 [0.99-  2.22] | 0.050 |  |  | 1.69 [1.11-  2.56] | 0.013 |  |  |
| rs196210 | BAG3, INPP5F | G | Alt | 41% | 47% | 13% | 1.84 [1.11-  3.05] | 0.018 |  |  |  |  |  |  |
| rs11054848 | BORCS5 | G | Alt | 17% | 48% | 35% |  |  | 1.85 [1.05-  3.25] | 0.032 |  |  |  |  |
| rs4484649 | C8orf74, SOX7 | C | Ref | 35% | 47% | 18% |  |  |  |  | 1.43 [0.99-  2.08] | 0.050 |  |  |
| rs10931283 | CALCRL | T | Alt | 17% | 46% | 37% |  |  | 1.95 [1.18-  3.23] | 0.009 |  |  | 1.93 [1.08-  3.46] | 0.027 |
| rs421491 | CARS2 | T | Ref | 88% | 11% | 1% | 2.63 [1.25-  5.56] | 0.010 |  |  |  |  |  |  |
| rs12979658 | CLEC4GP1, EVI5L | G | Alt | 49% | 42% | 9% | 1.84 [1.01-  3.33] | 0.046 |  |  |  |  |  |  |
| rs11240351 | CNTN2 | G | Alt | 15% | 46% | 39% | 1.57 [1.07-  2.31] | 0.020 | 1.94 [1.05-  3.56] | 0.033 | 1.54 [1.08-  2.21] | 0.018 |  |  |
| rs4766568 | CUX2 | T | Ref | 59% | 36% | 5% | 1.64 [1.10-  2.44] | 0.015 |  |  |  |  |  |  |
| rs62292950 | DNAJC13 | T | Ref | 67% | 29% | 4% |  |  | 3.85 [1.11-  12.5] | 0.033 |  |  |  |  |
| rs6088521 | DYNLRB1, MAP1LC3A | C | Alt | 47% | 42% | 10% | 1.70 [0.96-  3.01] | 0.050 |  |  |  |  |  |  |
| rs4638642 | EVI2B, NF1 | G | Ref | 39% | 47% | 13% |  |  |  |  |  |  | 2.18 [1.30-  3.66] | 0.003 |
| rs12593917 | FAM227B | T | Alt | 62% | 32% | 6% |  |  | 1.80 [1.26-  2.57] | 0.001 |  |  |  |  |
| rs1200503 | FCHO2 | C | Ref | 37% | 47% | 16% | 1.41 [0.98-  2.00] | 0.050 |  |  | 1.52 [1.03-  2.22] | 0.037 |  |  |
| rs3756772 | FRK | T | Alt | 33% | 44% | 23% | 1.44 [0.96-  2.15] | 0.050 | 1.51 [1.04-  2.21] | 0.031 | 1.54 [1.03-  2.29] | 0.034 |  |  |
| rs17855739 | FUT6 | C | Ref | 90% | 9% | 0% | 2.70 [1.33-  5.56] | 0.006 |  |  | 2.04 [1.05-  4.00] | 0.035 |  |  |
| rs869202 | GAB2 | A | Ref | 48% | 42% | 9% | 1.49 [1.05-  2.13] | 0.026 |  |  |  |  |  |  |
| rs4485425 | GALK1, H3F3B | G | Alt | 12% | 42% | 46% | 1.45 [1.02-  2.05] | 0.038 |  |  |  |  |  |  |
| rs11706136 | GLB1 | A | Alt | 19% | 46% | 35% | 1.46 [1.02-  2.10] | 0.037 |  |  |  |  |  |  |
| rs1658972 | GLDC, KDM4C | C | Ref | 83% | 15% | 2% |  |  |  |  | 1.61 [0.97-  2.70] | 0.050 |  |  |
| rs28383223 | HLA-DRB1, HLA- DQA1 | T | Alt | 12% | 46% | 41% |  |  |  |  | 1.49 [1.04-  2.14] | 0.030 | 2.35 [1.26-  4.41] | 0.007 |
| rs61352607 | INHBC | T | Alt | 34% | 49% | 17% |  |  |  |  | 1.93 [1.20-  3.11] | 0.007 | 1.57 [1.03-  2.40] | 0.034 |
| rs56360131 | IRF8, LINC01082 | T | Alt | 76% | 21% | 3% |  |  | 1.66 [1.11-  2.47] | 0.014 |  |  |  |  |
| rs12154248 | JAZF1 | T | Alt | 62% | 32% | 5% |  |  | 1.54 [1.08-  2.19] | 0.017 |  |  | 1.48 [1.00-  2.19] | 0.047 |
| rs717118 | KCNJ2, CASC17 | T | Ref | 16% | 50% | 34% |  |  | 1.49 [1.02-  2.17] | 0.039 |  |  |  |  |

| rs11170319 | KRT78, KRT8 | G | Alt | 11% | 41% | 48% |  |  | 3.14 [1.39-  7.08] | 0.006 |  |  |  |  |
| --- | --- | --- | --- | --- | --- | --- | --- | --- | --- | --- | --- | --- | --- | --- |
| rs7740188 | L3MBTL3 | A | Alt | 7% | 40% | 53% | 1.70 [1.20-  2.42] | 0.003 |  |  | 1.74 [1.17-  2.57] | 0.006 | 2.61 [1.16-  5.84] | 0.020 |
| rs2832277 | LINC00189 | G | Alt | 13% | 41% | 45% |  |  | 2.21 [1.13-  4.33] | 0.020 |  |  | 1.69 [0.95-  2.99] | 0.050 |
| rs17046767 | LINC01191 | G | Ref | 90% | 9% | 0% | 2.48 [1.09-  5.63] | 0.030 |  |  |  |  |  |  |
| rs11086005 | LINC01835 | G | Ref | 24% | 47% | 29% | 1.61 [1.09-  2.44] | 0.018 |  |  | 1.56 [1.01-  2.38] | 0.046 | 1.56 [1.00-  2.44] | 0.048 |
| rs2910953 | LINC02104, LINC00603 | A | Alt | 55% | 38% | 7% | 2.03 [1.04-  3.95] | 0.037 |  |  |  |  |  |  |
| rs10858828 | LINC02458, DUSP6 | G | Ref | 60% | 34% | 6% |  |  | 3.70 [1.11-  12.5] | 0.034 |  |  |  |  |
| rs1800759 | LOC100507053 | T | Ref | 32% | 47% | 21% | 1.49 [1.00-  2.22] | 0.050 |  |  | 1.64 [1.14-  2.38] | 0.009 |  |  |
| rs4841436 | LOC102723313 | C | Ref | 29% | 49% | 21% | 1.54 [1.03-  2.33] | 0.035 |  |  | 1.89 [1.28-  2.78] | 0.001 |  |  |
| rs7189522 | LOC102724084 | T | Ref | 5% | 36% | 59% | 4.97 [2.26-  10.92] | <0.001 |  |  | 3.26 [1.47-  7.14] | 0.004 |  |  |
| rs6577596 | LOC105376975, LOC339862 | C | Ref | 4% | 34% | 62% |  |  |  |  |  |  | 1.56 [1.06-  2.33] | 0.025 |
| rs2126259 | LOC157273 | T | Ref | 10% | 43% | 46% |  |  |  |  | 1.96 [1.1-  3.45] | 0.022 |  |  |
| rs2943654 | LOC646736, MIR5702 | T | Alt | 4% | 30% | 66% | 1.65 [1.08-  2.52] | 0.019 | 5.29 [1.21-  23.13] | 0.027 |  |  |  |  |
| rs10849448 | LTBR | A | Ref | 7% | 35% | 58% |  |  | 1.52 [1.03-  2.22] | 0.033 |  |  | 1.54 [1.10-  2.16] | 0.012 |
| rs6034011 | MACROD2 | C | Alt | 24% | 50% | 26% | 1.58 [1.04-  2.40] | 0.031 | 1.62 [1.00-  2.61] | 0.049 |  |  |  |  |
| rs4821764 | MAFF | A | Alt | 19% | 52% | 29% |  |  |  |  | 1.59 [1.09-  2.33] | 0.017 |  |  |
| rs12544992 | MFHAS1 | G | Alt | 7% | 41% | 51% |  |  |  |  | 1.91 [1.33-  2.73] | <0.001 |  |  |
| rs6589941 | MIR100HG, UBASH3B | G | Alt | 42% | 45% | 13% | 2.00 [1.19-  3.35] | 0.009 |  |  |  |  |  |  |
| rs6916318 | MIR588, RSPO3 | T | Alt | 27% | 48% | 25% | 1.70 [1.14-  2.52] | 0.009 |  |  |  |  |  |  |
| rs4782568 | MLYCD, OSGIN1 | C | Ref | 20% | 50% | 30% |  |  |  |  |  |  | 1.44 [1.00-  2.08] | 0.050 |
| rs17710008 | MYCT1 | A | Alt | 59% | 36% | 5% | 2.35 [1.07-  5.16] | 0.033 |  |  | 3.20 [1.43-  7.15] | 0.005 | 1.49 [1.04-  2.13] | 0.028 |
| rs7209484 | NFE2L1, CBX1 | C | Alt | 35% | 50% | 15% |  |  |  |  | 1.62 [1.01-  2.59] | 0.045 |  |  |
| rs72823014 | NHLRC2, ADRB1 | G | Ref | 85% | 14% | 1% | 1.82 [0.98-  3.33] | 0.050 |  |  | 1.67 [0.96-  2.86] | 0.050 |  |  |
| rs7018885 | NINJ1 | T | Ref | 74% | 23% | 3% | 1.75 [1.15-  2.70] | 0.009 |  |  | 2.17 [1.33-  3.57] | 0.002 |  |  |
| rs2289125 | NOX4 | C | Alt | 10% | 46% | 44% |  |  |  |  | 1.41 [0.99-  2.02] | 0.050 |  |  |
| rs6747874 | NPAS2 | A | Alt | 34% | 45% | 20% | 1.57 [1.03-  2.39] | 0.037 |  |  |  |  |  |  |
| rs503581 | ONECUT2 | G | Ref | 77% | 22% | 1% | 1.75 [1.09-  2.86] | 0.023 |  |  |  |  |  |  |
| rs7117339 | PANX1 | C | Ref | 68% | 28% | 4% | 1.54 [1.04-  2.22] | 0.028 |  |  | 1.66 [1.16-  2.38] | 0.006 |  |  |
| rs7256564 | PEPD | A | Ref | 15% | 44% | 41% | 1.69 [1.06-  2.78] | 0.027 |  |  | 1.79 [1.09-  3.03] | 0.024 |  |  |
| rs45587331 | PIK3AP1 | A | Alt | 74% | 24% | 1% |  |  | 1.60 [1.05-  2.43] | 0.027 |  |  |  |  |
| rs1002436 | PKN2-AS1 | G | Alt | 16% | 46% | 38% |  |  |  |  | 1.51 [1.05-  2.17] | 0.025 |  |  |
| rs738409 | PNPLA3 | G | Alt | 23% | 51% | 26% | 2.12 [1.44-  3.12] | <0.001 |  |  | 1.89 [1.27-  2.81] | 0.002 | 3.28 [1.85-  5.80] | <0.001 |
| rs4135247 | PPARG | G | Ref | 11% | 48% | 41% |  |  |  |  |  |  | 1.45 [1.01-  2.08] | 0.044 |
| rs313839 | PRKD2, STRN4 | G | Alt | 75% | 22% | 2% | 3.27 [1.07-  10.03] | 0.038 |  |  | 4.55 [1.38-  14.93] | 0.013 |  |  |
| rs9372475 | RFX6, VGLL2 | T | Ref | 5% | 33% | 61% |  |  |  |  |  |  | 1.47 [1.00-  2.17] | 0.049 |
| rs2491441 | RGL1 | C | Alt | 70% | 27% | 3% | 4.59 [1.60-  13.23] | 0.005 | 1.48 [0.99-  2.22] | 0.050 | 4.53 [1.55-  13.22] | 0.006 | 1.71 [1.14-  2.56] | 0.010 |

| rs10409243 | S1PR2 | C | Ref | 31% | 51% | 18% |  |  | 2.78 [1.49-  5.26] | 0.001 |  |  |  |  |
| --- | --- | --- | --- | --- | --- | --- | --- | --- | --- | --- | --- | --- | --- | --- |
| rs61980636 | SERPINA6 | T | Alt | 41% | 46% | 13% |  |  |  |  | 1.73 [1.02-  2.94] | 0.042 |  |  |
| rs2727324 | SMARCD2, TCAM1P | C | Alt | 36% | 47% | 17% |  |  |  |  | 1.66 [1.05-  2.64] | 0.030 |  |  |
| rs1917368 | SNX13 | G | Ref | 28% | 49% | 22% |  |  | 1.56 [1.00-  2.38] | 0.050 |  |  |  |  |
| rs1477066 | SOX9-AS1 | C | Alt | 34% | 50% | 16% |  |  |  |  |  |  | 1.75 [1.14-  2.69] | 0.011 |
| rs58504358 | ST3GAL1 | T | Ref | 64% | 33% | 3% |  |  |  |  | 1.52 [1.03-  2.22] | 0.032 |  |  |
| rs112771035 | ST3GAL4 | C | Ref | 93% | 6% | 0% | 2.44 [1.08-  5.56] | 0.033 |  |  | 4.17 [1.25-  14.29] | 0.020 |  |  |
| rs12406530 | ST7L | A | Ref | 43% | 47% | 10% | 1.45 [1.02-  2.04] | 0.036 |  |  | 1.52 [1.04-  2.22] | 0.032 |  |  |
| rs481206 | STARD10 | T | Alt | 48% | 42% | 10% |  |  |  |  | 2.09 [1.18-  3.71] | 0.012 |  |  |
| rs30386 | TBC1D9B | G | Alt | 10% | 37% | 53% |  |  |  |  |  |  | 2.73 [1.33-  5.59] | 0.006 |
| rs2568207 | TCF7L1 | T | Alt | 27% | 51% | 22% |  |  | 1.59 [1.01-  2.50] | 0.045 |  |  |  |  |
| rs58542926 | TM6SF2 | T | Alt | 92% | 8% | 0% |  |  | 2.15 [1.12-  4.12] | 0.021 |  |  | 2.71 [1.40-  5.23] | 0.003 |
| rs7029757 | TOR1B | G | Ref | 90% | 10% | 0% | 2.04 [1.08-  4.00] | 0.029 |  |  | 2.00 [0.94-  4.17] | 0.050 |  |  |
| rs2954027 | TRIB1, LINC00861 | T | Ref | 40% | 47% | 13% |  |  |  |  | 1.59 [1.13-  2.23] | 0.008 | 1.89 [1.13-  3.15] | 0.015 |
| rs134489 | TTC28 | A | Ref | 57% | 37% | 6% |  |  |  |  | 1.52 [1.05-  2.17] | 0.026 |  |  |
| rs998584 | VEGFA, LINC01512 | A | Alt | 19% | 44% | 37% | 1.58 [1.07-  2.34] | 0.021 |  |  | 1.53 [1.06-  2.21] | 0.022 |  |  |
| rs13212562 | VN1R10P, ZNF204P | A | Ref | 77% | 21% | 2% | 1.75 [1.06-  2.86] | 0.026 |  |  | 1.49 [0.96-  2.33] | 0.050 |  |  |
| rs17743415 | ZNF638 | C | Alt | 14% | 47% | 38% | 1.50 [1.02-  2.20] | 0.041 |  |  |  |  |  |  |

Gene annotations were extracted from (Chen et al, 2021). EA: effect allele associated with elevated AST and/or ALT levels in the UKBB, BBJ and CCHC cohorts. Alt: effect allele is the alternate allele. Ref: effect allele is the reference allele. Genotype frequencies in CCHC are shown for wild-type (WT, 2 copies of the reference allele), heterozygous (Het) and homozygous (Hom, 2 copies of the alternate allele) genotypes. AOR [95% CI]: adjusted odds ratios and 95% confidence intervals for elevated AST or elevated ALT for 2 vs. 0-1 effect alleles, and for 1-2 vs. 0 effect alleles. AORs were adjusted for age and gender.

**Supplementary Table S5.** SNPs with a 10% or greater increase in EAF in CCHC compared to UKBB and BBJ, which were also significantly associated with elevated AST and ALT levels in the CCHC.

| **rs ID** | **Chr** | **Position (GRCh38)** | **Gene annotation** | **EA** | **EAF CCHC** | **EAF UKBB** | **EAF BBJ** | **AOR [95% CI] for elevated AST** | **p** | **AOR [95% CI] for elevated ALT** | **p** |
| --- | --- | --- | --- | --- | --- | --- | --- | --- | --- | --- | --- |
| rs738409 | 22 | 43928847 | PNPLA3 (e) | G | 52% | 22% | 45% | 2.12 [1.44-3.12] | <0.001 | 3.28 [1.85-5.80] | <0.001 |
| rs2950388 | 12 | 56639569 | ATP5B (i) | C | 79% | 72% | 67% | 1.54 [1.03-2.33] | 0.034 | 1.75 [1.22-2.56] | 0.003 |
| rs7018885 | 9 | 93130444 | NINJ1 (i) | T | 86% | 75% | 78% | 1.75 [1.15-2.70] | 0.009 | 2.17 [1.33-3.57] | 0.002 |
| rs1200503 | 5 | 73055235 | FCHO2 (i) | C | 61% | 53% | 46% | 1.41 [0.98-2.00] | 0.050 | 1.52 [1.03-2.22] | 0.037 |
| rs1800759 | 4 | 99144358 | LOC100507053 (i) | T | 56% | 39% | 16% | 1.49 [1.00-2.22] | 0.050 | 1.64 [1.14-2.38] | 0.009 |
| rs17710008 | 6 | 152721900 | MYCT1 (e) | A | 23% | 19% | 12% | 2.35 [1.07-5.16] | 0.033 | 3.20 [1.43-7.15] | 0.005 |
| rs17743415 | 2 | 71335373 | ZNF638 (i) | C | 63% | 57% | 18% | 1.50 [1.02-2.20] | 0.041 |  |  |
| rs28383223 | 6 | 32615700 | HLA-DRB1, HLA-DQA1 (inter) | T | 65% | 58% | 46% |  |  | 2.35 [1.26-4.41] | 0.007 |
| rs4638642 | 17 | 31312307 | EVI2B, NF1 (i) | G | 63% | 39% | 50% |  |  | 2.18 [1.30-3.66] | 0.003 |
| rs61352607 | 12 | 57445390 | INHBC (i) | T | 42% | 24% | 7% |  |  | 1.93 [1.20-3.11] | 0.007 |
| rs2126259 | 8 | 9327636 | LOC157273 (i) | T | 32% | 10% | 1% |  |  | 1.96 [1.1-3.45] | 0.022 |
| rs2954027 | 8 | 125473052 | TRIB1, LINC00861 (inter) | T | 63% | 53% | 51% |  |  | 1.89 [1.13-3.15] | 0.015 |
| rs7209484 | 17 | 48065680 | NFE2L1, CBX1 (inter) | C | 40% | 24% | 22% |  |  | 1.62 [1.01-2.59] | 0.045 |

Annotations: (e) exonic, (i) intronic, (u) upstream, (d) downstream, (inter) intergenic, (UTR) untranslated region; EA: effect allele; EAF: effect allele frequency; CCHC: Cameron County Hispanic Cohort; UKBB: UK BioBank; BBJ: BioBank Japan; AOR [95% CI]: adjusted odds ratios 95% confidence intervals. Annotations and EAF in UKBB and BBJ were extracted from (Chen et al, 2021). AORs were adjusted for age and gender.

**Supplementary Table S6.** SNPs with strong associations with clinical outcomes of interest, as listed in Figures 3 and 4. For elevated AST and ALT, rs738409 (PNPLA3) and SNPs with an association stronger or similar to rs738409 are shown.

| **Clinical outcome** | **rsID** | **Chr** | **Position (GRCh38)** | **EA** | **Gene annotation** | **AOR [95% CI]** | **p** |
| --- | --- | --- | --- | --- | --- | --- | --- |
| **Elevated AST** | rs2943654 | 2 | 226248038 | T | LOC646736, MIR5702 (inter) | 5.29 [1.21-23.13] | 0.027 |
|  | rs7189522 | 16 | 80464197 | T | LOC102724084 (i) | 4.97 [2.26-10.92] | <0.001 |
|  | rs2491441 | 1 | 183833125 | C | RGL1 (i) | 4.59 [1.60-13.23] | 0.005 |
|  | rs62292950 | 3 | 132479151 | T | DNAJC13 (i) | 3.85 [1.11-12.5] | 0.033 |
|  | rs10858828 | 12 | 89147879 | G | LINC02458, DUSP6 (inter) | 3.70 [1.11-12.5] | 0.034 |
|  | rs313839 | 19 | 46718300 | G | PRKD2, STRN4 (inter) | 3.27 [1.07-10.03] | 0.038 |
|  | rs11170319 | 12 | 52891302 | G | KRT78, KRT8 (inter) | 3.14 [1.39-7.08] | 0.006 |
|  | rs10409243 | 19 | 10222312 | C | S1PR2 (UTR) | 2.78 [1.49-5.26] | 0.001 |
|  | rs17855739 | 19 | 5831829 | C | FUT6 (e) | 2.70 [1.33-5.56] | 0.006 |
|  | rs421491 | 13 | 110647653 | T | CARS2 (i) | 2.63 [1.25-5.56] | 0.010 |
|  | rs1801690 | 17 | 66212167 | G | APOH (e) | 2.58 [0.97-6.87] | 0.050 |
|  | rs17046767 | 2 | 113999632 | G | LINC01191 (i) | 2.48 [1.09-5.63] | 0.030 |
|  | rs112771035 | 11 | 126355981 | C | ST3GAL4 (UTR) | 2.44 [1.08-5.56] | 0.033 |
|  | rs17710008 | 6 | 152721900 | A | MYCT1 (e) | 2.35 [1.07-5.16] | 0.033 |
|  | rs2832277 | 21 | 29236564 | G | LINC00189 (i) | 2.21 [1.13-4.33] | 0.020 |
|  | rs58542926 | 19 | 19268740 | T | TM6SF2 (e) | 2.15 [1.12-4.12] | 0.021 |
|  | rs738409 | 22 | 43928847 | G | PNPLA3 (e) | 2.12 [1.44-3.12] | <0.001 |
| **Elevated ALT** | rs2519093 | 9 | 133266456 | C | ABO (i) | 8.05 [1.01-64.36] | 0.049 |
|  | rs313839 | 19 | 46718300 | G | PRKD2, STRN4 (inter) | 4.55 [1.38-14.93] | 0.013 |
|  | rs2491441 | 1 | 183833125 | C | RGL1 (i) | 4.53 [1.55-13.22] | 0.006 |
|  | rs112771035 | 11 | 126355981 | C | ST3GAL4 (UTR) | 4.17 [1.25-14.29] | 0.020 |
|  | rs738409 | 22 | 43928847 | G | PNPLA3 (e) | 3.28 [1.85-5.80] | <0.001 |
|  | rs7189522 | 16 | 80464197 | T | LOC102724084 (i) | 3.26 [1.47-7.14] | 0.004 |
|  | rs17710008 | 6 | 152721900 | A | MYCT1 (e) | 3.20 [1.43-7.15] | 0.005 |
| **Liver steatosis** | rs2491441 | 1 | 183833125 | C | RGL1 (i) | 8.62 [1.11-66.86] | 0.039 |
|  | rs2519093 | 9 | 133266456 | C | ABO (i) | 8.51 [1.01-71.72] | 0.049 |
|  | rs17710008 | 6 | 152721900 | A | MYCT1 (e) | 2.75 [1.00-7.57] | 0.050 |
|  | rs10858828 | 12 | 89147879 | G | LINC02458, DUSP6 (inter) | 2.00 [0.95-4.23] | 0.069 |
|  | rs7740188 | 6 | 130024960 | A | L3MBTL3 (i) | 1.95 [1.01-3.77] | 0.046 |
|  | rs9372475 | 6 | 117180825 | T | RFX6, VGLL2 (inter) | 1.61 [1.11-2.35] | 0.013 |
|  | rs12129745 | 1 | 28245806 | G | ATPIF1, SESN2 (inter) | 1.58 [1.10-2.29] | 0.014 |
|  | rs12154248 | 7 | 28152756 | T | JAZF1 (i) | 1.52 [1.05-2.21] | 0.027 |
|  | rs17743415 | 2 | 71335373 | C | ZNF638 (i) | 1.51 [1.04-2.19] | 0.032 |
| **Liver fibrosis** | rs1801690 | 17 | 66212167 | G | APOH (e) | 5.26 [1.85-14.95] | 0.002 |
|  | rs10858828 | 12 | 89147879 | G | LINC02458, DUSP6 (inter) | 5.00 [0.67-37.41] | 0.117 |
|  | rs10409243 | 19 | 10222312 | C | S1PR2 (UTR) | 4.55 [1.60-12.90] | 0.004 |
|  | rs2491441 | 1 | 183833125 | C | RGL1 (i) | 3.12 [0.92-10.57] | 0.067 |
|  | rs58542926 | 19 | 19268740 | T | TM6SF2 (e) | 2.29 [1.02-5.12] | 0.044 |
|  | rs1800759 | 4 | 99144358 | T | LOC100507053 (i) | 2.18 [1.28-3.70] | 0.004 |
|  | rs13212562 | 6 | 27332531 | A | VN1R10P, ZNF204P (inter) | 2.17 [1.04-4.54] | 0.040 |
| **Advanced liver fibrosis** | rs10409243 | 19 | 10222312 | C | S1PR2 (UTR) | 5.12 [1.20-21.81] | 0.027 |
|  | rs2491441 | 1 | 183833125 | C | RGL1 (i) | 4.06 [1.04-15.78] | 0.043 |
|  | rs1801690 | 17 | 66212167 | G | APOH (e) | 3.57 [1.08-11.83] | 0.037 |
|  | rs313839 | 19 | 46718300 | G | PRKD2, STRN4 (inter) | 3.05 [0.62-14.85] | 0.168 |
|  | rs11240351 | 1 | 205075211 | G | CNTN2 (UTR) | 2.39 [1.22-4.69] | 0.011 |
|  | rs738409 | 22 | 43928847 | G | PNPLA3 (e) | 2.16 [1.09-4.27] | 0.027 |
|  | rs1800759 | 4 | 99144358 | T | LOC100507053 (i) | 2.07 [1.05-4.10] | 0.036 |
| **Obesity** | rs17710008 | 6 | 152721900 | A | MYCT1 (e) | 3.55 [1.39-9.04] | 0.008 |
|  | rs17046767 | 2 | 113999632 | G | LINC01191 (i) | 2.51 [1.36-4.65] | 0.003 |
|  | rs10931283 | 2 | 187346716 | T | CALCRL (i) | 1.80 [1.13-2.86] | 0.013 |
|  | rs72823014 | 10 | 114026477 | G | NHLRC2, ADRB1 (inter) | 1.67 [1.02-2.72] | 0.041 |
|  | rs134489 | 22 | 28333779 | A | TTC28 (i) | 1.57 [1.11-2.22] | 0.011 |
|  | rs11706136 | 3 | 33056563 | A | GLB1 (i) | 1.51 [1.05-2.16] | 0.026 |
| **Diabetes** | rs2491441 | 1 | 183833125 | C | RGL1 (i) | 3.32 [1.12-9.84] | 0.031 |
|  | rs112771035 | 11 | 126355981 | C | ST3GAL4 (UTR) | 2.84 [1.19-6.82] | 0.019 |
|  | rs17710008 | 6 | 152721900 | A | MYCT1 (e) | 2.55 [1.09-5.97] | 0.031 |
|  | rs12544992 | 8 | 8804171 | G | MFHAS1 (i) | 2.03 [1.38-2.99] | <0.001 |
|  | rs2126259 | 8 | 9327636 | T | LOC157273 (i) | 1.81 [1.00-3.29] | 0.049 |
|  | rs3756772 | 6 | 116003979 | T | FRK (e) | 1.68 [1.09-2.61] | 0.020 |
|  | rs1917368 | 7 | 17872129 | G | SNX13 (i) | 1.64 [1.02-2.63] | 0.041 |
|  | rs1800759 | 4 | 99144358 | T | LOC100507053 (i) | 1.58 [1.06-2.35] | 0.026 |
|  | rs4841436 | 8 | 10736964 | C | LOC102723313 (i) | 1.54 [1.03-2.30] | 0.037 |

Annotations: (e) exonic, (i) intronic, (u) upstream, (d) downstream, (inter) intergenic, (UTR) untranslated region; EA: effect allele. AOR [95% CI]: adjusted odds ratios 95% confidence intervals for clinical outcomes. AORs were adjusted for age and gender.

**Supplementary Table S7.** Gene Expression Results from PhenoScanner.

| **Variants** | **a1** | **a2** | **Proxy rsID** | **a1** | **a2** | **r2** | **Tissue** | **Expressed Gene** | **p** | **Direction** |
| --- | --- | --- | --- | --- | --- | --- | --- | --- | --- | --- |
| rs2519093 (ABO) | C | T | rs532436 | G | A | 0.99 | Adipose subcutaneous | ABO | 3.88E-08 | - |
|  |  |  | rs635634 | C | T | 0.99 | Whole blood | ABO | 4.18E-280 | + |
|  |  |  |  |  |  |  | Whole blood | BPI | 2.65E-06 | + |
|  |  |  |  |  |  |  | Whole blood | CACFD1 | 6.95E-13 | + |
|  |  |  |  |  |  |  | Whole blood | CEACAM8 | 6.40E-06 | + |
|  |  |  |  |  |  |  | Whole blood | CLEC4C | 4.56E-07 | + |
|  |  |  |  |  |  |  | Whole blood | CTSG | 2.35E-07 | + |
|  |  |  |  |  |  |  | Whole blood | DBN1 | 5.64E-06 | + |
|  |  |  |  |  |  |  | Whole blood | GBGT1 | 6.01E-26 | + |
|  |  |  |  |  |  |  | Whole blood | H19 | 3.49E-10 | - |
|  |  |  |  |  |  |  | Whole blood | MED22 | 7.58E-08 | - |
|  |  |  |  |  |  |  | Whole blood | MMP9 | 7.54E-06 | + |
|  |  |  |  |  |  |  | Whole blood | SURF1 | 2.40E-30 | + |
| rs7041363 (AKNA) | C | G | rs4979373 | C | T | 0.99 | Colon sigmoid | AKNA | 7.45E-11 | + |
|  |  |  | rs10733608 | G | T | 0.95 | Colon transverse | AKNA | 1.34E-07 | + |
|  |  |  | rs4979371 | C | T | 0.97 | Liver | AKNA | 3.90E-06 | + |
|  |  |  | rs7032795 | T | C | 0.94 | Neutrophils | AKNA | 6.50E-13 | - |
|  |  |  |  |  |  |  | Whole blood | AKNA | 1.66E-28 | - |
|  |  |  |  |  |  |  | Whole blood | COL27A1 | 8.13E-06 | - |
|  |  |  |  |  |  |  | Whole blood | ORM2 | 1.12E-32 | - |
| rs11704551 (AP1B1) | C | T | rs4611747 | C | T | 0.72 | Adipose visceral omentum | AP1B1 | 2.43E-06 | - |
|  |  |  | rs8140836 | G | A | 1.00 | Liver | AP1B1 | 6.05E-41 | NA |
|  |  |  | rs113886205 | C | T | 0.71 | Whole blood | AP1B1 | 6.70E-25 | - |
|  |  |  | rs34680030 | C | T | 0.71 | Whole blood | GAS2L1 | 2.22E-42 | - |
|  |  |  |  |  |  |  | Whole blood | NEFH | 1.69E-06 | + |
|  |  |  |  |  |  |  | Whole blood | NIPSNAP1 | 2.41E-09 | - |
|  |  |  |  |  |  |  | Whole blood | RASL10A | 8.89E-08 | - |
|  |  |  |  |  |  |  | Whole blood | SF3A1 | 1.49E-17 | - |
| rs1801690 (APOH) | G | C | rs1801690 | G | C | 1.00 | Whole blood | PRKCA | 6.13E-24 | - |
|  |  |  | rs144579769 | T | C | 1.00 |  |  |  |  |

|  |  |  | rs76452927 | A | G | 0.83 |  |  |  |  |
| --- | --- | --- | --- | --- | --- | --- | --- | --- | --- | --- |
| rs1664781 (ARL15) | A | G | rs4865796 | A | G | 0.97 | Adipose subcutaneous | FST | 5.97E-06 | - |
|  |  |  | rs702634 | A | G | 0.94 |  |  |  |  |
| rs9616810 (ARSA, SHANK3) | C | T | rs6010040 | T | G | 0.99 | Whole blood | ARSA | 1.31E-47 | - |
|  |  |  | rs6010042 | G | A | 0.99 | Whole blood | CPT1B; CHKB- CPT1B | 1.24E-06 | + |
|  |  |  | rs12484068 | C | T | 0.99 | Spleen | MAPK12 | 5.46E-06 | - |
|  |  |  | rs68174505 | G | A | 0.96 | Whole blood | RABL2B | 3.64E-08 | - |
|  |  |  |  |  |  |  | Whole blood | SCO2 | 1.40E-09 | - |
|  |  |  |  |  |  |  | Adipose subcutaneous | SHANK3 | 1.26E-11 | - |
|  |  |  |  |  |  |  | Adipose visceral omentum | SHANK3 | 1.91E-06 | - |
|  |  |  |  |  |  |  | Colon transverse | SHANK3 | 9.61E-06 | - |
|  |  |  |  |  |  |  | Liver | SHANK3 | 2.22E-14 | NA |
|  |  |  |  |  |  |  | Visceral abdominal fat | SHANK3 | 9.82E-15 | NA |
| rs2950388 (ATP5B) | C | T | rs9634246 | A | G | 0.99 | Whole blood | ABCC3 | 5.75E-06 | - |
|  |  |  | rs2950394 | T | C | 0.99 | Whole blood | AQP10 | 2.94E-19 | - |
|  |  |  | rs941207 | C | G | 0.91 | Whole blood | ATP5B | 3.61E-06 | + |
|  |  |  | rs7315472 | G | C | 0.89 | Whole blood | BAMBI | 1.04E-06 | + |
|  |  |  |  |  |  |  | Whole blood | BAZ2A | 1.19E-18 | - |
|  |  |  |  |  |  |  | Whole blood | C1orf198 | 7.99E-10 | - |
|  |  |  |  |  |  |  | Whole blood | C21orf7 | 2.01E-16 | + |
|  |  |  |  |  |  |  | Whole blood | CA2 | 1.39E-08 | + |
|  |  |  |  |  |  |  | Whole blood | CD9 | 7.70E-06 | - |
|  |  |  |  |  |  |  | Whole blood | CDK2AP1 | 2.35E-06 | - |
|  |  |  |  |  |  |  | Whole blood | CLDN5 | 7.97E-11 | - |
|  |  |  |  |  |  |  | Whole blood | CTDSPL | 1.05E-06 | - |
|  |  |  |  |  |  |  | Whole blood | CTTN | 4.74E-10 | - |
|  |  |  |  |  |  |  | Whole blood | ENKUR | 2.03E-30 | + |
|  |  |  |  |  |  |  | Whole blood | ESAM | 8.46E-09 | - |
|  |  |  |  |  |  |  | Whole blood | GLS2; MIP; SPRYD4 | 2.56E-07 | - |
|  |  |  |  |  |  |  | Whole blood | GRB14 | 1.24E-12 | - |
|  |  |  |  |  |  |  | Whole blood | HIST1H2AG | 2.24E-09 | + |

|  |  |  |  |  |  |  | Whole blood | HIST1H2BG | 2.22E-06 | + |
| --- | --- | --- | --- | --- | --- | --- | --- | --- | --- | --- |
|  |  |  |  |  |  |  | Whole blood | ITGA2B | 2.85E-06 | - |
|  |  |  |  |  |  |  | Whole blood | ITGB3 | 1.19E-11 | - |
|  |  |  |  |  |  |  | Whole blood | JAM3 | 4.51E-06 | - |
|  |  |  |  |  |  |  | Whole blood | MGLL | 5.29E-08 | - |
|  |  |  |  |  |  |  | Whole blood | MYLK | 1.27E-06 | - |
|  |  |  |  |  |  |  | Whole blood | NACA | 2.09E-32 | - |
|  |  |  |  |  |  |  | Whole blood | NACA3P | 3.27E-310 | + |
|  |  |  |  |  |  |  | Whole blood | NACAP1 | 6.74E-68 | - |
|  |  |  |  |  |  |  | Whole blood | NFIB | 3.20E-06 | - |
|  |  |  |  |  |  |  | Whole blood | NRGN | 6.68E-06 | - |
|  |  |  |  |  |  |  | Whole blood | PARVB | 4.92E-08 | - |
|  |  |  |  |  |  |  | Whole blood | PDE5A | 8.68E-09 | - |
|  |  |  |  |  |  |  | Whole blood | PPBP | 9.13E-07 | - |
|  |  |  |  |  |  |  | Whole blood | PRIM1 | 6.28E-99 | - |
|  |  |  |  |  |  |  | Whole blood | PTGES3 | 1.43E-13 | - |
|  |  |  |  |  |  |  | Whole blood | RAB11A | 1.59E-06 | - |
|  |  |  |  |  |  |  | Whole blood | RAB6B | 1.52E-06 | - |
|  |  |  |  |  |  |  | Adipose subcutaneous | RBMS2 | 6.95E-31 | - |
|  |  |  |  |  |  |  | Whole blood | RBMS2 | 2.68E-278 | - |
|  |  |  |  |  |  |  | Whole blood | SAMD14 | 5.46E-06 | - |
|  |  |  |  |  |  |  | Whole blood | SH3BGRL2 | 8.76E-08 | - |
|  |  |  |  |  |  |  | Whole blood | SPARC | 5.37E-06 | - |
|  |  |  |  |  |  |  | Whole blood | SPOCD1 | 2.94E-40 | - |
|  |  |  |  |  |  |  | Whole blood | SPRYD4 | 3.14E-27 | - |
|  |  |  |  |  |  |  | Whole blood | TMEM158 | 2.57E-07 | - |
|  |  |  |  |  |  |  | Whole blood | TSPAN9 | 1.98E-08 | - |
|  |  |  |  |  |  |  | Whole blood | VWA7 | 8.70E-06 | + |
| rs12129745 (ATPIF1, SESN2) | C | G | rs111715719 | G | T | 0.73 | Whole blood | AL353354.1 | 1.30E-09 | - |
|  |  |  | rs61786981 | A | G | 0.73 | Adipose subcutaneous | ATPIF1 | 6.13E-18 | - |
|  |  |  | rs489620 | G | C | 0.75 | Colon sigmoid | ATPIF1 | 1.47E-11 | - |
|  |  |  | rs8559 | G | A | 0.75 | Monocytes | ATPIF1 | 1.63E-10 | - |
|  |  |  |  |  |  |  | Whole blood | ATPIF1 | 6.82E-200 | - |

|  |  |  |  |  |  |  | Adipose subcutaneous | DNAJC8 | 3.51E-10 | - |
| --- | --- | --- | --- | --- | --- | --- | --- | --- | --- | --- |
|  |  |  |  |  |  |  | Monocytes | DNAJC8 | 5.45E-33 | - |
|  |  |  |  |  |  |  | Neutrophils | DNAJC8 | 4.86E-17 | - |
|  |  |  |  |  |  |  | T cells | DNAJC8 | 1.04E-07 | + |
|  |  |  |  |  |  |  | Whole blood | DNAJC8 | 9.69E-56 | - |
|  |  |  |  |  |  |  | Whole blood | EYA3 | 1.98E-11 | + |
|  |  |  |  |  |  |  | Whole blood | PHACTR4 | 5.50E-29 | - |
|  |  |  |  |  |  |  | Whole blood | RPA2 | 5.10E-08 | + |
|  |  |  |  |  |  |  | T cells | SESN2 | 3.54E-10 | + |
|  |  |  |  |  |  |  | Whole blood | SESN2 | 1.75E-06 | - |
|  |  |  |  |  |  |  | Whole blood | SMPDL3B | 6.93E-07 | - |
|  |  |  |  |  |  |  | Whole blood | SNHG12 | 7.91E-17 | - |
| rs196210 (BAG3, INPP5F) | C | G | rs196223 | G | A | 0.99 | Whole blood | BAG3 | 2.33E-11 | + |
|  |  |  | rs196217 | C | T | 0.99 | Whole blood | INPP5F | 5.14E-11 | - |
|  |  |  | rs196225 | A | G | 0.99 | Whole blood | MCMBP | 1.00E-215 | + |
|  |  |  | rs196227 | G | A | 0.99 | Whole blood | SEC23IP | 4.27E-10 | - |
| rs11054848 (BORCS5) | A | G | rs12818821 | C | T | 0.87 | Whole blood | DUSP16 | 3.65E-07 | - |
|  |  |  | rs10772556 | C | T | 0.98 | Monocytes | LOH12CR1 | 1.21E-10 | + |
|  |  |  | rs3741800 | C | T | 0.86 | Neutrophils | LOH12CR1 | 1.73E-09 | + |
|  |  |  | rs10772552 | C | T | 0.86 | Whole blood | LOH12CR1 | 1.74E-53 | + |
|  |  |  |  |  |  |  | Whole blood | MANSC1 | 2.39E-06 | - |
|  |  |  |  |  |  |  | Whole blood | RP11-253I19.3 | 8.07E-06 | + |
| rs4484649 (C8orf74, SOX7) | A | C | rs4292651 | A | C | 0.68 | Whole blood | AF131215.9 | 3.69E-77 | - |
|  |  |  | rs13278953 | A | C | 0.67 | Whole blood | BLK | 5.85E-66 | + |
|  |  |  | rs6998295 | A | C | 0.66 | Whole blood | CTSB | 7.79E-47 | - |
|  |  |  |  |  |  |  | Whole blood | ERI1 | 7.65E-16 | - |
|  |  |  |  |  |  |  | Whole blood | FAM167A | 7.05E-96 | - |
|  |  |  |  |  |  |  | Whole blood | MSRA | 1.64E-67 | + |
|  |  |  |  |  |  |  | Whole blood | RP11- 148O21.4 | 5.88E-22 | + |
|  |  |  |  |  |  |  | Whole blood | RP11-981G7.2 | 4.89E-06 | - |
|  |  |  |  |  |  |  | Adipose subcutaneous | RP1L1 | 1.49E-09 | - |
|  |  |  |  |  |  |  | Whole blood | RP1L1 | 1.82E-15 | - |
|  |  |  |  |  |  |  | Whole blood | SLC35G5 | 2.76E-10 | + |

|  |  |  |  |  |  |  | Liver | SOX7 | 9.80E-12 | NA |
| --- | --- | --- | --- | --- | --- | --- | --- | --- | --- | --- |
|  |  |  |  |  |  |  | Whole blood | XKR6 | 2.53E-10 | - |
| rs10931283 (CALCRL) | C | T | rs10931284 | C | T | 0.94 | Whole blood | BACE1 | 6.24E-06 | + |
|  |  |  | rs10931285 | T | A | 0.73 | Monocytes | CALCRL | 3.80E-08 | - |
|  |  |  | rs11904730 | T | C | 0.73 | Whole blood | CALCRL | 1.04E-108 | - |
|  |  |  | rs1828546 | C | G | 0.73 |  |  |  |  |
| rs421491 (CARS2) | C | T | rs450847 | G | C | 1.00 | Whole blood | CARKD | 6.13E-39 | - |
|  |  |  | rs2986091 | T | C | 0.98 | Adipose subcutaneous | CARS2 | 5.29E-17 | - |
|  |  |  | rs2986328 | G | A | 0.98 | Adipose visceral omentum | CARS2 | 1.05E-16 | - |
|  |  |  | rs2296661 | T | C | 0.93 | Colon sigmoid | CARS2 | 2.52E-10 | - |
|  |  |  |  |  |  |  | Colon transverse | CARS2 | 2.79E-10 | - |
|  |  |  |  |  |  |  | Monocytes | CARS2 | 4.82E-09 | - |
|  |  |  |  |  |  |  | Small intestine terminal ileum | CARS2 | 3.96E-09 | - |
|  |  |  |  |  |  |  | Spleen | CARS2 | 1.07E-08 | - |
|  |  |  |  |  |  |  | Whole blood | CARS2 | 1.05E-87 | - |
| rs12979658 (CLEC4GP1, EVI5L) | A | G | rs149098725 | G | G C | 0.88 | Adipose visceral omentum | EVI5L | 6.72E-07 | - |
|  |  |  | rs11260035 | G | A | 0.98 | Liver | EVI5L | 1.32E-06 | - |
|  |  |  | rs11672003 | G | A | 0.98 | Whole blood | EVI5L | 3.75E-22 | + |
|  |  |  | rs12984133 | G | A | 0.87 |  |  |  |  |
| rs11240351 (CNTN2) | A | G | rs10900448 | G | A | 0.72 | Adipose subcutaneous | DSTYK | 2.07E-12 | + |
|  |  |  | rs1572993 | G | A | 0.89 | Adipose visceral omentum | DSTYK | 4.96E-06 | + |
|  |  |  | rs12048743 | C | G | 0.85 | Colon sigmoid | DSTYK | 8.61E-06 | + |
|  |  |  | rs11240358 | G | A | 0.72 | Colon transverse | DSTYK | 2.43E-06 | + |
|  |  |  |  |  |  |  | Monocytes | DSTYK | 2.11E-11 | - |
|  |  |  |  |  |  |  | Whole blood | DSTYK | 3.27E-310 | - |
|  |  |  |  |  |  |  | Whole blood | NUAK2 | 1.63E-07 | + |
|  |  |  |  |  |  |  | Monocytes | RBBP5 | 3.80E-11 | - |
|  |  |  |  |  |  |  | Whole blood | RBBP5 | 3.25E-13 | - |
|  |  |  |  |  |  |  | Whole blood | TMCC2 | 2.78E-15 | - |
|  |  |  |  |  |  |  | Adipose subcutaneous | TMEM81 | 1.97E-06 | - |
|  |  |  |  |  |  |  | Monocytes | TMEM81 | 1.84E-09 | - |

|  |  |  |  |  |  |  | Whole blood | TMEM81 | 6.83E-24 | - |
| --- | --- | --- | --- | --- | --- | --- | --- | --- | --- | --- |
| rs4766568 (CUX2) | C | T | rs1966303 | T | C | 0.98 | Whole blood | ACAD10 | 6.39E-10 | + |
|  |  |  | rs3809278 | A | C | 0.89 | Whole blood | ADAM1A | 1.43E-16 | - |
|  |  |  | rs11611514 | A | G | 0.84 | Whole blood | ADAM1B | 6.43E-11 | + |
|  |  |  | rs35236844 | G | G A | 0.83 | Whole blood | ALDH2 | 1.84E-10 | + |
|  |  |  |  |  |  |  | Whole blood | ATXN2 | 2.76E-06 | + |
|  |  |  |  |  |  |  | Whole blood | ERP29 | 3.24E-12 | - |
|  |  |  |  |  |  |  | Whole blood | FAM109A | 7.79E-43 | + |
|  |  |  |  |  |  |  | Whole blood | MAPKAPK5 | 9.53E-24 | + |
|  |  |  |  |  |  |  | Whole blood | MAPKAPK5- AS1 | 4.48E-75 | + |
|  |  |  |  |  |  |  | Whole blood | NAA25 | 4.35E-22 | + |
|  |  |  |  |  |  |  | Whole blood | RP3-462E2.3 | 3.52E-08 | + |
|  |  |  |  |  |  |  | Whole blood | RP3-462E2.5 | 2.74E-08 | - |
|  |  |  |  |  |  |  | Whole blood | RP3-473L9.4 | 4.00E-24 | + |
|  |  |  |  |  |  |  | Whole blood | SH2B3 | 5.08E-42 | - |
|  |  |  |  |  |  |  | Adipose visceral omentum | TMEM116 | 1.61E-07 | + |
|  |  |  |  |  |  |  | Whole blood | TMEM116 | 3.35E-133 | + |
| rs62292950 (DNAJC13) | G | T | rs62292896 | A | G | 0.85 | Whole blood | ACPP | 7.16E-12 | - |
|  |  |  | rs62292953 | A | T | 0.85 | Adipose subcutaneous | DNAJC13 | 7.25E-06 | - |
|  |  |  | rs16839317 | C | T | 0.79 | Monocytes | DNAJC13 | 3.15E-06 | - |
|  |  |  |  |  |  |  | Whole blood | DNAJC13 | 2.44E-46 | - |
|  |  |  |  |  |  |  | Adipose subcutaneous | NPHP3 | 1.04E-10 | - |
|  |  |  |  |  |  |  | Neutrophils | NPHP3 | 5.26E-07 | - |
|  |  |  |  |  |  |  | Whole blood | NPHP3 | 4.26E-131 | - |
|  |  |  |  |  |  |  | Whole blood | RHBDD3 | 6.83E-06 | + |
|  |  |  |  |  |  |  | Whole blood | UBA5 | 5.11E-06 | + |
| rs6088521 (DYNLRB1, MAP1LC3A ) | A | C | rs13044413 | A | G | 0.67 | Whole blood | ACSS2 | 3.37E-07 | - |
|  |  |  | rs6120663 | C | A | 0.64 | Whole blood | CHMP4B | 4.20E-06 | + |
|  |  |  | rs6059872 | C | A | 0.62 | Whole blood | EDEM2 | 3.82E-65 | + |
|  |  |  | rs2424992 | C | T | 0.61 | Whole blood | EIF2S2 | 1.60E-55 | - |
|  |  |  |  |  |  |  | Whole blood | EIF6 | 7.02E-59 | - |

|  |  |  |  |  |  |  | Adipose subcutaneous | GGT7 | 8.91E-07 | + |
| --- | --- | --- | --- | --- | --- | --- | --- | --- | --- | --- |
|  |  |  |  |  |  |  | Whole blood | GGT7 | 7.04E-53 | + |
|  |  |  |  |  |  |  | Adipose subcutaneous | GGTL3 | 6.11E-25 | + |
|  |  |  |  |  |  |  | Monocytes | GGTL3 | 3.14E-40 | NA |
|  |  |  |  |  |  |  | Adipose subcutaneous | ITCH | 1.07E-08 | + |
|  |  |  |  |  |  |  | Whole blood | ITCH | 6.42E-108 | - |
|  |  |  |  |  |  |  | Adipose subcutaneous | MAP1LC3A | 4.55E-29 | - |
|  |  |  |  |  |  |  | Adipose visceral omentum | MAP1LC3A | 9.03E-21 | - |
|  |  |  |  |  |  |  | Colon sigmoid | MAP1LC3A | 1.87E-11 | - |
|  |  |  |  |  |  |  | Colon transverse | MAP1LC3A | 3.03E-10 | - |
|  |  |  |  |  |  |  | Liver | MAP1LC3A | 3.47E-06 | - |
|  |  |  |  |  |  |  | Monocytes | MAP1LC3A | 2.69E-08 | NA |
|  |  |  |  |  |  |  | Small intestine terminal ileum | MAP1LC3A | 2.28E-07 | - |
|  |  |  |  |  |  |  | Whole blood | MAP1LC3A | 8.98E-183 | - |
|  |  |  |  |  |  |  | Whole blood | MMP24 | 8.13E-11 | + |
|  |  |  |  |  |  |  | Whole blood | MMP24-AS1 | 1.97E-06 | + |
|  |  |  |  |  |  |  | Whole blood | MYH7B | 1.25E-19 | - |
|  |  |  |  |  |  |  | Whole blood | NCOA6 | 1.75E-07 | + |
|  |  |  |  |  |  |  | Whole blood | PIGU | 2.09E-08 | - |
|  |  |  |  |  |  |  | Whole blood | PROCR | 4.19E-33 | + |
|  |  |  |  |  |  |  | Whole blood | RP1-64K7.4 | 8.33E-09 | + |
|  |  |  |  |  |  |  | Whole blood | RP4-614O4.12 | 8.23E-38 | + |
|  |  |  |  |  |  |  | Whole blood | RP5- 1125A11.1 | 1.44E-14 | + |
|  |  |  |  |  |  |  | Whole blood | TRPC4AP | 9.76E-16 | + |
|  |  |  |  |  |  |  | Whole blood | UQCC | 2.72E-56 | + |
| rs4638642 (EVI2B, NF1) | G | C | rs10512434 | C | T | 1.00 | Whole blood | CRLF3 | 6.95E-07 | - |
|  |  |  | rs12602834 | G | A | 0.99 | Monocytes | EVI2A | 9.78E-11 | - |
|  |  |  | rs7218930 | A | G | 0.99 | Neutrophils | EVI2A | 5.09E-18 | - |
|  |  |  | rs7503922 | C | A | 0.99 | Spleen | EVI2A | 9.60E-09 | - |
|  |  |  |  |  |  |  | T cells | EVI2A | 2.51E-16 | - |
|  |  |  |  |  |  |  | Whole blood | EVI2A | 3.27E-310 | - |
|  |  |  |  |  |  |  | Whole blood | EVI2B | 1.28E-34 | - |

|  |  |  |  |  |  |  | Adipose subcutaneous | NF1 | 7.22E-06 | + |
| --- | --- | --- | --- | --- | --- | --- | --- | --- | --- | --- |
|  |  |  |  |  |  |  | Whole blood | NF1 | 4.07E-56 | + |
|  |  |  |  |  |  |  | Spleen | OMG | 1.83E-11 | + |
|  |  |  |  |  |  |  | Whole blood | OMG | 1.20E-12 | + |
|  |  |  |  |  |  |  | Whole blood | RAB11FIP4 | 2.19E-06 | - |
|  |  |  |  |  |  |  | Whole blood | TEFM | 1.43E-07 | - |
| rs12593917 (FAM227B) | G | T | rs147273035 | G TA C C A | G | 0.92 | Adipose subcutaneous | FAM227B | 3.94E-07 | - |
|  |  |  | rs34729962 | A G | A | 1.00 | Neutrophils | GALK2 | 4.21E-10 | - |
|  |  |  | rs12050614 | C | T | 1.00 | Whole blood | GALK2 | 6.52E-12 | - |
|  |  |  | rs62021589 | T | G | 1.00 |  |  |  |  |
| rs1200503 (FCHO2) | C | G | rs258888 | A | T | 1.00 | Adipose subcutaneous | FCHO2 | 2.04E-24 | - |
|  |  |  | rs478575 | T | C | 1.00 | Adipose visceral omentum | FCHO2 | 4.68E-11 | - |
|  |  |  | rs30532 | C | T | 1.00 | Monocytes | FCHO2 | 1.57E-24 | - |
|  |  |  | rs194493 | T | G | 1.00 | Neutrophils | FCHO2 | 9.40E-35 | - |
|  |  |  |  |  |  |  | Spleen | FCHO2 | 1.52E-06 | - |
|  |  |  |  |  |  |  | T cells | FCHO2 | 1.79E-08 | - |
|  |  |  |  |  |  |  | Whole blood | FCHO2 | 3.27E-310 | - |
|  |  |  |  |  |  |  | Whole blood | TMEM171 | 1.72E-16 | + |
|  |  |  |  |  |  |  | Whole blood | TNPO1 | 1.85E-07 | + |
| rs3756772 (FRK) | T | C | rs3798233 | C | A | 0.99 | Whole blood | COL10A1 | 1.16E-08 | + |
|  |  |  | rs1890426 | C | T | 0.99 | Whole blood | DSE | 2.28E-06 | + |
|  |  |  | rs2351285 | T | C | 0.99 | Whole blood | FAM26F | 1.50E-08 | - |
|  |  |  | rs4946132 | T | C | 0.99 | Colon transverse | FRK | 7.69E-11 | + |
|  |  |  |  |  |  |  | Neutrophils | NT5DC1 | 1.90E-07 | + |
|  |  |  |  |  |  |  | Whole blood | NT5DC1 | 8.08E-31 | + |
|  |  |  |  |  |  |  | Whole blood | TSPYL1; DSE | 1.35E-06 | - |
| rs869202 (GAB2) | A | G | rs881338 | C | T | 1.00 | Liver | GAB2 | 1.97E-28 | - |
|  |  |  | rs881361 | C | G | 1.00 | T cells | GAB2 | 4.87E-10 | + |
|  |  |  | rs881337 | G | C | 1.00 | Whole blood | GAB2 | 2.49E-103 | - |
|  |  |  | rs11237489 | G | A | 1.00 | Whole blood | INTS4 | 1.16E-07 | + |

|  |  |  |  |  |  |  | Neutrophils | KCTD21 | 9.34E-06 | + |
| --- | --- | --- | --- | --- | --- | --- | --- | --- | --- | --- |
|  |  |  |  |  |  |  | Whole blood | KCTD21 | 2.11E-45 | + |
|  |  |  |  |  |  |  | Monocytes | NARS2 | 8.45E-17 | + |
|  |  |  |  |  |  |  | Neutrophils | NARS2 | 3.72E-09 | + |
|  |  |  |  |  |  |  | T cells | NARS2 | 5.63E-11 | + |
|  |  |  |  |  |  |  | Whole blood | NARS2 | 3.36E-205 | + |
|  |  |  |  |  |  |  | Whole blood | NDUFC2 | 1.45E-11 | + |
|  |  |  |  |  |  |  | Whole blood | RP11- 452H21.4 | 8.42E-68 | - |
|  |  |  |  |  |  |  | Whole blood | USP35 | 2.99E-127 | - |
| rs4485425 (GALK1, H3F3B) | A | G | rs67740167 | C | G | 0.98 | Whole blood | ACOX1 | 4.85E-69 | - |
|  |  |  | rs113500981 | C | C A | 0.98 | Whole blood | EVPL | 2.00E-12 | + |
|  |  |  | rs73352129 | G | C | 0.98 | Adipose subcutaneous | FBF1 | 2.82E-08 | + |
|  |  |  | rs8076052 | C | A | 0.98 | Adipose visceral omentum | FBF1 | 5.03E-09 | + |
|  |  |  |  |  |  |  | Colon sigmoid | FBF1 | 1.59E-08 | + |
|  |  |  |  |  |  |  | Colon transverse | FBF1 | 8.06E-09 | + |
|  |  |  |  |  |  |  | Whole blood | FBF1 | 1.02E-08 | - |
|  |  |  |  |  |  |  | Liver | GALK1 | 4.16E-13 | + |
|  |  |  |  |  |  |  | Whole blood | GALK1 | 2.87E-120 | - |
|  |  |  |  |  |  |  | Whole blood | H3F3B | 1.99E-40 | - |
|  |  |  |  |  |  |  | Whole blood | MRPL38 | 1.21E-06 | - |
|  |  |  |  |  |  |  | Whole blood | RP11- 552F3.10 | 8.97E-19 | - |
|  |  |  |  |  |  |  | Whole blood | RP11-552F3.4 | 1.26E-07 | - |
|  |  |  |  |  |  |  | Whole blood | RP11-552F3.9 | 7.62E-26 | + |
|  |  |  |  |  |  |  | Whole blood | TEN1-CDK3 | 2.13E-23 | - |
|  |  |  |  |  |  |  | Whole blood | TRIM47 | 1.17E-10 | - |
|  |  |  |  |  |  |  | Whole blood | TRIM65 | 7.09E-07 | - |
|  |  |  |  |  |  |  | Neutrophils | UNC13D | 2.12E-14 | - |
|  |  |  |  |  |  |  | Whole blood | UNC13D | 3.06E-48 | - |
|  |  |  |  |  |  |  | Adipose subcutaneous | WBP2 | 8.24E-06 | + |
| rs11706136 (GLB1) | A | G | rs12633904 | G | C | 0.81 | Whole blood | CCR4 | 3.19E-46 | - |
|  |  |  | rs11708012 | A | G | 0.80 | Adipose subcutaneous | CRTAP | 7.21E-07 | + |

|  |  |  | rs11129543 | T | G | 0.81 | Monocytes | GLB1 | 2.71E-07 | + |
| --- | --- | --- | --- | --- | --- | --- | --- | --- | --- | --- |
|  |  |  | rs4423716 | G | A | 0.80 | Subcutaneous fat | GLB1 | 1.40E-06 | NA |
|  |  |  |  |  |  |  | Whole blood | GLB1 | 3.56E-38 | + |
|  |  |  |  |  |  |  | Whole blood | PDCD6IP | 3.46E-12 | - |
| rs1658972 (GLDC, KDM4C) | C | T | rs34932926 | C A | C | 0.98 | Whole blood | ERMP1 | 3.64E-06 | + |
|  |  |  | rs12685293 | G | A | 0.96 | Liver | GLDC | 2.61E-10 | + |
|  |  |  | rs820504 | G | A | 0.95 | Whole blood | RP11-390F4.3 | 1.14E-06 | + |
|  |  |  | rs820503 | C | A | 0.95 |  |  |  |  |
| rs28383223 (HLA-DRB1, HLA-DQA1) | C | T | rs28383221 | A | G | 0.98 | Whole blood | AGER | 2.56E-06 | - |
|  |  |  | rs28383230 | T | C | 0.98 | Whole blood | ATF6B | 1.44E-14 | + |
|  |  |  |  |  |  |  | Adipose subcutaneous | CYP21A1P | 9.24E-06 | - |
|  |  |  |  |  |  |  | Whole blood | CYP21A1P | 3.92E-32 | - |
|  |  |  |  |  |  |  | Whole blood | CYP21A2 | 1.61E-06 | + |
|  |  |  |  |  |  |  | Whole blood | DDAH2 | 4.21E-30 | + |
|  |  |  |  |  |  |  | Whole blood | HLA-DOB | 5.68E-06 | - |
|  |  |  |  |  |  |  | Whole blood | HLA-DQA1 | 8.56E-35 | - |
|  |  |  |  |  |  |  | Adipose subcutaneous | HLA-DQA2 | 5.76E-18 | - |
|  |  |  |  |  |  |  | Adipose visceral omentum | HLA-DQA2 | 6.97E-11 | - |
|  |  |  |  |  |  |  | Colon sigmoid | HLA-DQA2 | 5.73E-06 | - |
|  |  |  |  |  |  |  | Colon transverse | HLA-DQA2 | 2.64E-09 | - |
|  |  |  |  |  |  |  | Spleen | HLA-DQA2 | 3.57E-06 | - |
|  |  |  |  |  |  |  | Whole blood | HLA-DQA2 | 1.94E-166 | - |
|  |  |  |  |  |  |  | Whole blood | HLA-DQB1 | 9.53E-13 | - |
|  |  |  |  |  |  |  | Whole blood | HLA-DQB1- AS1 | 1.09E-17 | + |
|  |  |  |  |  |  |  | Whole blood | HLA-DQB2 | 9.91E-06 | - |
|  |  |  |  |  |  |  | Adipose subcutaneous | HLA-DRB1 | 5.56E-08 | + |
|  |  |  |  |  |  |  | Adipose visceral omentum | HLA-DRB1 | 1.93E-06 | + |
|  |  |  |  |  |  |  | Spleen | HLA-DRB1 | 3.88E-06 | + |
|  |  |  |  |  |  |  | Whole blood | HLA-DRB1 | 4.77E-218 | + |
|  |  |  |  |  |  |  | Whole blood | HLA-DRB5 | 3.27E-310 | + |

|  |  |  |  |  |  |  | Adipose subcutaneous | HLA-DRB6 | 9.95E-35 | - |
| --- | --- | --- | --- | --- | --- | --- | --- | --- | --- | --- |
|  |  |  |  |  |  |  | Adipose visceral omentum | HLA-DRB6 | 5.74E-18 | - |
|  |  |  |  |  |  |  | Colon sigmoid | HLA-DRB6 | 2.75E-09 | - |
|  |  |  |  |  |  |  | Colon transverse | HLA-DRB6 | 2.71E-18 | - |
|  |  |  |  |  |  |  | Liver | HLA-DRB6 | 2.01E-08 | - |
|  |  |  |  |  |  |  | Small intestine terminal ileum | HLA-DRB6 | 2.07E-10 | - |
|  |  |  |  |  |  |  | Spleen | HLA-DRB6 | 1.90E-08 | - |
|  |  |  |  |  |  |  | Whole blood | HLA-DRB6 | 1.05E-160 | + |
|  |  |  |  |  |  |  | Whole blood | HSPA1B | 4.68E-11 | + |
|  |  |  |  |  |  |  | Whole blood | LY6G5B | 1.85E-26 | + |
|  |  |  |  |  |  |  | Whole blood | LY6G5C | 3.84E-39 | + |
|  |  |  |  |  |  |  | Adipose visceral omentum | PRRT1 | 3.00E-06 | - |
|  |  |  |  |  |  |  | Whole blood | PSMB9 | 7.54E-07 | + |
|  |  |  |  |  |  |  | Whole blood | SKIV2L | 2.89E-32 | - |
|  |  |  |  |  |  |  | Whole blood | STK19P | 2.27E-13 | - |
|  |  |  |  |  |  |  | Whole blood | TAP2 | 1.94E-18 | + |
|  |  |  |  |  |  |  | Whole blood | TNXA | 6.94E-12 | - |
| rs61352607 (INHBC) | G | T | rs2229357 | G | A | 1.00 | Whole blood | MARS | 4.90E-08 | + |
|  |  |  | rs3741414 | C | T | 1.00 | Whole blood | METTL21B | 6.21E-19 | - |
|  |  |  | rs73119306 | A | G | 0.99 | Whole blood | STAT6 | 2.03E-25 | + |
|  |  |  | rs12313306 | C | T | 0.97 | Whole blood | TMEM194A | 4.09E-18 | - |
|  |  |  |  |  |  |  | Whole blood | XRCC6BP1 | 5.20E-11 | + |
| rs12154248 (JAZF1) | C | T | rs4722763 | T | C | 1.00 | Whole blood | CREB5 | 2.11E-06 | + |
|  |  |  | rs12055996 | T | C | 1.00 | Liver | JAZF1 | 1.31E-06 | + |
|  |  |  | rs886664 | C | T | 1.00 | T cells | JAZF1 | 2.50E-11 | - |
|  |  |  | rs147809697 | G G T | G | 1.00 | Whole blood | JAZF1 | 6.18E-06 | - |
| rs717118 (KCNJ2, CASC17) | A | T | rs10221267 | T | C | 1.00 | Whole blood | KCNJ2 | 2.53E-11 | - |
|  |  |  | rs1911970 | C | T | 1.00 |  |  |  |  |
|  |  |  | rs4793370 | C | T | 1.00 |  |  |  |  |
|  |  |  | rs4519367 | G | A | 0.98 |  |  |  |  |

| rs11170319 (KRT78, KRT8) | G | T | rs5019799 | C | T | 0.89 | Whole blood | ITGB7 | 1.74E-12 | - |
| --- | --- | --- | --- | --- | --- | --- | --- | --- | --- | --- |
|  |  |  | rs5019800 | C | G | 0.89 | Whole blood | KRT1 | 2.94E-47 | - |
|  |  |  | rs4403881 | A | G | 0.89 | Whole blood | KRT72 | 1.18E-50 | + |
|  |  |  | rs4351899 | G | T | 0.89 | Whole blood | KRT73 | 9.01E-52 | + |
|  |  |  |  |  |  |  | Whole blood | KRT77 | 2.13E-08 | + |
|  |  |  |  |  |  |  | Whole blood | KRT79 | 3.40E-31 | - |
|  |  |  |  |  |  |  | Whole blood | KRT8 | 1.09E-10 | - |
|  |  |  |  |  |  |  | Whole blood | MFSD5 | 1.68E-07 | + |
|  |  |  |  |  |  |  | Whole blood | SOAT2 | 6.10E-06 | + |
| rs7740188 (L3MBTL3) | A | G | rs11390217 | TA | T | 1.00 | Adipose subcutaneous | L3MBTL3 | 3.00E-15 | - |
|  |  |  | rs55941023 | C T | C | 0.98 | Adipose visceral omentum | L3MBTL3 | 4.79E-13 | - |
|  |  |  | rs6926186 | A | G | 1.00 | Colon transverse | L3MBTL3 | 5.26E-17 | - |
|  |  |  | rs1415700 | A | G | 0.98 | Liver | L3MBTL3 | 2.51E-08 | - |
|  |  |  |  |  |  |  | Monocytes | L3MBTL3 | 3.26E-16 | - |
|  |  |  |  |  |  |  | Neutrophils | L3MBTL3 | 2.17E-16 | - |
|  |  |  |  |  |  |  | Small intestine terminal ileum | L3MBTL3 | 2.65E-10 | - |
|  |  |  |  |  |  |  | Spleen | L3MBTL3 | 4.88E-19 | - |
|  |  |  |  |  |  |  | T cells | L3MBTL3 | 2.46E-22 | - |
|  |  |  |  |  |  |  | Whole blood | L3MBTL3 | 3.27E-310 | - |
|  |  |  |  |  |  |  | Whole blood | SAMD3 | 2.99E-22 | - |
| rs2832277 (LINC00189) | A | G | rs2832264 | G | A | 0.98 | Whole blood | AF124730.4 | 2.81E-08 | - |
|  |  |  | rs8131557 | T | A | 0.99 | Whole blood | AF129075.5 | 6.59E-07 | + |
|  |  |  | rs1882862 | C | T | 0.98 | Whole blood | BACH1 | 1.72E-11 | - |
|  |  |  | rs2832265 | A | G | 0.98 | Whole blood | C21orf7 | 8.00E-20 | - |
|  |  |  |  |  |  |  | Neutrophils | CCT8 | 1.97E-09 | + |
|  |  |  |  |  |  |  | Monocytes | GAPDHP14 | 1.44E-07 | + |
|  |  |  |  |  |  |  | Neutrophils | GAPDHP14 | 1.14E-08 | + |
|  |  |  |  |  |  |  | Whole blood | GAPDHP14 | 3.74E-96 | + |
|  |  |  |  |  |  |  | Whole blood | GRIK1 | 1.33E-09 | + |
|  |  |  |  |  |  |  | Adipose subcutaneous | LINC00189 | 4.23E-11 | + |
|  |  |  |  |  |  |  | Adipose visceral omentum | LINC00189 | 1.44E-09 | + |

|  |  |  |  |  |  |  | Colon sigmoid | LINC00189 | 2.44E-06 | + |
| --- | --- | --- | --- | --- | --- | --- | --- | --- | --- | --- |
|  |  |  |  |  |  |  | Monocytes | LINC00189 | 1.02E-07 | + |
|  |  |  |  |  |  |  | Neutrophils | LINC00189 | 1.69E-09 | + |
|  |  |  |  |  |  |  | Whole blood | LINC00189 | 6.12E-219 | + |
|  |  |  |  |  |  |  | Whole blood | N6AMT1 | 1.61E-25 | + |
|  |  |  |  |  |  |  | Whole blood | RWDD2B | 2.29E-57 | + |
|  |  |  |  |  |  |  | Monocytes | RWDD2B | 1.70E-11 | + |
|  |  |  |  |  |  |  | T cells | RWDD2B | 1.23E-06 | + |
|  |  |  |  |  |  |  | Whole blood | RWDD2B | 2.47E-148 | + |
|  |  |  |  |  |  |  | Monocytes | ZNF294 | 2.20E-20 | NA |
| rs17046767 (LINC01191) | C | G | rs11695436 | C | T | 0.77 | Whole blood | AC010982.1 | 3.27E-310 | - |
|  |  |  | rs13011246 | G | A | 0.71 | Whole blood | AC016683.6 | 5.63E-23 | + |
|  |  |  | rs75771460 | G | C | 0.77 | Whole blood | AC017074.2 | 6.87E-08 | - |
|  |  |  | rs13023671 | G | A | 0.97 | Whole blood | AC104653.1 | 4.90E-28 | - |
|  |  |  |  |  |  |  | Whole blood | AC110769.3 | 2.17E-11 | - |
|  |  |  |  |  |  |  | Liver | ACTR3 | 1.11E-14 | NA |
|  |  |  |  |  |  |  | Whole blood | DDX11L2 | 9.79E-58 | - |
|  |  |  |  |  |  |  | Whole blood | LOC654433; PAX8 | 2.42E-06 | + |
|  |  |  |  |  |  |  | Whole blood | PAX8 | 1.79E-17 | + |
|  |  |  |  |  |  |  | Whole blood | RP11- 395L14.13 | 6.65E-07 | - |
|  |  |  |  |  |  |  | Adipose subcutaneous | RPL23AP7 | 3.29E-06 | - |
|  |  |  |  |  |  |  | Whole blood | RPL23AP7 | 1.48E-65 | - |
|  |  |  |  |  |  |  | Whole blood | SLC35F5 | 5.43E-15 | + |
|  |  |  |  |  |  |  | Whole blood | U3 | 2.57E-08 | - |
|  |  |  |  |  |  |  | Whole blood | WASH2P | 6.09E-16 | + |
| rs11086005 (LINC01835) | G | T | rs11086004 | A | G | 0.97 | Whole blood | AC004791.2 | 3.27E-310 | + |
|  |  |  | rs2079291 | A | G | 0.94 | Whole blood | CYP4F12 | 9.75E-14 | - |
|  |  |  | rs12462501 | A | G | 0.86 | Whole blood | CYP4F2 | 9.71E-07 | - |
|  |  |  | rs12462976 | T | G | 0.86 |  |  |  |  |
| rs2910953 (LINC02104, LINC00603) | A | T |  |  |  |  | Whole blood | DAB2 | 1.80E-16 | - |
| rs10858828 (LINC02458, DUSP6) | C | G | rs10431420 | C | A | 1.00 | Neutrophils | DUSP6 | 9.52E-09 | + |

|  |  |  | rs10745495 | G | C | 1.00 | Whole blood | DUSP6 | 1.83E-140 | + |
| --- | --- | --- | --- | --- | --- | --- | --- | --- | --- | --- |
|  |  |  | rs10431419 | A | C | 1.00 | Whole blood | GALNT4 | 3.01E-09 | + |
|  |  |  | rs10858830 | A | G | 1.00 | Whole blood | RP11-13A1.1 | 4.03E-13 | - |
|  |  |  |  |  |  |  | Whole blood | RP11-13A1.3 | 1.43E-06 | - |
| rs1800759 (LOC100507053) | T | G | rs201267712 | G | A | 0.92 | Whole blood | ACRBP | 7.52E-06 | - |
|  |  |  | rs2602836 | A | G | 0.92 | Whole blood | ADH1A; RP11- 696N14.1 | 2.57E-22 | - |
|  |  |  | rs2602844 | C | T | 0.92 | Adipose visceral omentum | ADH4 | 3.17E-06 | - |
|  |  |  | rs1133483 | G | C | 0.92 | Colon transverse | ADH4 | 1.71E-11 | + |
|  |  |  |  |  |  |  | Small intestine terminal ileum | ADH4 | 4.15E-06 | + |
|  |  |  |  |  |  |  | Spleen | ADH4 | 8.20E-13 | + |
|  |  |  |  |  |  |  | Dendritic cells | ADH5 | 1.57E-06 | NA |
|  |  |  |  |  |  |  | Monocytes | ADH5 | 1.31E-22 | + |
|  |  |  |  |  |  |  | Neutrophils | ADH5 | 9.58E-12 | + |
|  |  |  |  |  |  |  | Whole blood | ADH5 | 4.58E-39 | + |
|  |  |  |  |  |  |  | T cells | ADH6 | 1.87E-10 | - |
|  |  |  |  |  |  |  | Whole blood | ADH6 | 2.59E-26 | - |
|  |  |  |  |  |  |  | Whole blood | EIF4E | 1.72E-48 | - |
|  |  |  |  |  |  |  | Whole blood | RP11- 696N14.1 | 6.85E-36 | - |
|  |  |  |  |  |  |  | Whole blood | TSC22D1 | 1.20E-06 | - |
| rs4841436 (LOC102723313) | A | C | rs2898251 | C | G | 0.96 | Whole blood | AF131215.9 | 1.06E-120 | - |
|  |  |  | rs4840513 | T | G | 0.95 | Whole blood | BLK | 3.98E-84 | + |
|  |  |  | rs6601513 | C | T | 1.00 | Monocytes | CLDN23 | 1.54E-15 | NA |
|  |  |  | rs2116094 | T | C | 0.95 | Whole blood | CTSB | 1.40E-59 | - |
|  |  |  |  |  |  |  | Whole blood | ERI1 | 6.20E-20 | - |
|  |  |  |  |  |  |  | Whole blood | FAM167A | 2.32E-129 | - |
|  |  |  |  |  |  |  | Peripheral blood monocytes | FDFT1 | 1.05E-06 | NA |
|  |  |  |  |  |  |  | Whole blood | MSRA | 1.24E-95 | + |
|  |  |  |  |  |  |  | Whole blood | RP11- 148O21.2 | 1.40E-06 | + |
|  |  |  |  |  |  |  | Whole blood | RP11- 148O21.4 | 2.47E-25 | + |

|  |  |  |  |  |  |  | Whole blood | RP11-981G7.2 | 1.51E-09 | - |
| --- | --- | --- | --- | --- | --- | --- | --- | --- | --- | --- |
|  |  |  |  |  |  |  | Adipose subcutaneous | RP1L1 | 9.46E-06 | - |
|  |  |  |  |  |  |  | Whole blood | RP1L1 | 4.28E-20 | - |
|  |  |  |  |  |  |  | T cells | SLC35G5 | 3.55E-06 | + |
|  |  |  |  |  |  |  | Whole blood | SLC35G5 | 1.59E-16 | + |
|  |  |  |  |  |  |  | Whole blood | SOX7 | 1.45E-08 | - |
|  |  |  |  |  |  |  | Whole blood | XKR6 | 7.78E-09 | - |
| rs7189522 (LOC102724084) | C | T | rs28650012 | C | G | 0.99 | Whole blood | AGXT2L2 | 3.70E-06 | - |
|  |  |  | rs4581712 | C | A | 0.89 |  |  |  |  |
| rs6577596 (LOC105376975, LOC339862) | C | T | rs67851870 | G | A | 0.73 | Whole blood | TBC1D5 | 4.28E-07 | - |
|  |  |  | rs9870741 | G | T | 0.69 |  |  |  |  |
|  |  |  | rs11720653 | C | T | 0.69 |  |  |  |  |
|  |  |  | rs13321297 | A | T | 0.69 |  |  |  |  |
| rs2126259 (LOC157273) | T | C | rs6601299 | T | C | 0.99 | Whole blood | CTSB | 1.74E-15 | + |
|  |  |  | rs1461729 | A | G | 0.96 | Whole blood | LATS1 | 8.86E-07 | + |
|  |  |  | rs11781511 | A | G | 0.94 | Whole blood | MFHAS1 | 4.47E-14 | + |
|  |  |  | rs76453951 | T | C | 0.88 | Whole blood | RP11-10A14.4 | 4.96E-06 | + |
|  |  |  |  |  |  |  | Whole blood | RP11-10A14.5 | 5.91E-16 | + |
|  |  |  |  |  |  |  | Whole blood | RP11-62H7.2 | 1.38E-30 | + |
|  |  |  |  |  |  |  | Whole blood | SGK223 | 9.05E-06 | + |
| rs2943654 (LOC646736, MIR5702) | C | T | rs2972156 | C | G | 1.00 | Whole blood | CDHR4 | 6.48E-06 | - |
|  |  |  | rs2972138 | A | G | 0.99 | Adipose subcutaneous | IRS1 | 1.75E-11 | + |
|  |  |  | rs2972135 | G | T | 0.99 | Adipose visceral omentum | IRS1 | 5.96E-07 | + |
|  |  |  | rs2972146 | G | T | 0.98 | Whole blood | PPP2R2D | 2.73E-06 | - |
| rs10849448 (LTBR) | A | G | rs2364481 | A | G | 0.92 | Whole blood | ABLIM1 | 6.36E-06 | - |
|  |  |  | rs2364480 | C | A | 0.93 | Whole blood | ACTN1 | 2.98E-06 | - |
|  |  |  | rs11064157 | A | C | 0.88 | Whole blood | ANTXR2 | 6.67E-07 | + |
|  |  |  | rs4301834 | G | A | 0.87 | Whole blood | C1QB | 9.10E-06 | + |
|  |  |  |  |  |  |  | Whole blood | CACHD1 | 1.72E-06 | - |
|  |  |  |  |  |  |  | Whole blood | CALHM2 | 4.72E-07 | + |
|  |  |  |  |  |  |  | Whole blood | CAMK2D | 1.49E-07 | - |

| Whole blood | CCR7 | 2.59E-06 | - |
| --- | --- | --- | --- |
| Whole blood | CD248 | 7.55E-08 | - |
| Whole blood | CD27 | 7.10E-08 | + |
| Whole blood | CD27-AS1 | 1.06E-19 | - |
| Whole blood | CLDND1 | 1.00E-06 | + |
| Whole blood | CLEC4F | 4.67E-08 | + |
| Whole blood | CSF1R | 6.93E-06 | + |
| Whole blood | DBNDD1 | 6.58E-06 | - |
| Whole blood | DUSP5 | 7.16E-13 | + |
| Whole blood | EDAR | 3.66E-06 | - |
| Whole blood | FHIT | 2.89E-06 | - |
| Whole blood | GPBAR1 | 6.44E-09 | + |
| Whole blood | GPR183 | 2.68E-07 | + |
| Whole blood | GSN | 3.24E-06 | - |
| Whole blood | HMOX1 | 1.09E-07 | + |
| Whole blood | IFFO1 | 1.37E-10 | + |
| Whole blood | ITPRIPL2 | 8.05E-06 | + |
| Whole blood | KIF5C | 6.52E-09 | + |
| Whole blood | LPAR5 | 2.26E-06 | + |
| Adipose subcutaneous | LTBR | 4.39E-69 | + |
| Adipose visceral omentum | LTBR | 5.62E-41 | + |
| Colon sigmoid | LTBR | 7.43E-16 | + |
| Colon transverse | LTBR | 3.31E-29 | + |
| Dendritic cells | LTBR | 2.63E-08 | NA |
| Liver | LTBR | 2.46E-08 | + |
| Monocytes | LTBR | 6.97E-15 | + |
| Neutrophils | LTBR | 2.66E-06 | - |
| Small intestine terminal ileum | LTBR | 7.76E-06 | + |
| Spleen | LTBR | 4.53E-06 | + |
| Subcutaneous fat | LTBR | 1.57E-62 | NA |
| Visceral abdominal fat | LTBR | 5.85E-53 | NA |
| Whole blood | LTBR | 1.08E-205 | + |

|  |  |  |  |  |  |  | Whole blood | MAP3K4 | 9.01E-06 | + |
| --- | --- | --- | --- | --- | --- | --- | --- | --- | --- | --- |
|  |  |  |  |  |  |  | Whole blood | MMP28 | 7.20E-10 | - |
|  |  |  |  |  |  |  | Whole blood | MS4A7 | 4.29E-12 | + |
|  |  |  |  |  |  |  | Whole blood | PAPSS2 | 6.76E-06 | + |
|  |  |  |  |  |  |  | Whole blood | PEA15 | 2.70E-08 | - |
|  |  |  |  |  |  |  | Whole blood | PHGDH | 5.59E-06 | - |
|  |  |  |  |  |  |  | Whole blood | PQLC3 | 9.10E-06 | + |
|  |  |  |  |  |  |  | Whole blood | RP1-102E24.8 | 1.77E-22 | - |
|  |  |  |  |  |  |  | Whole blood | SIDT2 | 2.10E-06 | + |
|  |  |  |  |  |  |  | Whole blood | SLC20A1 | 7.33E-06 | - |
|  |  |  |  |  |  |  | Whole blood | SLC38A11 | 2.99E-06 | - |
|  |  |  |  |  |  |  | Whole blood | SLC7A7 | 2.46E-06 | + |
|  |  |  |  |  |  |  | Whole blood | TAPBPL | 3.73E-09 | - |
|  |  |  |  |  |  |  | Whole blood | TNFRSF4 | 1.13E-07 | + |
|  |  |  |  |  |  |  | Whole blood | TPPP3 | 4.90E-07 | + |
|  |  |  |  |  |  |  | Whole blood | TRABD2A | 8.03E-06 | - |
|  |  |  |  |  |  |  | Whole blood | TTC39C | 5.25E-07 | + |
|  |  |  |  |  |  |  | Whole blood | TXK | 3.42E-06 | - |
|  |  |  |  |  |  |  | Whole blood | VAMP1 | 1.25E-09 | + |
|  |  |  |  |  |  |  | Whole blood | YWHAH | 2.58E-06 | + |
| rs6034011 (MACROD2) | C | T | rs1932951 | T | C | 0.89 | Whole blood | NDUFAF5 | 4.95E-06 | - |
|  |  |  | rs6135228 | A | G | 0.89 |  |  |  |  |
| rs4821764 (MAFF) | A | G | rs4820323 | G | C | 1.00 | Whole blood | ANKRD54 | 6.29E-12 | + |
|  |  |  | rs4820325 | A | G | 1.00 | Adipose subcutaneous | BAIAP2L2 | 3.85E-07 | + |
|  |  |  | rs2267373 | T | C | 1.00 | Whole blood | CSNK1E | 1.82E-30 | + |
|  |  |  | rs2267375 | T | G | 1.00 | Whole blood | H1F0 | 2.36E-06 | + |
|  |  |  |  |  |  |  | Whole blood | LGALS2 | 2.29E-06 | - |
|  |  |  |  |  |  |  | Adipose subcutaneous | MAFF | 4.09E-10 | + |
|  |  |  |  |  |  |  | Monocytes | MAFF | 7.07E-10 | - |
|  |  |  |  |  |  |  | Neutrophils | MAFF | 9.45E-06 | - |
|  |  |  |  |  |  |  | Whole blood | MAFF | 3.76E-61 | - |
|  |  |  |  |  |  |  | Whole blood | NOL12 | 1.80E-13 | + |
|  |  |  |  |  |  |  | Whole blood | PICK1 | 1.10E-12 | + |

|  |  |  |  |  |  |  | T cells | PLA2G6 | 5.51E-14 | - |
| --- | --- | --- | --- | --- | --- | --- | --- | --- | --- | --- |
|  |  |  |  |  |  |  | Whole blood | PLA2G6 | 1.28E-45 | - |
|  |  |  |  |  |  |  | Whole blood | RP1-5O6.6 | 6.88E-13 | + |
|  |  |  |  |  |  |  | Whole blood | SH3BP1 | 8.33E-07 | + |
|  |  |  |  |  |  |  | Whole blood | TMEM184B | 1.58E-103 | + |
|  |  |  |  |  |  |  | Colon sigmoid | TOMM22 | 7.45E-06 | + |
| rs12544992 (MFHAS1) | C | G | rs11784052 | C | T | 0.96 | Adipose visceral omentum | ALG1L13P | 3.05E-06 | - |
|  |  |  | rs7823757 | T | A | 0.96 | T cells | ALG1L13P | 5.34E-11 | + |
|  |  |  | rs35039922 | A | T | 0.96 | Whole blood | ALG1L13P | 2.38E-47 | - |
|  |  |  | rs60315134 | A | G | 0.96 | Whole blood | BLK | 4.40E-14 | + |
|  |  |  |  |  |  |  | Adipose subcutaneous | CLDN23 | 2.29E-08 | - |
|  |  |  |  |  |  |  | Whole blood | CLDN23 | 5.97E-08 | - |
|  |  |  |  |  |  |  | Whole blood | CTSB | 1.08E-111 | - |
|  |  |  |  |  |  |  | Neutrophils | ERI1 | 4.90E-07 | - |
|  |  |  |  |  |  |  | Whole blood | ERI1 | 9.37E-35 | - |
|  |  |  |  |  |  |  | Adipose subcutaneous | FAM85B | 4.87E-10 | + |
|  |  |  |  |  |  |  | Adipose visceral omentum | FAM85B | 6.01E-07 | + |
|  |  |  |  |  |  |  | Monocytes | FAM85B | 4.89E-08 | + |
|  |  |  |  |  |  |  | Neutrophils | FAM85B | 1.63E-17 | + |
|  |  |  |  |  |  |  | Spleen | FAM85B | 1.50E-06 | + |
|  |  |  |  |  |  |  | Adipose subcutaneous | FAM86B3P | 3.92E-06 | - |
|  |  |  |  |  |  |  | Adipose visceral omentum | FAM86B3P | 5.47E-18 | - |
|  |  |  |  |  |  |  | Colon transverse | FAM86B3P | 8.11E-11 | - |
|  |  |  |  |  |  |  | T cells | FAM86B3P | 1.37E-17 | + |
|  |  |  |  |  |  |  | Whole blood | FAM86B3P | 1.76E-17 | + |
|  |  |  |  |  |  |  | Monocytes | MFHAS1 | 1.46E-08 | - |
|  |  |  |  |  |  |  | Whole blood | MFHAS1 | 9.35E-113 | - |
|  |  |  |  |  |  |  | Whole blood | MSRA | 5.44E-06 | + |
|  |  |  |  |  |  |  | Whole blood | MTMR9 | 4.96E-08 | - |
|  |  |  |  |  |  |  | Whole blood | RP11-10A14.3 | 2.05E-17 | - |
|  |  |  |  |  |  |  | Whole blood | RP11-10A14.5 | 6.86E-118 | - |
|  |  |  |  |  |  |  | Whole blood | RP11-62H7.2 | 3.39E-179 | - |

|  |  |  |  |  |  |  | Whole blood | SGK223 | 7.24E-25 | - |
| --- | --- | --- | --- | --- | --- | --- | --- | --- | --- | --- |
|  |  |  |  |  |  |  | Whole blood | XKR6 | 2.57E-11 | - |
| rs6589941 (MIR100HG, UBASH3B) | G | T | rs10892870 | G | A | 0.99 | Adipose subcutaneous | UBASH3B | 3.96E-06 | + |
|  |  |  | rs6589940 | G | A | 0.99 | Neutrophils | UBASH3B | 2.16E-12 | - |
|  |  |  | rs7124914 | C | T | 0.99 | Whole blood | UBASH3B | 2.07E-18 | - |
|  |  |  | rs1945390 | C | T | 0.96 |  |  |  |  |
| rs6916318 (MIR588, RSPO3) | A | T | rs9482771 | G | C | 0.86 | Whole blood | RNF146 | 2.82E-18 | + |
|  |  |  | rs1936800 | C | T | 0.95 | Whole blood | RP11-90D4.2 | 7.48E-06 | - |
|  |  |  | rs1936801 | A | G | 0.95 | Adipose subcutaneous | RSPO3 | 4.66E-12 | - |
|  |  |  | rs9491696 | C | G | 0.86 |  |  |  |  |
| rs17710008 (MYCT1) | A | G | rs73005239 | A | G | 0.97 | Whole blood | MYCT1; HSPD1 | 3.90E-25 | - |
|  |  |  | rs73003601 | G | A | 0.97 | Whole blood | SYNE1 | 2.09E-09 | + |
|  |  |  | rs2078355 | G | A | 0.66 | Monocytes | SYNE1 | 3.36E-07 | + |
|  |  |  | rs142952476 | A | G | 0.96 | Whole blood | TRPS1 | 7.29E-07 | + |
| rs4782568 (MLYCD, OSGIN1) | C | G | rs12933677 | T | C | 0.93 | Whole blood | MLYCD | 3.54E-15 | - |
|  |  |  | rs11149612 | C | T | 0.98 | Whole blood | NECAB2 | 4.67E-08 | - |
|  |  |  | rs2255540 | A | G | 0.80 | Liver | OSGIN1 | 5.76E-06 | NA |
|  |  |  | rs67890964 | T | C | 0.77 | Neutrophils | OSGIN1 | 1.71E-17 | - |
|  |  |  |  |  |  |  | Whole blood | OSGIN1 | 3.07E-101 | - |
|  |  |  |  |  |  |  | Whole blood | RP11- 483P21.6 | 6.69E-06 | - |
| rs7209484 (NFE2L1, CBX1) | C | T | rs8438 | A | G | 0.96 | Whole blood | CBX1 | 7.46E-21 | - |
|  |  |  | rs62064917 | A | G | 0.96 | Whole blood | CDK5RAP3 | 9.50E-08 | + |
|  |  |  | rs8081209 | G | A | 0.96 | Whole blood | COPZ2 | 1.54E-27 | + |
|  |  |  |  |  |  |  | Monocytes | EFCAB13 | 1.75E-06 | + |
|  |  |  |  |  |  |  | Adipose subcutaneous | HOXB2 | 2.91E-07 | - |
|  |  |  |  |  |  |  | T cells | HOXB2 | 1.40E-06 | - |
|  |  |  |  |  |  |  | Whole blood | HOXB2 | 3.27E-310 | - |
|  |  |  |  |  |  |  | Whole blood | HOXB3 | 1.85E-26 | - |
|  |  |  |  |  |  |  | Whole blood | HOXB4 | 1.00E-31 | - |
|  |  |  |  |  |  |  | Adipose subcutaneous | HOXB5 | 3.12E-07 | - |
|  |  |  |  |  |  |  | Whole blood | HOXB7 | 2.24E-07 | - |

|  |  |  |  |  |  |  | T cells | HOXB-AS1 | 6.19E-06 | - |
| --- | --- | --- | --- | --- | --- | --- | --- | --- | --- | --- |
|  |  |  |  |  |  |  | Whole blood | HOXB-AS1 | 1.45E-45 | - |
|  |  |  |  |  |  |  | Whole blood | LRRC46 | 5.13E-10 | - |
|  |  |  |  |  |  |  | Monocytes | NFE2L1 | 8.40E-06 | - |
|  |  |  |  |  |  |  | Whole blood | NFE2L1 | 1.81E-29 | - |
|  |  |  |  |  |  |  | Whole blood | PNPO | 9.63E-52 | + |
|  |  |  |  |  |  |  | Whole blood | RP11-6N17.4 | 1.56E-56 | - |
|  |  |  |  |  |  |  | Whole blood | SCRN2 | 1.29E-70 | - |
|  |  |  |  |  |  |  | Whole blood | SKAP1 | 2.58E-134 | - |
|  |  |  |  |  |  |  | Colon transverse | SNX11 | 7.86E-06 | + |
|  |  |  |  |  |  |  | Small intestine terminal ileum | SNX11 | 3.42E-06 | + |
|  |  |  |  |  |  |  | Whole blood | SNX11 | 2.74E-17 | + |
|  |  |  |  |  |  |  | Monocytes | TBKBP1 | 3.20E-07 | + |
|  |  |  |  |  |  |  | Whole blood | TBKBP1 | 2.94E-42 | + |
| rs72823014 (NHLRC2, ADRB1) | A | G | rs137967504 | A | AT | 0.90 | Monocytes | ADRB1 | 4.04E-30 | + |
|  |  |  | rs72823013 | A | G | 0.90 | Spleen | ADRB1 | 2.91E-08 | + |
|  |  |  | rs72823015 | T | G | 0.90 | Whole blood | ADRB1 | 7.08E-130 | + |
|  |  |  | rs72823016 | C | T | 0.90 | Whole blood | CASP7 | 4.68E-09 | + |
|  |  |  |  |  |  |  | Whole blood | NHLRC2 | 4.11E-14 | + |
| rs7018885 (NINJ1) | C | T |  |  |  |  | Whole blood | BICD2 | 8.84E-08 | + |
|  |  |  |  |  |  |  | Whole blood | C9orf89 | 6.12E-62 | - |
|  |  |  |  |  |  |  | Whole blood | NINJ1 | 2.38E-37 | - |
|  |  |  |  |  |  |  | Whole blood | SUSD3 | 1.89E-08 | - |
|  |  |  |  |  |  |  | Whole blood | WNK2 | 7.39E-10 | - |
| rs2289125 (NOX4) | C | A | rs2289123 | T | G | 0.91 | Whole blood | IER3IP1 | 7.58E-06 | + |
|  |  |  | rs3816123 | T | G | 0.77 | Whole blood | TRIM64B | 1.88E-06 | - |
| rs6747874 (NPAS2) | A | G | rs876060 | T | A | 0.88 | Whole blood | RPL31 | 1.13E-18 | + |
|  |  |  | rs6747755 | A | G | 1.00 | Whole blood | RPL31; TBC1D8 | 9.85E-27 | + |
|  |  |  | rs12622050 | A | G | 0.94 |  |  |  |  |
|  |  |  | rs12619710 | C | T | 0.90 |  |  |  |  |
| rs7117339 (PANX1) | C | T | rs11607757 | T | G | 1.00 | Whole blood | C11orf75 | 5.17E-29 | - |

|  |  |  | rs56175344 | C | G | 0.98 | Whole blood | GPR83 | 3.23E-06 | - |
| --- | --- | --- | --- | --- | --- | --- | --- | --- | --- | --- |
|  |  |  | rs11604580 | G | A | 0.93 | Adipose subcutaneous | HEPHL1 | 1.29E-08 | - |
|  |  |  | rs4342991 | G | C | 0.93 | Colon sigmoid | HEPHL1 | 2.15E-11 | - |
|  |  |  |  |  |  |  | Colon transverse | HEPHL1 | 3.68E-10 | - |
|  |  |  |  |  |  |  | Whole blood | HEPHL1 | 5.91E-24 | - |
|  |  |  |  |  |  |  | Whole blood | MRE11A | 1.37E-27 | + |
|  |  |  |  |  |  |  | Adipose subcutaneous | PANX1 | 3.53E-08 | + |
|  |  |  |  |  |  |  | Liver | PANX1 | 1.23E-06 | + |
|  |  |  |  |  |  |  | Monocytes | PANX1 | 2.11E-07 | + |
|  |  |  |  |  |  |  | Visceral abdominal fat | PANX1 | 2.10E-13 | NA |
|  |  |  |  |  |  |  | Whole blood | PANX1 | 5.60E-225 | + |
|  |  |  |  |  |  |  | Whole blood | RP11- 685N10.1 | 1.31E-13 | + |
| rs7256564 (PEPD) | A | G | rs8109191 | T | G | 0.95 | Whole blood | CEBPA | 2.64E-18 | - |
|  |  |  | rs4805878 | T | C | 0.95 | Whole blood | LRP3 | 2.93E-06 | - |
|  |  |  | rs8106165 | A | G | 0.95 | Whole blood | PEPD | 1.29E-15 | - |
|  |  |  | rs7250869 | T | C | 0.95 |  |  |  |  |
| rs45587331 (PIK3AP1) | A | G | rs61856816 | C | T | 0.80 | Whole blood | GPR18 | 5.54E-06 | - |
|  |  |  | rs12573330 | C | A | 0.78 | Whole blood | PIK3AP1 | 8.73E-26 | - |
|  |  |  | rs61856817 | T | C | 0.80 |  |  |  |  |
|  |  |  | rs17112076 | T | C | 0.78 |  |  |  |  |
| rs1002436 (PKN2-AS1) | G | A | rs10922478 | G | A | 1.00 | Adipose subcutaneous | CCBL2 | 4.98E-15 | + |
|  |  |  | rs1565497 | C | G | 0.97 | Adipose visceral omentum | CCBL2 | 1.16E-13 | + |
|  |  |  |  |  |  |  | Colon sigmoid | CCBL2 | 2.42E-07 | + |
|  |  |  |  |  |  |  | Colon transverse | CCBL2 | 9.00E-12 | + |
|  |  |  |  |  |  |  | Neutrophils | CCBL2 | 3.84E-13 | + |
|  |  |  |  |  |  |  | Whole blood | CCBL2 | 1.72E-273 | + |
|  |  |  |  |  |  |  | Whole blood | GBP3 | 9.36E-44 | + |
|  |  |  |  |  |  |  | Whole blood | GTF2B | 6.69E-43 | - |
|  |  |  |  |  |  |  | Whole blood | PKN2 | 4.25E-08 | + |
|  |  |  |  |  |  |  | Whole blood | RBMXL1 | 9.58E-60 | + |
| rs738409 (PNPLA3) | G | C | rs738408 | T | C | 1.00 | Whole blood | FAM89B | 5.49E-06 | + |
|  |  |  | rs3747207 | A | G | 0.99 | Subcutaneous fat | PNPLA3 | 1.64E-06 | NA |

|  |  |  | rs2294915 | T | C | 0.94 | Adipose subcutaneous | SAMM50 | 4.68E-07 | + |
| --- | --- | --- | --- | --- | --- | --- | --- | --- | --- | --- |
|  |  |  | rs12485100 | T | G | 0.80 | Whole blood | SAMM50 | 1.51E-111 | + |
| rs4135247 (PPARG) | A | G | rs4518111 | C | A | 0.90 | Whole blood | PPARG | 2.50E-12 | - |
|  |  |  |  |  |  |  | Whole blood | MKRN2 | 5.87E-27 | + |
| rs313839 (PRKD2, STRN4) | C | G | rs402072 | T | C | 0.98 | Adipose subcutaneous | FKRP | 1.68E-14 | - |
|  |  |  | rs425105 | T | C | 0.86 | Adipose visceral omentum | FKRP | 1.44E-08 | - |
|  |  |  | rs60652743 | A | G | 0.85 | Colon sigmoid | FKRP | 2.15E-11 | - |
|  |  |  | rs113374757 | C | T | 0.81 | Colon transverse | FKRP | 8.81E-11 | - |
|  |  |  |  |  |  |  | Monocytes | FKRP | 3.26E-10 | - |
|  |  |  |  |  |  |  | Neutrophils | FKRP | 1.77E-11 | - |
|  |  |  |  |  |  |  | T cells | FKRP | 4.16E-10 | - |
|  |  |  |  |  |  |  | Whole blood | FKRP | 1.56E-251 | - |
|  |  |  |  |  |  |  | Whole blood | GNG8 | 6.78E-07 | - |
|  |  |  |  |  |  |  | Whole blood | MIR320E | 8.59E-44 | - |
|  |  |  |  |  |  |  | Adipose subcutaneous | PRKD2 | 3.09E-09 | - |
|  |  |  |  |  |  |  | Colon sigmoid | PRKD2 | 5.20E-09 | - |
|  |  |  |  |  |  |  | Colon transverse | PRKD2 | 7.49E-08 | - |
|  |  |  |  |  |  |  | Monocytes | PRKD2 | 3.08E-25 | - |
|  |  |  |  |  |  |  | Neutrophils | PRKD2 | 3.77E-19 | - |
|  |  |  |  |  |  |  | Whole blood | PRKD2 | 3.27E-310 | - |
|  |  |  |  |  |  |  | Whole blood | SLC1A5 | 1.22E-06 | + |
| rs2491441 (RGL1) | T | C | rs2500110 | C | T | 1.00 | Whole blood | ARPC5 | 3.44E-10 | - |
|  |  |  | rs2491440 | G | A | 1.00 | Whole blood | RGL1 | 3.18E-07 | + |
| rs10409243 (S1PR2) | C | T | rs2116942 | T | G | 0.91 | Whole blood | ANGPTL6 | 7.91E-06 | + |
|  |  |  |  |  |  |  | Whole blood | C19orf66 | 1.37E-25 | + |
|  |  |  |  |  |  |  | Liver | DNMT1 | 1.71E-12 | NA |
|  |  |  |  |  |  |  | Whole blood | DNMT1 | 3.35E-12 | - |
|  |  |  |  |  |  |  | Whole blood | EIF3G | 1.28E-16 | + |
|  |  |  |  |  |  |  | Whole blood | ICAM1 | 2.02E-08 | - |
|  |  |  |  |  |  |  | Whole blood | ICAM5 | 9.21E-11 | - |
|  |  |  |  |  |  |  | Adipose visceral omentum | S1PR2 | 6.37E-06 | - |
|  |  |  |  |  |  |  | Whole blood | S1PR2 | 7.79E-07 | + |

|  |  |  |  |  |  |  | Whole blood | SLC44A2 | 2.84E-06 | + |
| --- | --- | --- | --- | --- | --- | --- | --- | --- | --- | --- |
|  |  |  |  |  |  |  | Whole blood | ZNF266 | 8.84E-09 | - |
| rs61980636 (SERPINA6) | C | T | rs4905179 | A | G | 0.97 | Whole blood | SERPINA1 | 4.07E-36 | + |
|  |  |  | rs61280460 | A | T | 0.97 |  |  |  |  |
|  |  |  | rs1956178 | C | A | 0.97 |  |  |  |  |
| rs2727324 (SMARCD2, TCAM1P) | C | G | rs56966266 | A | AT | 0.90 | Neutrophils | CCDC47 | 1.33E-06 | + |
|  |  |  | rs4480868 | T | C | 0.93 | Whole blood | CCDC47 | 1.61E-34 | + |
|  |  |  |  |  |  |  | Adipose subcutaneous | CD79B | 5.24E-15 | - |
|  |  |  |  |  |  |  | Whole blood | CD79B | 3.87E-12 | + |
|  |  |  |  |  |  |  | Liver | DDX42 | 4.06E-06 | - |
|  |  |  |  |  |  |  | Whole blood | DDX42 | 1.81E-10 | - |
|  |  |  |  |  |  |  | Adipose subcutaneous | FTSJ3 | 2.54E-17 | + |
|  |  |  |  |  |  |  | Adipose visceral omentum | FTSJ3 | 4.71E-12 | + |
|  |  |  |  |  |  |  | Colon sigmoid | FTSJ3 | 5.71E-06 | + |
|  |  |  |  |  |  |  | Colon transverse | FTSJ3 | 2.00E-07 | + |
|  |  |  |  |  |  |  | Whole blood | FTSJ3 | 3.71E-37 | + |
|  |  |  |  |  |  |  | Whole blood | FTSJ3 | 8.24E-121 | + |
|  |  |  |  |  |  |  | Whole blood | ICAM2 | 1.06E-10 | + |
|  |  |  |  |  |  |  | Whole blood | LIMD2 | 1.16E-41 | + |
|  |  |  |  |  |  |  | Adipose subcutaneous | PSMC5 | 4.35E-10 | + |
|  |  |  |  |  |  |  | Adipose visceral omentum | PSMC5 | 3.12E-07 | + |
|  |  |  |  |  |  |  | Colon transverse | PSMC5 | 1.91E-08 | + |
|  |  |  |  |  |  |  | Whole blood | PSMC5 | 1.06E-43 | + |
|  |  |  |  |  |  |  | Whole blood | SMARCD2 | 8.73E-34 | - |
|  |  |  |  |  |  |  | Adipose subcutaneous | STRADA | 6.16E-09 | - |
|  |  |  |  |  |  |  | Adipose visceral omentum | STRADA | 1.12E-07 | - |
|  |  |  |  |  |  |  | Whole blood | STRADA | 3.61E-22 | - |
|  |  |  |  |  |  |  | Adipose subcutaneous | TCAM1P | 2.13E-06 | - |
|  |  |  |  |  |  |  | Adipose visceral omentum | TCAM1P | 2.79E-07 | - |
|  |  |  |  |  |  |  | Spleen | TCAM1P | 1.78E-06 | - |

|  |  |  |  |  |  |  | Whole blood | TCAM1P | 8.10E-07 | - |
| --- | --- | --- | --- | --- | --- | --- | --- | --- | --- | --- |
| rs1917368 (SNX13) | G | T | rs6461354 | C | T | 0.98 | Whole blood | C6orf164 | 6.42E-06 | - |
|  |  |  | rs10242866 | C | T | 0.96 | Adipose subcutaneous | SNX13 | 9.45E-07 | - |
|  |  |  | rs10282707 | C | T | 0.96 | Whole blood | SNX13 | 1.56E-06 | + |
|  |  |  | rs4142995 | G | T | 0.96 |  |  |  |  |
| rs1477066 (SOX9-AS1) | T | C |  |  |  |  | Whole blood | HES7 | 1.65E-06 | - |
| rs58504358 (ST3GAL1) | C | T | rs62518380 | A | G | 0.83 | Whole blood | HSPBP1 | 1.16E-06 | - |
|  |  |  | rs16904940 | T | C | 0.80 | Whole blood | LTA4H | 3.75E-06 | - |
|  |  |  | rs59413766 | A | C | 0.91 | Whole blood | ST3GAL1 | 2.07E-185 | - |
|  |  |  | rs62520290 | A | G | 0.85 |  |  |  |  |
| rs112771035 (ST3GAL4) | C | G | rs78471630 | G | T | 0.76 | Whole blood | DBI | 3.96E-06 | - |
|  |  |  | rs76970536 | G | A | 0.69 | Whole blood | DCPS | 2.09E-113 | + |
|  |  |  | rs73632737 | G | C | 0.61 | Whole blood | FAM118B | 8.33E-20 | + |
|  |  |  | rs376145133 | G G A G T G TA G G C A | G | 0.65 | Whole blood | FOXRED1 | 3.97E-07 | - |
|  |  |  |  |  |  |  | Whole blood | KIRREL3 | 4.15E-06 | - |
|  |  |  |  |  |  |  | Whole blood | RPUSD4 | 2.49E-63 | + |
|  |  |  |  |  |  |  | T cells | ST3GAL4 | 7.51E-06 | - |
|  |  |  |  |  |  |  | Whole blood | TES | 9.56E-06 | + |
|  |  |  |  |  |  |  | Whole blood | TIRAP | 1.67E-07 | - |
| rs12406530 (ST7L) | A | G | rs34136813 | C T G A G | C | 0.99 | Adipose visceral omentum | MOV10 | 5.66E-06 | - |
|  |  |  | rs3737136 | A | G | 0.99 | Neutrophils | MOV10 | 1.42E-09 | - |
|  |  |  | rs35147679 | C | T | 0.99 | T cells | MOV10 | 3.28E-06 | - |
|  |  |  | rs12117479 | T | A | 0.99 | Whole blood | MOV10 | 9.83E-127 | - |
|  |  |  |  |  |  |  | Whole blood | RHOC | 7.57E-08 | + |
|  |  |  |  |  |  |  | Adipose subcutaneous | ST7L | 4.14E-21 | - |

|  |  |  |  |  |  |  | Adipose visceral omentum | ST7L | 4.91E-13 | - |
| --- | --- | --- | --- | --- | --- | --- | --- | --- | --- | --- |
|  |  |  |  |  |  |  | Colon sigmoid | ST7L | 2.95E-07 | - |
|  |  |  |  |  |  |  | Colon transverse | ST7L | 3.92E-08 | - |
|  |  |  |  |  |  |  | Monocytes | ST7L | 1.53E-09 | - |
|  |  |  |  |  |  |  | Whole blood | ST7L | 3.27E-310 | - |
|  |  |  |  |  |  |  | T cells | WNT2B | 9.90E-08 | - |
| rs481206 (STARD10) | C | T | rs369319448 | T | T G G G G G C T C | 0.93 | Adipose subcutaneous | STARD10 | 2.01E-25 | - |
|  |  |  | rs663015 | T | C | 0.99 | Adipose visceral omentum | STARD10 | 1.59E-14 | - |
|  |  |  | rs519790 | C | G | 0.92 | Colon sigmoid | STARD10 | 1.80E-08 | - |
|  |  |  | rs12795307 | G | A | 0.89 | Liver | STARD10 | 2.21E-08 | - |
|  |  |  |  |  |  |  | Monocytes | STARD10 | 9.01E-20 | - |
|  |  |  |  |  |  |  | Neutrophils | STARD10 | 3.83E-29 | - |
|  |  |  |  |  |  |  | Spleen | STARD10 | 2.19E-09 | - |
|  |  |  |  |  |  |  | Subcutaneous fat | STARD10 | 1.79E-33 | NA |
|  |  |  |  |  |  |  | Visceral abdominal fat | STARD10 | 3.44E-38 | NA |
|  |  |  |  |  |  |  | Whole blood | ARAP1 | 4.89E-12 | - |
|  |  |  |  |  |  |  | Whole blood | ARAP1-AS2 | 3.43E-24 | - |
|  |  |  |  |  |  |  | Whole blood | ATG16L2 | 6.31E-07 | + |
|  |  |  |  |  |  |  | Whole blood | PDE2A | 2.25E-14 | - |
|  |  |  |  |  |  |  | Whole blood | RP11-169D4.2 | 7.08E-08 | + |
|  |  |  |  |  |  |  | Whole blood | STARD10 | 3.27E-310 | - |
| rs30386 (TBC1D9B) | T | G | rs378684 | C | T | 0.70 | Whole blood | C5orf45 | 1.12E-11 | - |
|  |  |  | rs416974 | G | C | 0.69 | Whole blood | CANX | 8.96E-10 | + |
|  |  |  | rs155791 | T | C | 0.73 | Whole blood | HNRNPH1 | 2.25E-12 | - |
|  |  |  |  |  |  |  | Whole blood | MGAT4B | 5.21E-24 | + |
|  |  |  |  |  |  |  | Whole blood | RNF130 | 3.16E-07 | - |
|  |  |  |  |  |  |  | Whole blood | RUFY1 | 5.02E-06 | - |

|  |  |  |  |  |  |  | CD4+ lymphocytes | SQSTM1 | 4.79E-06 | NA |
| --- | --- | --- | --- | --- | --- | --- | --- | --- | --- | --- |
|  |  |  |  |  |  |  | Whole blood | SQSTM1 | 8.54E-73 | + |
| rs2568207 (TCF7L1) | A | T | rs6547599 | A | T | 0.86 | Whole blood | CAPG | 1.25E-19 | - |
|  |  |  | rs2568211 | A | G | 0.82 | Whole blood | GNLY | 5.78E-10 | + |
|  |  |  | rs6709118 | G | A | 0.81 | Whole blood | KCNB2 | 2.19E-08 | + |
|  |  |  | rs59112392 | G | T | 0.81 | Whole blood | TGOLN2 | 3.21E-06 | + |
| rs58542926 (TM6SF2) | C | T | rs150641967 | T G A C A | T | 1.00 | Whole blood | ASPRV1 | 8.59E-06 | - |
|  |  |  | rs200210321 | A | A G | 1.00 | Whole blood | ATP10A | 2.40E-06 | + |
|  |  |  | rs56255430 | A | C | 0.87 | Adipose subcutaneous | ATP13A1 | 1.13E-06 | - |
|  |  |  | rs73002956 | A | G | 0.83 | Whole blood | ATP13A1 | 3.27E-310 | - |
|  |  |  |  |  |  |  | Whole blood | CXCL16 | 5.71E-06 | + |
|  |  |  |  |  |  |  | Whole blood | CXCL9 | 2.92E-06 | + |
|  |  |  |  |  |  |  | Whole blood | GATAD2A | 1.05E-115 | - |
|  |  |  |  |  |  |  | Whole blood | LPAR2 | 1.52E-29 | - |
|  |  |  |  |  |  |  | Whole blood | MAU2 | 3.27E-310 | + |
|  |  |  |  |  |  |  | Whole blood | MEF2B | 1.70E-22 | + |
|  |  |  |  |  |  |  | Whole blood | MEF2BNB | 2.62E-08 | + |
|  |  |  |  |  |  |  | Whole blood | PHF19 | 2.28E-10 | - |
|  |  |  |  |  |  |  | Whole blood | RUNDC3A | 1.98E-10 | + |
|  |  |  |  |  |  |  | Whole blood | TPST1 | 5.48E-06 | + |
|  |  |  |  |  |  |  | Whole blood | YJEFN3 | 5.00E-13 | - |
|  |  |  |  |  |  |  | Whole blood | ZNF486 | 2.26E-07 | + |
| rs7029757 (TOR1B) | A | G | rs3816260 | C | T | 0.73 | Adipose subcutaneous | C9orf78 | 3.83E-07 | - |
|  |  |  | rs146693094 | G | C | 0.69 | Colon transverse | C9orf78 | 2.88E-06 | - |
|  |  |  | rs35595055 | G | C | 0.60 | T cells | C9orf78 | 1.46E-12 | - |
|  |  |  |  |  |  |  | Whole blood | C9orf78 | 3.27E-310 | - |
|  |  |  |  |  |  |  | Whole blood | C9orf78 | 2.01E-72 | - |
|  |  |  |  |  |  |  | Whole blood | FNBP1 | 5.98E-15 | - |
|  |  |  |  |  |  |  | Whole blood | LINC00597 | 4.03E-06 | - |
|  |  |  |  |  |  |  | Whole blood | PTGES | 1.43E-09 | - |

|  |  |  |  |  |  |  | Neutrophils | TOR1A | 1.78E-11 | - |
| --- | --- | --- | --- | --- | --- | --- | --- | --- | --- | --- |
|  |  |  |  |  |  |  | Whole blood | TOR1A | 8.74E-84 | - |
|  |  |  |  |  |  |  | Colon transverse | TOR1B | 1.90E-15 | - |
|  |  |  |  |  |  |  | Liver | TOR1B | 5.77E-26 | NA |
|  |  |  |  |  |  |  | Monocytes | TOR1B | 1.92E-11 | - |
|  |  |  |  |  |  |  | Neutrophils | TOR1B | 1.76E-17 | - |
|  |  |  |  |  |  |  | Small intestine terminal ileum | TOR1B | 2.64E-06 | - |
|  |  |  |  |  |  |  | Visceral abdominal fat | TOR1B | 8.81E-16 | NA |
|  |  |  |  |  |  |  | Whole blood | TOR1B | 3.27E-310 | - |
|  |  |  |  |  |  |  | Whole blood | TPST2 | 2.60E-22 | - |
|  |  |  |  |  |  |  | Whole blood | USP20 | 2.27E-09 | - |
| rs134489 (TTC28) | A | T | rs695659 | G | A | 0.98 | Whole blood | CCDC117 | 1.28E-10 | + |
|  |  |  | rs134548 | G | A | 0.97 | Whole blood | TRIM68 | 8.76E-06 | + |
|  |  |  | rs134500 | C | T | 0.98 | Whole blood | TTC28 | 4.38E-18 | + |
|  |  |  | rs695625 | G | A | 0.98 | Whole blood | TTC28-AS1 | 2.10E-08 | + |
|  |  |  |  |  |  |  | Whole blood | XBP1 | 1.47E-61 | + |
| rs998584 (VEGFA, LINC01512) | C | A | rs11967262 | C | G | 0.98 | Whole blood | PEX6 | 1.92E-12 | - |
|  |  |  | rs4711750 | T | A | 0.96 | Whole blood | KRT80 | 4.15E-06 | + |
|  |  |  | rs1358980 | C | T | 0.87 |  |  |  |  |
|  |  |  | rs6905288 | G | A | 0.70 |  |  |  |  |
| rs13212562 (VN1R10P, ZNF204P) | A | G | rs13212562 | A | G | 1.00 | Whole blood | BTN2A1 | 1.53E-25 | + |
|  |  |  |  |  |  |  | Whole blood | BTN2A2 | 2.48E-27 | - |
|  |  |  |  |  |  |  | Whole blood | BTN2A3P | 2.33E-13 | - |
|  |  |  |  |  |  |  | Whole blood | BTN3A1 | 2.34E-11 | + |
|  |  |  |  |  |  |  | Adipose subcutaneous | BTN3A2 | 4.90E-15 | + |
|  |  |  |  |  |  |  | Adipose visceral omentum | BTN3A2 | 7.57E-14 | + |
|  |  |  |  |  |  |  | Colon sigmoid | BTN3A2 | 4.12E-08 | + |
|  |  |  |  |  |  |  | Colon transverse | BTN3A2 | 1.78E-12 | + |
|  |  |  |  |  |  |  | Liver | BTN3A2 | 4.79E-06 | + |
|  |  |  |  |  |  |  | Monocytes | BTN3A2 | 7.54E-16 | + |

| Small intestine terminal ileum | BTN3A2 | 5.72E-07 | + |
| --- | --- | --- | --- |
| Spleen | BTN3A2 | 1.10E-11 | + |
| T cells | BTN3A2 | 8.41E-14 | + |
| Whole blood | BTN3A2 | 3.27E-310 | + |
| Lymphoblastoid cell lines | BTN3A3 | 5.82E-06 | NA |
| Whole blood | CTA-14H9.5 | 4.68E-07 | + |
| Whole blood | GABBR1 | 1.53E-10 | - |
| Whole blood | GUSBP2 | 4.86E-06 | - |
| Whole blood | HCG11 | 2.08E-26 | + |
| Whole blood | HCG4; HLA-P | 2.32E-07 | + |
| Whole blood | HCP5 | 8.26E-13 | + |
| Whole blood | HIST1H2BD | 1.25E-09 | + |
| Whole blood | HIST1H2BK | 1.81E-21 | - |
| Whole blood | HIST1H4K | 1.13E-12 | + |
| Monocytes | HIST1H4L | 3.65E-11 | + |
| T cells | HIST1H4L | 1.51E-19 | + |
| Whole blood | HLA-DQA2; HLA-DQA1 | 3.83E-10 | + |
| Whole blood | HLA-DRB6 | 4.44E-22 | - |
| Whole blood | HLA-F-AS1 | 2.32E-31 | - |
| Whole blood | LINC00243 | 1.81E-38 | - |
| Whole blood | PGBD1 | 1.32E-22 | - |
| Whole blood | PRRC2A | 1.53E-06 | + |
| Whole blood | PRSS16 | 7.04E-24 | - |
| Whole blood | RP11- 457M11.5 | 2.15E-06 | + |
| Whole blood | RP1-265C24.5 | 4.42E-18 | + |
| Whole blood | RP5-874C20.3 | 7.52E-46 | + |
| Whole blood | SLC44A4 | 4.24E-20 | - |
| Whole blood | TRIM10 | 7.96E-09 | + |
| Whole blood | TRIM26 | 1.35E-19 | - |
| Whole blood | TUBB | 2.01E-38 | - |
| Monocytes | ZKSCAN4 | 7.43E-06 | - |

|  |  |  |  |  |  |  | Neutrophils | ZKSCAN4 | 2.28E-11 | - |
| --- | --- | --- | --- | --- | --- | --- | --- | --- | --- | --- |
|  |  |  |  |  |  |  | Whole blood | ZKSCAN4 | 5.67E-73 | - |
|  |  |  |  |  |  |  | Neutrophils | ZKSCAN8 | 1.12E-07 | - |
|  |  |  |  |  |  |  | Neutrophils | ZNF165 | 1.75E-07 | - |
|  |  |  |  |  |  |  | Whole blood | ZNF165 | 4.58E-17 | - |
|  |  |  |  |  |  |  | Whole blood | ZNF192 | 1.43E-14 | - |
|  |  |  |  |  |  |  | Whole blood | ZNF204P | 1.70E-12 | - |
|  |  |  |  |  |  |  | Whole blood | ZNF391 | 1.08E-07 | - |
|  |  |  |  |  |  |  | Whole blood | ZSCAN12P1 | 1.91E-24 | - |
|  |  |  |  |  |  |  | Neutrophils | ZSCAN16 | 8.10E-07 | - |
|  |  |  |  |  |  |  | Whole blood | ZSCAN16 | 1.34E-63 | - |
|  |  |  |  |  |  |  | Monocytes | ZSCAN16-AS1 | 8.59E-06 | - |
| rs17743415 (ZNF638) | C | T | rs142333215 | T | TA G T C | 0.99 | Whole blood | CLEC4F | 3.70E-07 | - |
|  |  |  | rs17693408 | A | G | 0.99 | Monocytes | DYSF | 6.94E-12 | - |
|  |  |  | rs67817805 | G | A | 0.98 | Whole blood | DYSF | 9.94E-36 | - |
|  |  |  | rs3771371 | T | C | 0.98 | Whole blood | MCEE | 6.59E-44 | + |
|  |  |  |  |  |  |  | Whole blood | MPHOSPH10 | 2.26E-06 | + |
|  |  |  |  |  |  |  | Whole blood | NAGK | 5.81E-18 | + |
|  |  |  |  |  |  |  | T cells | ZNF638 | 3.86E-24 | - |
|  |  |  |  |  |  |  | Whole blood | ZNF638 | 4.42E-161 | - |

Gene expression results in whole blood and immune cells and in liver, adipose and gut tissues were extracted from PhenoScanner. a1: effect allele, a2: non-effect allele, r2: between the input variant and the proxy variant, based on AMR from 1000 Genomes. The direction of association with respect to the effect allele is shown.
