# Supplementary material for: Genetic variants associated with circulating liver injury markers in Mexican Americans, a population at risk for non-alcoholic fatty liver disease
Source: Front Genet. 2022 Oct 26;13:995488. doi: 10.3389/fgene.2022.995488 (PMC9644071; doi:10.3389/fgene.2022.995488)
Supplement: Supplementary file 3 [file DataSheet1.DOCX]

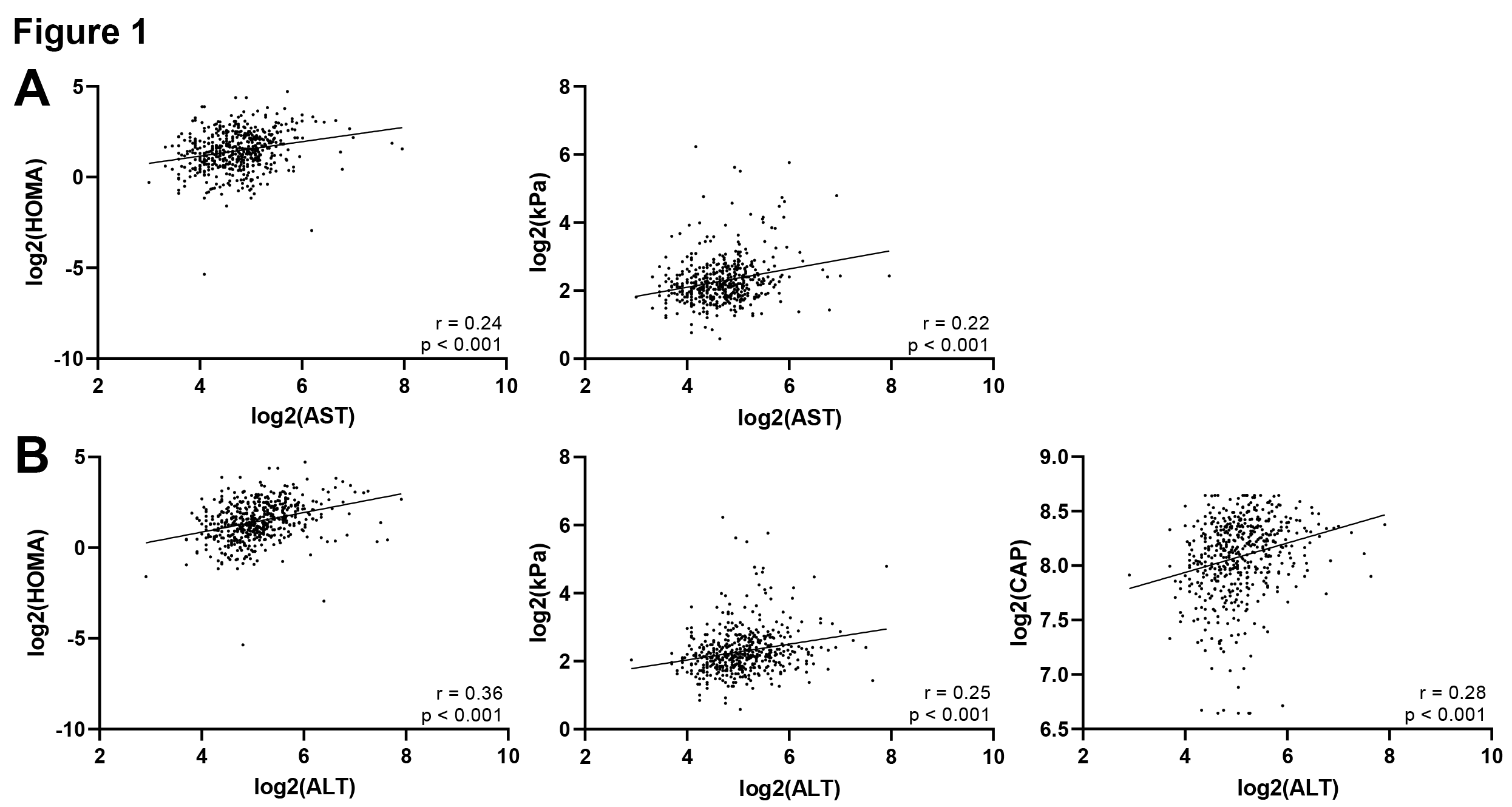


**Supplementary Figure S1.** Spearman's correlations between AST and ALT levels and clinical parameters. (A) Positive correlation between AST levels and HOMA or liver fibrosis (FibroScan kPa). (B) Positive correlation between ALT levels and HOMA, liver fibrosis (FibroScan kPa) or steatosis (FibroScan CAP).

**
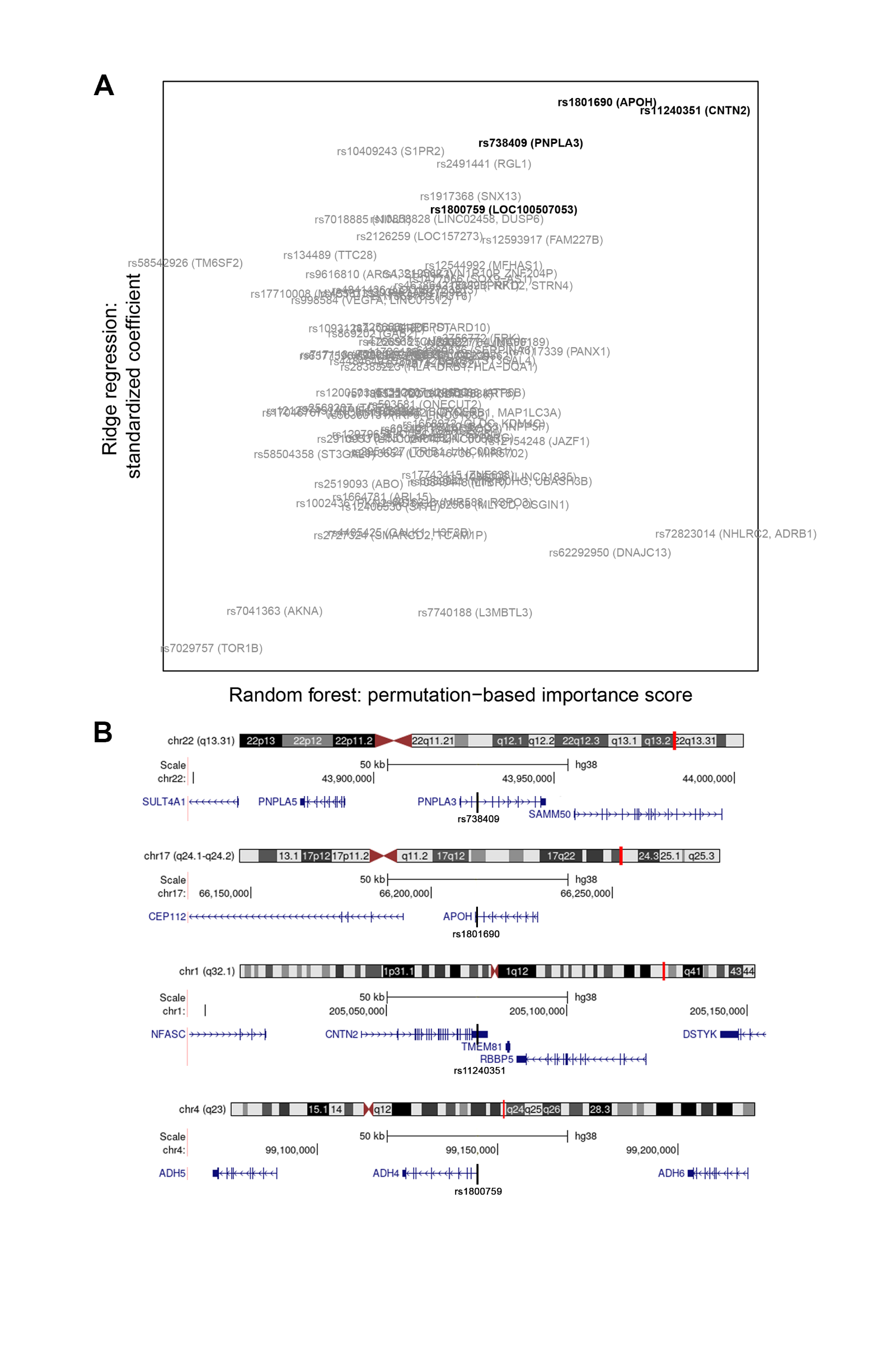
**

**Supplementary Figure S2.** Contribution of variants in predicting risk of advanced fibrosis. For the 86 AST- and ALT-associated variants, random forest and ridge regression were implemented to determine which variants contributed the most to predicting risk of advanced fibrosis. (A) For each variant, unscaled permutation-based importance scores from random forest were plotted against the standardized coefficients from ridge regression. Variants in black and bold were ranked among the top 10 most important contributors by both machine learning algorithms. (B) Genomic context of rs738409 in *PNPLA3*, rs1801690 in *APOH*, rs11240351 in *CNTN2* and rs1800759 in *LOC100507053*. For each variant, the chromosomal location is marked in red on the chromosome ideogram. Below each chromosome ideogram, the overlapping and surrounding genes within 80k base pairs upstream and downstream of each variant are shown.


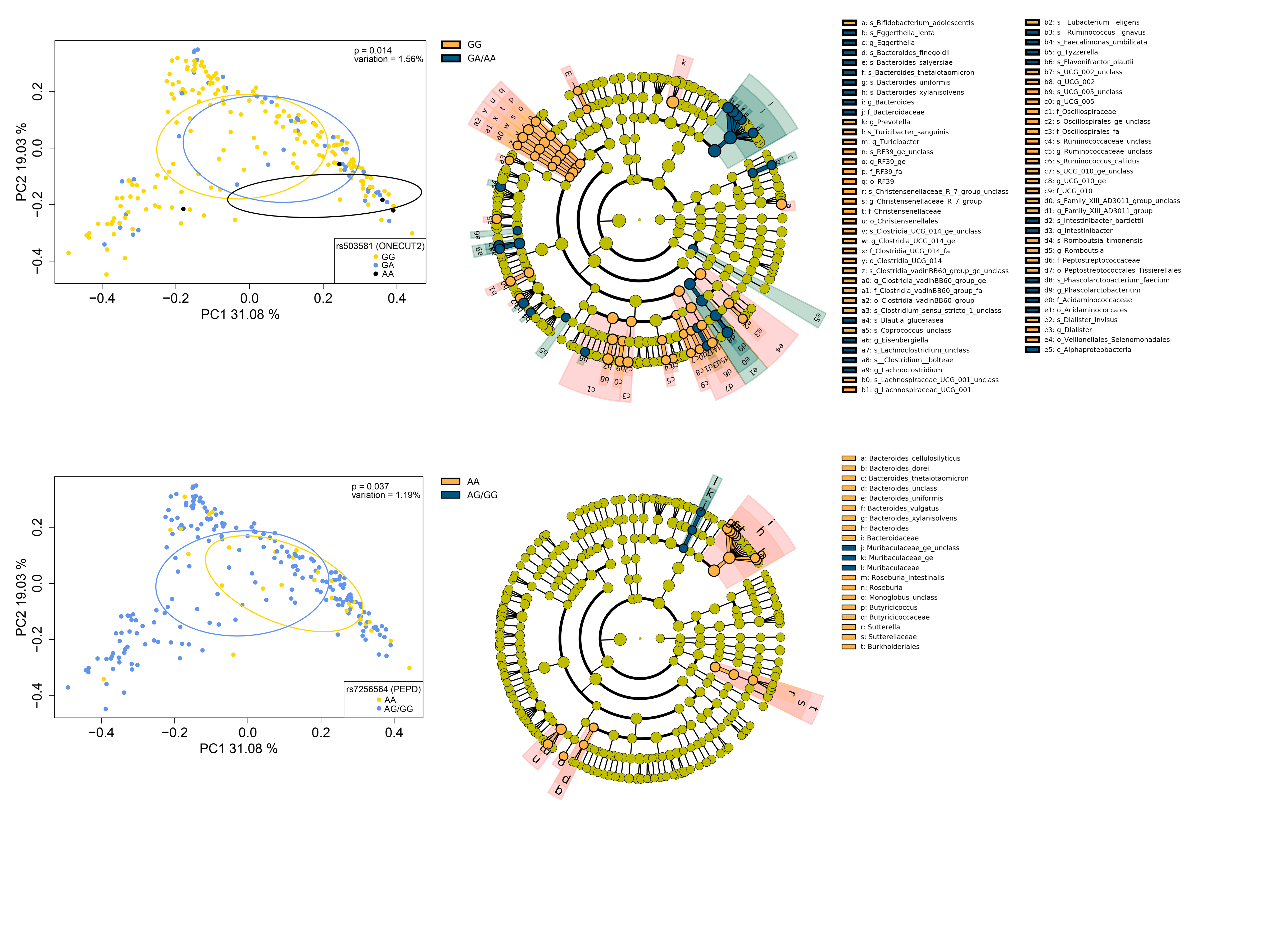


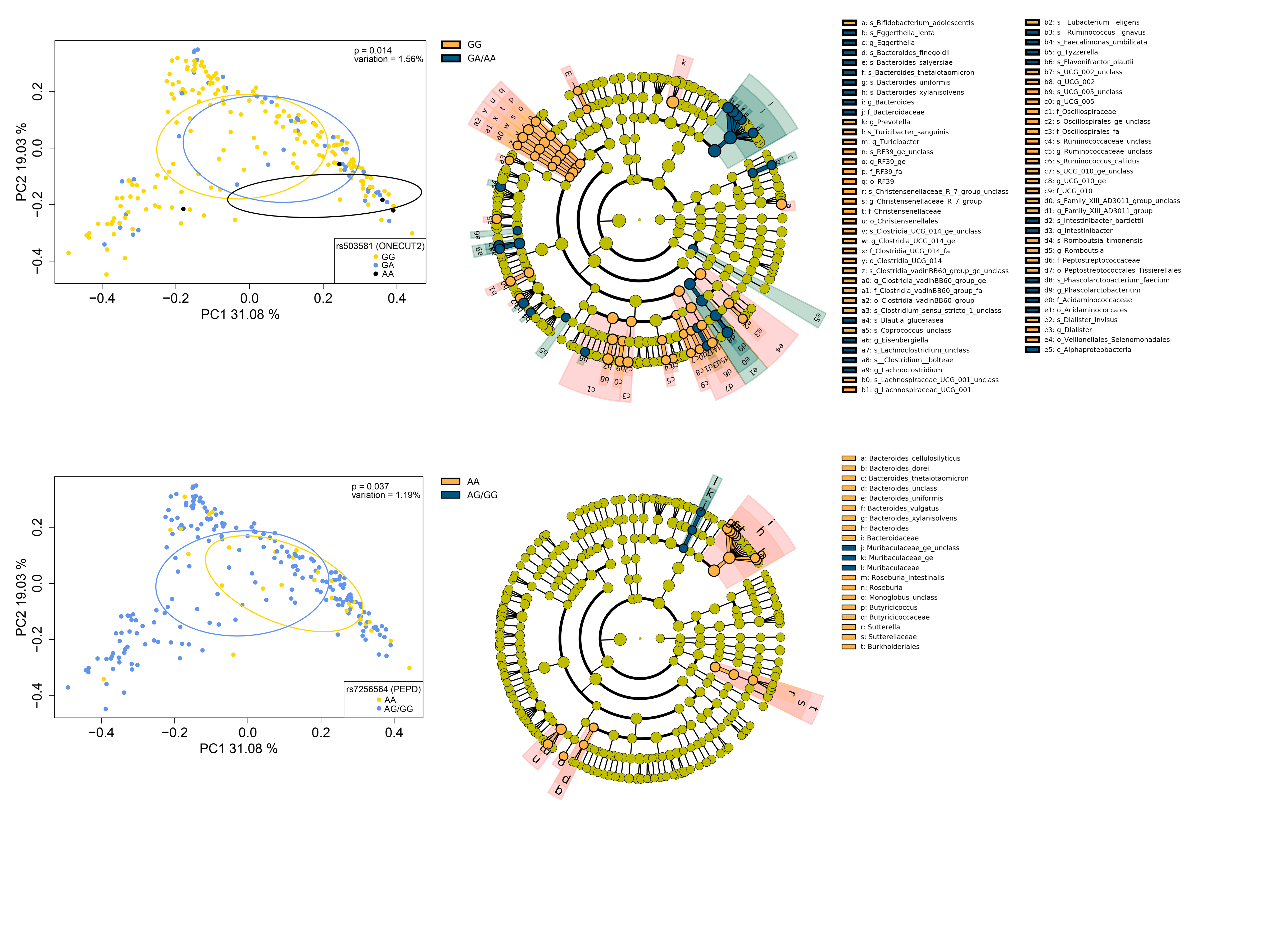

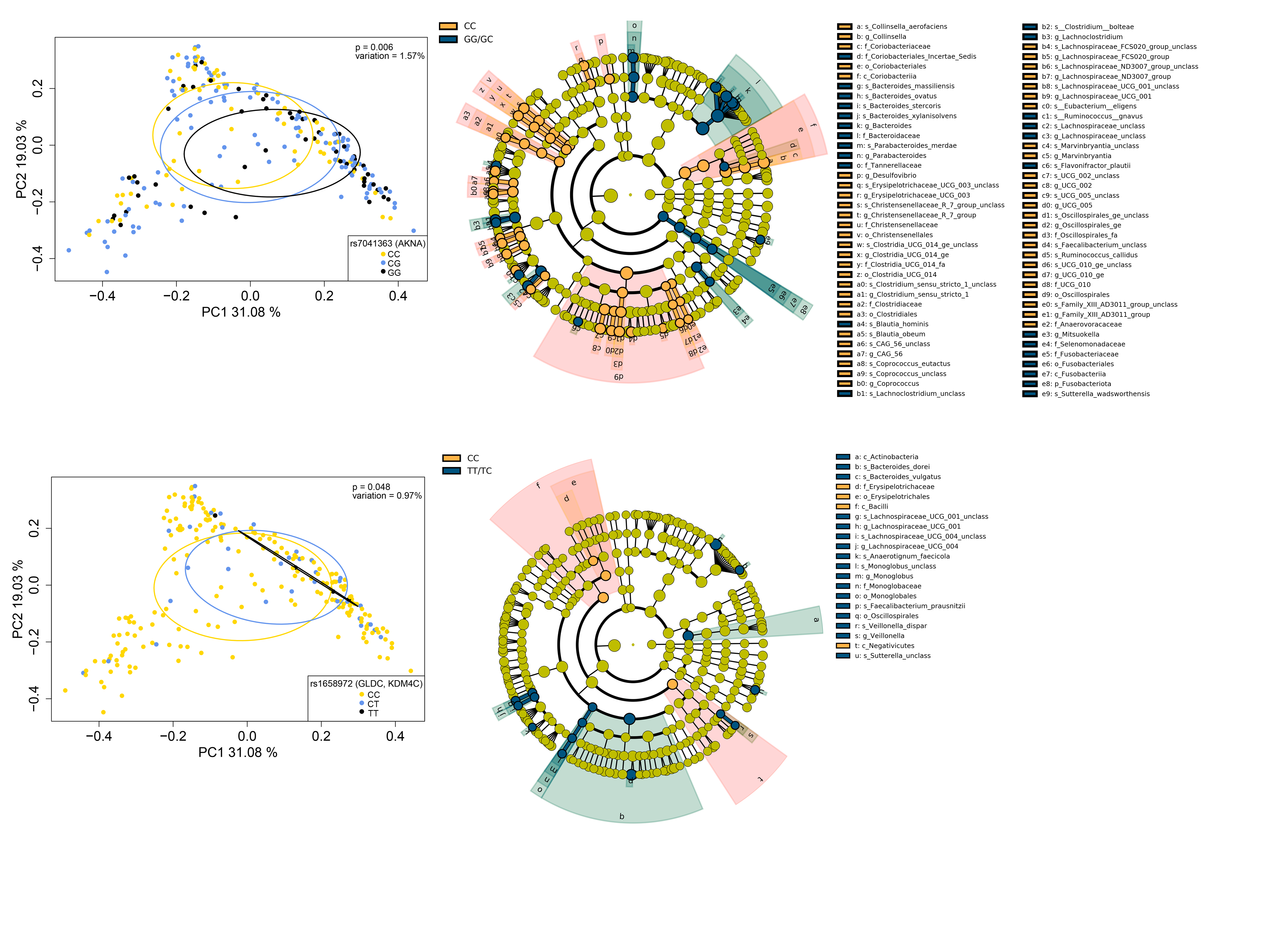


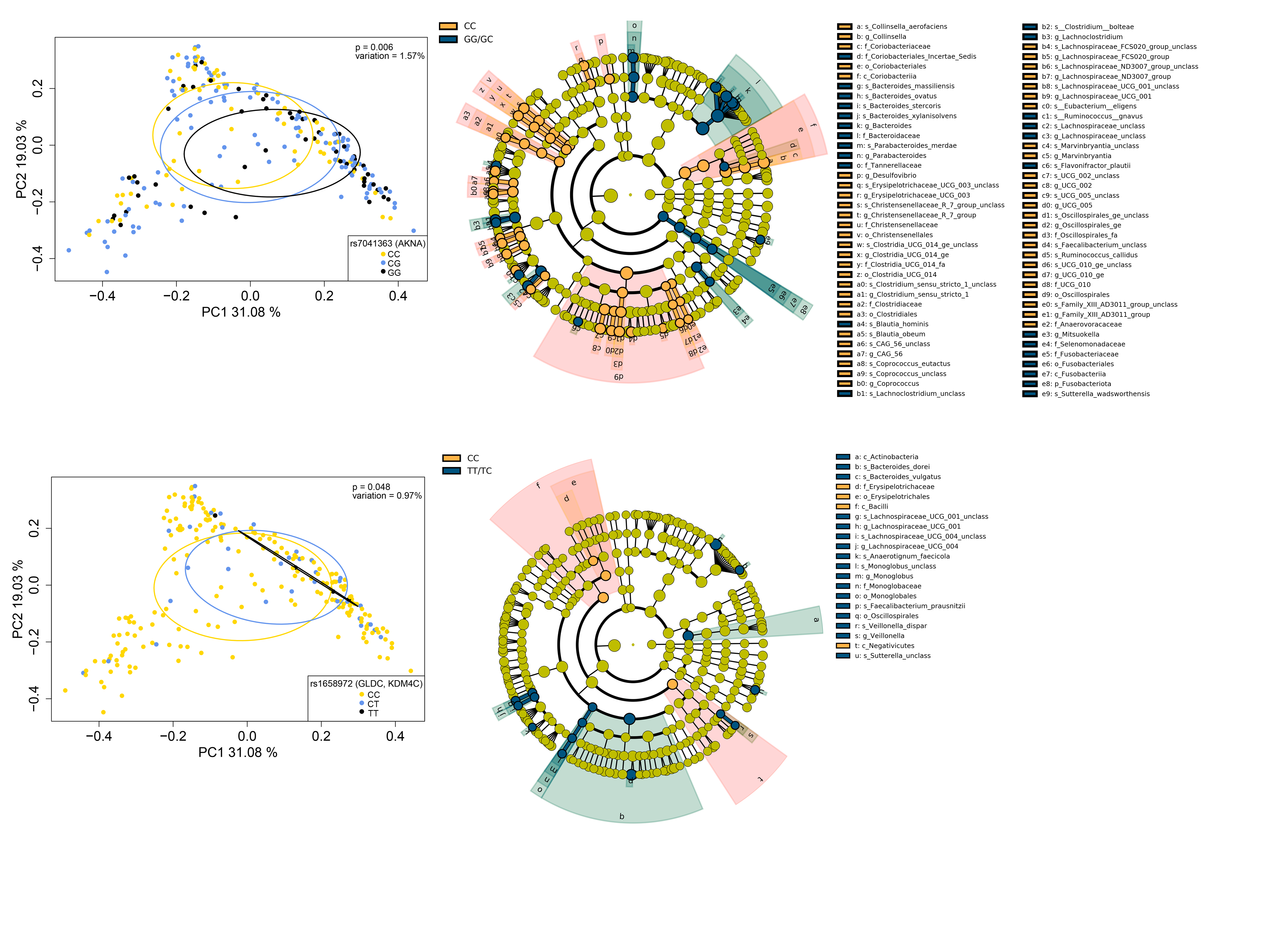


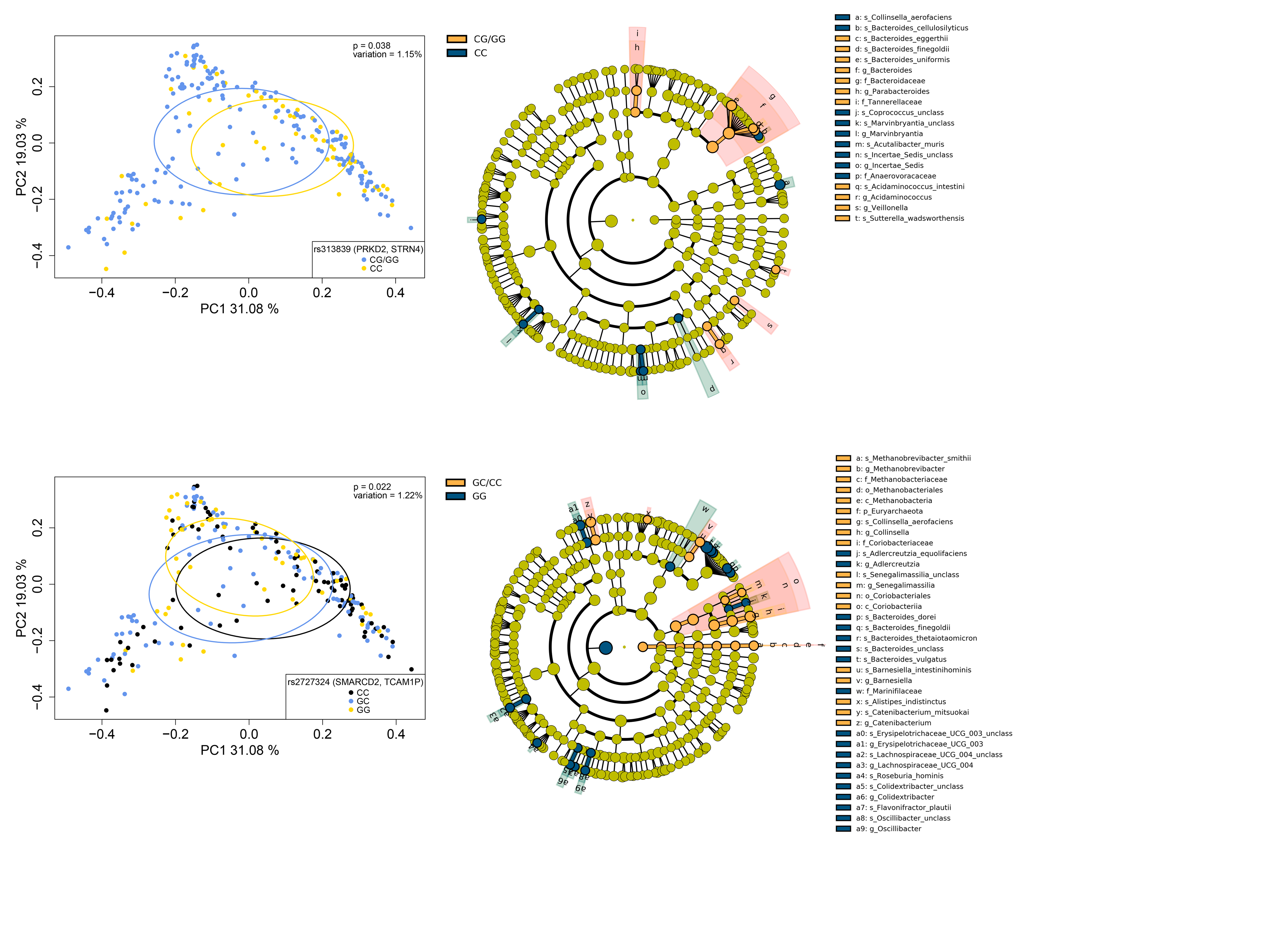


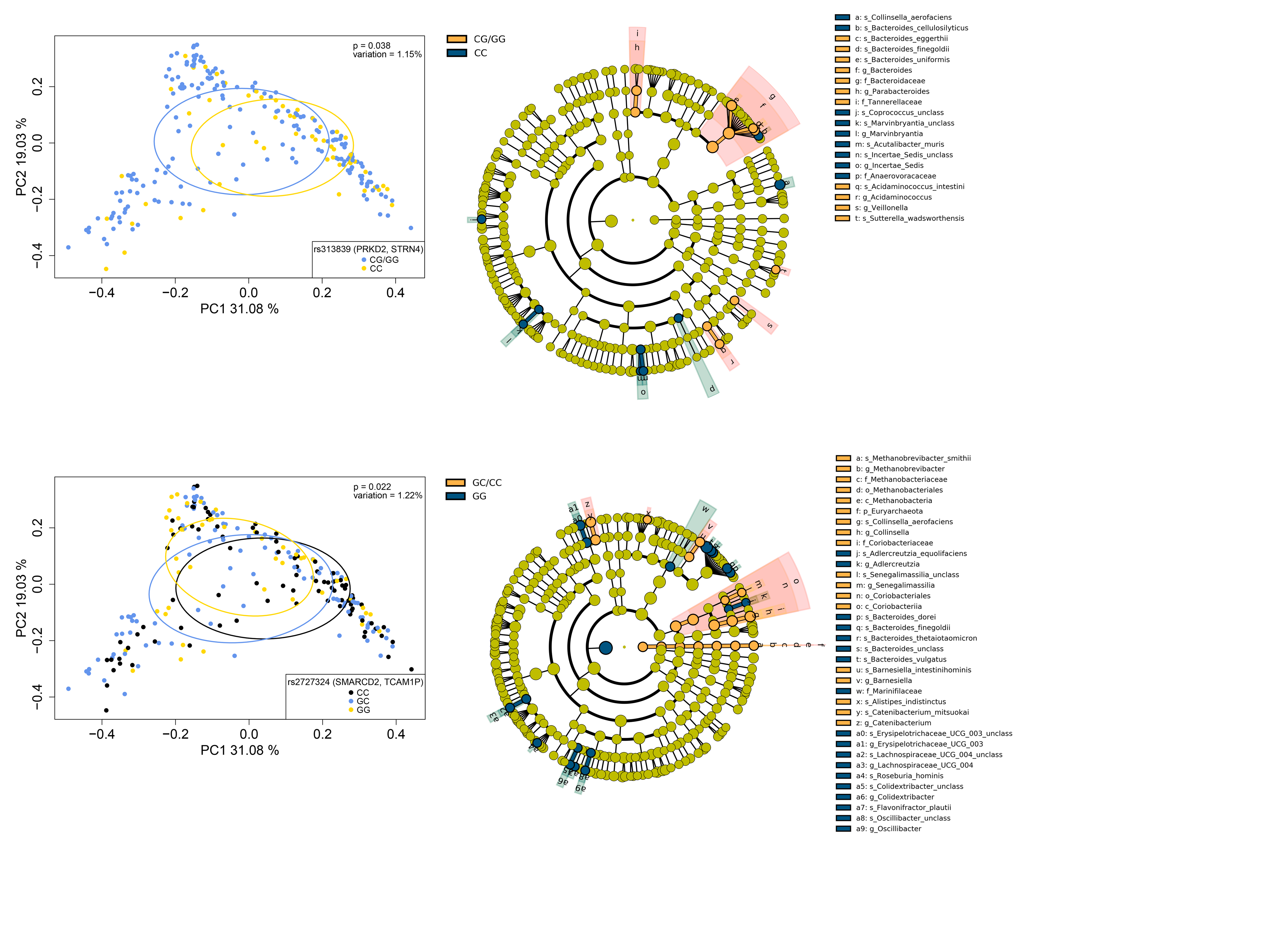


**Supplementary Figure S3.** Variants associated with gut microbiome changes. In addition to the variants shown in Figure 7, six additional variants were shown to be associated with significant changes in gut microbiome profiles. Left: PCoA plots of microbiome profiles by genotypes based on weighted UniFrac distances. Right: cladograms showing taxa with significantly different abundances between genotypes assessed by the LEfSe algorithm.
